# Targeting the 16S rRNA Gene for Bacterial Identification in Complex Mixed Samples: Comparative Evaluation of Second (Illumina) and Third (Oxford Nanopore Technologies) Generation Sequencing Technologies

**DOI:** 10.3390/ijms21010298

**Published:** 2019-12-31

**Authors:** Raf Winand, Bert Bogaerts, Stefan Hoffman, Loïc Lefevre, Maud Delvoye, Julien Van Braekel, Qiang Fu, Nancy HC Roosens, Sigrid CJ De Keersmaecker, Kevin Vanneste

**Affiliations:** Transversal activities in Applied Genomics, Sciensano, 1050 Brussels, Belgium; raf.winand@sciensano.be (R.W.); bert.bogaerts@sciensano.be (B.B.); stefan.hoffman@sciensano.be (S.H.); loic.lefevre@sciensano.be (L.L.); maud.delvoye@sciensano.be (M.D.); julien.vanbraekel@sciensano.be (J.V.B.); qiang.fu@sciensano.be (Q.F.); nancy.roosens@sciensano.be (N.H.C.R.)

**Keywords:** orientation, bacterial identification, 16S rRNA, targeted genomics, public health, Illumina, Nanopore, targeted metagenomics

## Abstract

Rapid, accurate bacterial identification in biological samples is an important task for microbiology laboratories, for which 16S rRNA gene Sanger sequencing of cultured isolates is frequently used. In contrast, next-generation sequencing does not require intermediate culturing steps and can be directly applied on communities, but its performance has not been extensively evaluated. We present a comparative evaluation of second (Illumina) and third (Oxford Nanopore Technologies (ONT)) generation sequencing technologies for 16S targeted genomics using a well-characterized reference sample. Different 16S gene regions were amplified and sequenced using the Illumina MiSeq, and analyzed with Mothur. Correct classification was variable, depending on the region amplified. Using a majority vote over all regions, most false positives could be eliminated at the genus level but not the species level. Alternatively, the entire 16S gene was amplified and sequenced using the ONT MinION, and analyzed with Mothur, EPI2ME, and GraphMap. Although >99% of reads were correctly classified at the genus level, up to ≈40% were misclassified at the species level. Both technologies, therefore, allow reliable identification of bacterial genera, but can potentially misguide identification of bacterial species, and constitute viable alternatives to Sanger sequencing for rapid analysis of mixed samples without requiring any culturing steps.

## 1. Introduction

Clinical microbiology laboratories and public health authorities have relied, traditionally, on phenotypic methods to identify bacterial pathogens, but these cannot be used for uncultivable bacteria. Slow-growing bacteria and bacteria with uncommon phenotypes can also render identification arduous and time-consuming [1]. A potential alternative is the use of 16S rRNA gene sequencing, which has historically been used to identify known and new bacteria independently of cultivability and phenotype [2,3]. The 16S rRNA gene is approximately 1500 bp long and consists of nine (hyper)variable regions named V1 to V9, interspaced with more conserved regions. Since the 16S rRNA gene is present in all bacteria and subject to different evolutionary rates depending on the gene region considered, it has historically been used for classification of isolates [4], and more recently in complex samples coming from a variety of environments, such as the human gut [5], soil [6], and oceans [7] where it can also be used to determine the bacterial number and composition of samples. Employing 16S rRNA gene sequencing for bacterial identification is, however, impacted by several factors, such as the 16S rRNA gene region(s) considered and their amplification efficiency, the sequencing technology itself, and the bioinformatics workflow(s) employed.

Regarding the 16S rRNA gene region(s) considered, the likelihood of correct species identification varies substantially between genera and species based on their sequence similarity [8], because the 16S rRNA gene has poor discriminatory power at the species level, and even at the genus level for certain bacterial clades [9]. Systematic investigations have indicated that up to 90%, and 86%, of bacteria can be reliably identified at the genus and species levels, respectively [10], because the variable 16S regions that are ultimately used for classification are not all equally informative between and across different species, genera, and families [11,12]. Another complicating factor, especially in mixed samples, is 16S rRNA gene copy number variation, ranging from one copy in *Mycoplasma pneumoniae*, over seven copies in *Escherichia coli*, to up to 15 copies in *Bacillus subtilis* [13,14,15], potentially causing species with low copy numbers to remain undetected. Sequence variations between multiple 16S rRNA gene copies can also exist within the same species. *E. coli* (ATCC 70096) has seven copies of which six are different over the full gene length, but all seven are identical when only considering the V4 region. *Staphylococcus aureus* (ATCC BAA-1718) and *S. epidermidis* (ATCC 12228) contrarily have five gene copies, of which four are identical in their V4 region between both species, and the one remaining copy differs only by one nucleotide within the *S. aureus* copies, 1.7% within the *S. epidermidis* copies, and 2.0% between the *S. epidermidis* and *S. aureus* copies [16]. Several studies have attempted to identify the best region, or combination of regions, for classification, but resulted in different recommendations and preferences [17]. The PCR-step required to target and amplify 16S rRNA gene region(s) constitutes an additional concern. Although the primers employed are supposedly universal, in practice, 16S rRNA gene variability (even in conserved regions) can result in primer-template mismatches that may bias amplification and potentially impact identification, especially for mixed samples containing several bacteria [18], and primers performing well in silico might not work well in laboratory conditions [19].

Regarding sequencing technology, 16S rRNA sequences are traditionally generated using “first-generation” Sanger sequencing offering high basecalling accuracy, but constituting a difficult and laborious process when dealing with identification of bacterial communities, as this technology cannot be directly applied to mixed samples. Doing so would result in overlapping signals because all bacteria are sequenced, therefore, requiring an intermediate culturing step to first isolate the different bacteria [20]. With the advent of “next-generation” sequencing (NGS) technologies, it has become easier and more cost-effective to generate 16S rRNA gene sequences from organisms in mixed complex samples without intermediate culturing steps because of the high sequence data throughput generated in one reaction [21]. Several high-throughput sequencing (HTS) technologies are currently available, each with their unique advantages and disadvantages, allowing one to generate increasing quantities of data at decreasing costs [22]. Consequently, HTS technologies are being adopted in diverse applications to allow rapid identification of bacteria of clinical interest [23,24,25]. “Second-generation” NGS technologies, such as Illumina technology, are also referred to as “short-read” sequencing technologies because achievable read lengths are relatively limited, but vast quantities of accurate sequencing data or “reads” can be generated through their massively parallel set-up. The Illumina MiSeq instrument is capable of sequencing maximum 2 × 300 bp, which is insufficient to cover the entire 16S rRNA gene, but does allow sequencing one or more variable regions, rendering gene region selection paramount due to variable gene regions differences within and between species, as discussed previously [17]. To circumvent this limitation, a consensus approach can be employed based on combining information from multiple variable regions. This approach is, for instance, employed by Life Technologies, who developed a 16S rRNA gene sequencing protocol for their Ion PGM System [26], but comprehensive investigation of the performance of such an approach is currently largely absent. More recently, a new generation of sequencing technologies based on single molecule sequencing has appeared, also referred to as “third-generation” NGS technologies, which are capable of producing much longer reads. The tradeoff in regard to second-generation NGS technologies is that due to the absence of a massively parallel sequencing set-up, basecalling accuracy is lower, resulting in higher read error rates. Although available since ≈2010, as offered by Pacific Biosciences (PacBio), this technology has only recently become widely accessible through the launch of the MinION instrument by Oxford Nanopore Technologies (ONT), which can generate reads of more than 2 Mb and is considered a disruptive innovation within microbial genomics through its portability, possibility to perform “real-time” data analysis, and lower cost per quantity of sequenced bases [27,28,29]. This allows sequencing the entire 16S rRNA gene, and several recent case studies have highlighted its potential for rapid accurate bacterial identification [24,30,31]. Its higher read error rate of ≈8–15% [32,33,34] compared to ≈0.1% for the Illumina MiSeq [35], does however hinder identification because it is higher than thresholds used for 16S rRNA-based classification where most taxonomists require percent identity scores of ≥97% and ≥99% to classify bacteria to genera and species, respectively [36].

Regarding bioinformatic analysis, multiple workflows exist. For short-read data, community standards and guidelines have emerged and are now readily available. Mothur, an open-source and multiplatform software for sequence-based microbial community analysis [37], maintains a standard operating procedure (SOP) for Illumina Miseq data (https://www.mothur.org/wiki/MiSeq_SOP) that has been used as basis for other community guidelines [38]. Such recommendations are, however, still largely absent for ONT data, although several bioinformatics tools and packages already support long error-prone reads [39,40,41,42]. Mothur also allows ONT data analysis, although a SOP does not exist (yet). The online EPI2ME platform (https://epi2me.nanoporetech.com) is a proprietary solution provided by ONT, containing a 16S workflow optimized for analyzing MinION reads. Alternatives optimized to deal with long error-prone reads, such as GraphMap [42], also exist. All the aforementioned tools and packages require 16S gene bacterial reference databases containing sequence information to allow classification. The SILVA database constitutes one of the largest and most actively maintained and employed databases containing comprehensive, curated 16S rRNA gene sequences [43,44]. A curated collection of 16S rRNA sequences and taxonomic information is also maintained in NCBI. Importantly, amplification and sequencing of 16S rRNA gene (regions), and bioinformatic analysis of generated sequencing data, constitute two separate aspects that are, nevertheless, heavily intertwined. Issues with amplification and sequencing, such as PCR artifacts and read error rates, can influence classification through propagation of wrong or biased sequence information. However, even when obtaining “perfect sequences,” the 16S rRNA gene might not offer enough discriminatory power to distinguish between certain bacteria present in a sample, especially if they are closely related.

The discriminatory power of selected 16S rRNA gene region(s) and their amplification efficiency, sequencing technology (artifacts), and bioinformatics workflow, all interplay and affect classification, but comprehensive investigation of 16S rRNA-targeted genomics performance with different NGS technologies and bioinformatics approaches is still largely absent. We compared 16S rRNA-targeted genomic performance with both the Illumina and ONT technologies using a well-characterized bacterial reference sample (mock community) containing eight bacterial species as a proxy for a real-life biological sample. Several 16S rRNA gene regions were amplified and sequenced using the MiSeq, whereas the entire gene was amplified and sequenced using the MinION. We employed different reference databases and bioinformatics approaches for bacterial identification, and demonstrated that both technologies have their own advantages and disadvantages;both can both be used for accurate classification down to the genus level but not the species level.

## 2. Results

### 2.1. Classification of a Well-Characterized Reference Sample Through Sequencing Different 16S rRNA Gene Regions with the Illumina (MiSeq) Technology

**Figure 1:**
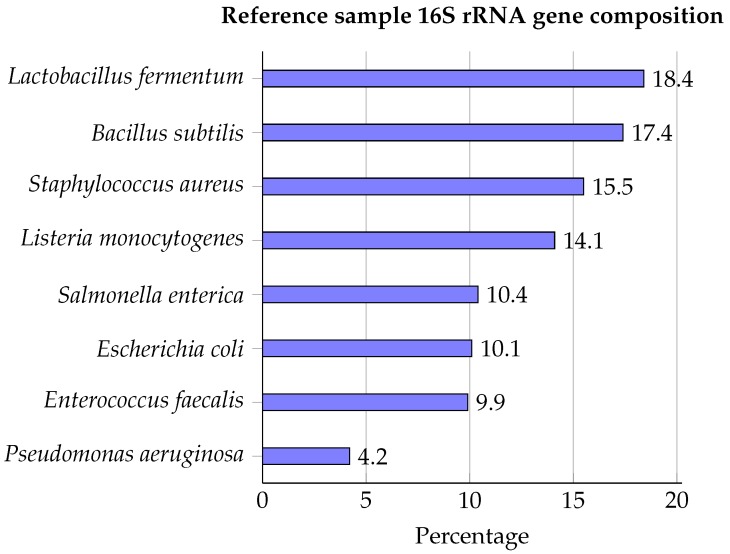
Bacterial composition of the ZymoBIOMICS™ Microbial Community DNA Standard expressed as 16S rRNA gene percentages [45].

The ZymoBIOMICS™ Microbial Community DNA Standard was used as reference material containing genomic DNA of eight bacterial species (*Lactobacillus fermentum*, *Bacillus subtilis*, *Staphylococcus aureus*, *Listeria monocytogenes*, *Salmonella enterica*, *Escherichia coli*, *Enterococcus faecalis*, and *Pseudomonas aeruginosa*), for which 16S rRNA compositions are presented in Figure 1.

A literature survey to identify different 16S rRNA gene variable regions, and primers for their amplification, was performed. Eleven amplicon libraries, hereafter referred to as “samples,” were created through amplification of different variable regions, or spanning multiple variable regions, for which an overview is presented in Table 1.

**Figure 2:**
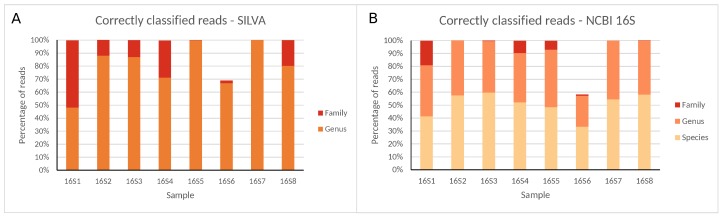
Correctly classified reads for all 16S rRNA gene regions at different taxonomic levels using the (**A**) SILVA and (**B**) NCBI 16S databases for Illumina MiSeq data. Percentages are expressed against the total number of reads classified at any taxonomic level (SILVA only allows classification until the genus).

Samples were sequenced on an Illumina MiSeq, after which Mothur was employed to analyze sequencing data following a community SOP, using either the SILVA or NCBI 16S reference databases that contain information down to the genus and species level, respectively (i.e., bacterial species could only be identified with the NCBI 16S database). Percentages of correctly classified reads per sample (i.e., assigned to bacteria present in the reference sample) at different taxonomic levels are presented in Figure 2, and percentages of reads correctly classified, misclassified (i.e., assigned to bacteria not present in the reference sample), and unclassified (i.e., not assigned), are presented in Table 2 for every taxonomic level (exact numbers are available in Table S2).

Large variation in raw read numbers between different regions was observed, with nearly no reads generated for samples 16S9, 16S10, and 16S11, and more than 500,000 reads for samples 16S1, 16S6, and 16S8, and other samples in between. Read numbers classified in samples 16S9, 16S10 and 16S11 were so low that no reads were correctly classified in samples 16S10 and 16S11, and only maximally 103 reads in sample 16S9, which were, therefore, excluded from further analysis and discussion. An extended description of read quality and statistics for all samples is presented in the Supplementary Information. For all samples and databases, ≤0.09% of reads were misclassified and ≤0.33% remained unclassified at the family level. At the genus level, the percentage of misclassified reads remained identical, while the fraction of unclassified reads increased, ranging between 0% and ≈52%. Sample 16S6 was an exception both at the family and genus level with many unclassified reads and few misclassified reads due to the low number of unique reads compared to total reads. At the species level, accurate classification using the NCBI 16S database was generally problematic, with all samples exhibiting large misclassified or unclassified read numbers, or both, ranging between ≈8% and ≈27%, and ≈17% and ≈67%, respectively (see Supplementary Information).

A detailed overview of reads identified for every bacterial species present in the reference sample is provided in Figures S1–S16, and a detailed overview of all bacterial reads identified for every sample is provided in Figures S17–S32. Depending on gene region and taxonomic level, estimated relative abundances could deviate substantially from real abundances for all bacteria, ranging from underestimation to large overestimation, especially in samples with lower quality reads, such as 16S6. Additionally, if classification was impossible at a certain taxonomic level for the present bacteria, their relative abundance would drop to zero, thereby impacting predicted relative abundances of other detected bacteria accordingly. This was especially apparent for species classification where predicted relative abundances of identified species expected to be present were typically over-inflated. An extended description of results for all bacteria of the reference sample is provided in the Supplementary Information. A non-template control library (NTC) was also prepared for each gene region and sequenced, but read numbers surviving pre-processing and being classified were generally low, with <350 reads classified at the family level. At the genus level, for bacteria present in the reference sample, only *Pseudomonas*, *Staphylococcus*, and *Bacillus* were identified in a limited number of NTC samples. For bacteria not part of the reference sample, the large majority were only sporadically identified belonging, amongst others, to the genera *Delftia*, *Bradyrhizobium*, *Sphingomonas*, *Actinomyces*, *Corynebacterium*, *Devosia*, *Enhydrobacter*, *Mesorhizobium*, *Methylobacterium*, *Micrococcus*, *Stenotrophomonas*, and *Streptococcus*. An extended description including all detected genera and species is provided in the Supplementary information.

Despite variation in correctly and misclassified read numbers, and detected species per sequenced 16S rRNA gene region, only sample 16S5 allowed us to unambiguously identify bacteria (i.e., only detect bacteria present, and not falsely detect bacteria absent). To improve identification accuracy, an alternative consensus approach was explored based on a majority vote over all gene regions combined to determine presence and absence of bacteria. As samples 16S9, 16S10, and 16S11 were omitted (see before), eight gene regions remained, for which results are presented in Table 3 and Table 4 for the SILVA and NCBI 16S databases, respectively.

At the family level, all expected families were detected, and very few false positives existed. The family *Neisseriaceae* was incorrectly identified in seven samples using both the SILVA and NCBI 16S databases, which could likely be explained by “bleed-through” during sequencing because another sample containing almost exclusively *Neisseria meningitidis* was included in the same sequencing run (no additional samples were however sequenced in the same run). The family *Carnobacteriaceae* was incorrectly identified only in sample 16S6 when using the SILVA database with no corresponding hits at the genus level. Because this is a sample of lower sequencing quality with very few unique reads (see above), this most likely constitutes a misidentification of *L. fermentum* because the *Lactobacillales* and *Carnobacteriaceae* belong to the same order: Lactobacillales. At the genus level, all expected genera were detected and false positives identifications remained fairly limited, for which an extended overview is presented in Table S4. The genus *Neisseria* was identified through bleed-through (see before), although at maximally 0.06% reads in any sample. Other false positive identifications occurred only when using the SILVA database in a minority of samples: *Azomonas* (four samples), *Trabulsiella* (three samples), *Melissococcus* (one sample), and *Falsibacillus* (one sample). Conversely, the genera *Pseudomonas*, *Salmonella*, and *Enterococcus* were only correctly identified in, respectively, four, two, and five samples using the SILVA database; and the genus *Salmonella* was only correctly identified in six samples when using the NCBI 16S database, indicating that false negatives also start appearing. At the species level, both false negative and positive identifications increased considerably, typically both within the same genus. *E.coli* was only correctly identified in sample 16S6, but *E. fergusonii* was incorrectly identified in six samples. *L. monocytogenes* was never correctly identified in any sample, whereas *L. welshimeri* was incorrectly identified in five samples. *B. subtilis* was never correctly identified in any sample, whereas *B. halotolerans* was incorrectly identified in one sample. *E. faecalis* was identified correctly in four samples, whereas *E. hirae* and *E. saccharolyticus* were each incorrectly identified in one sample. *Neisseria polysaccharea* (four samples) and *N. weaveri* (one sample) were incorrectly identified through bleed-through (see before). Lastly, *P. aeruginosa*, *S. enterica*, *S. aureus*, and *L. fermentum* were correctly identified in respectively seven, six, eight, and eight samples.

### 2.2. Classification of a Well-Characterized Reference Sample through Sequencing the Entire 16S rRNA Gene with the ONT (MinION) Technology

**Figure 3:**
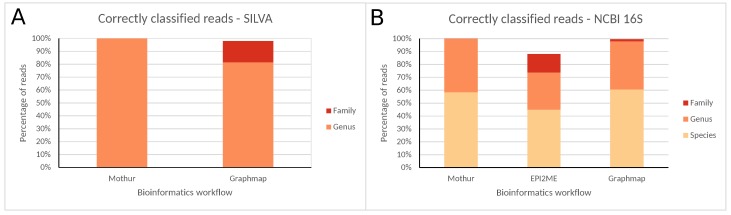
Correctly classified reads for all bioinformatics workflows at different taxonomic levels using the (**A**) SILVA and (**B**) NCBI 16S databases for ONT MinION data. Percentages are expressed against the total number of reads classified at any taxonomic level (SILVA only allows classification until the genus, and EPI2ME only supports the NCBI 16S database).

The same reference material was used, but only one library was created through amplification of the 16S rRNA gene over its entire length, and afterwards sequenced on an ONT MinION. Data were down-sampled to 10,000 reads to accommodate computational resources required for data analysis with three different bioinformatic workflows [34]: Mothur, EPI2ME, and GraphMap. The SILVA and NCBI 16S reference databases allowing classification down to the genus and species level, respectively, were used for Mothur and GraphMap, whereas EPI2ME only supported the NCBI 16S database. Classification results are presented in Figure 3 and Table 5 for all bioinformatics workflows.

A detailed overview of read numbers assigned to each taxonomic level using both databases for the different bioinformatics workflows is presented in Figures S33–S37 and Tables S5–S7. Similarly to MiSeq data, classification accuracy was largely dependent on the taxonomic level considered. At the family level, the majority of reads were correctly classified, and nearly no misclassifications occurred. Using the SILVA database, Mothur and GraphMap misclassified zero and 145 (2.04%) reads, and neither had any unclassified reads after pre-processing (results for EPI2ME are not available because it does not support SILVA). Using the NCBI 16S database, misclassified reads were similarly minimal with zero, 30 (0.34%), and 35 (0.45%), reads for Mothur, EPI2ME, and GraphMap, respectively. Both Mothur and GraphMap did not have any unclassified reads, whereas EPI2ME had 1135 (11.54%) unclassified reads. At the genus level, the majority of reads were again correctly classified, and misclassified reads remained limited. Using the SILVA database, Mothur did not misclassify any reads, while GraphMap misclassified 1311 reads (18.48%), with neither having any unclassified reads. Using the NCBI 16S database, Mothur misclassified only a single read (0.02%), while EPI2ME and GraphMap misclassified 68 (0.93%) and 163 (2.09%) reads, respectively. Both Mothur and GraphMap did not have any unclassified reads, whereas EPI2ME had 2505 (25.46%) unclassified reads. At the species level, the correctly classified reads reduced substantially, while misclassified reads increased drastically. Mothur, EPI2ME, and GraphMap misclassified 1836 (41.45%), 2897 (39.50%), and 3073 (39.34%) reads. Both Mothur and GraphMap did not have any unclassified reads, whereas EPI2ME had 2505 (25.46%) unclassified reads.

A detailed overview of all bacterial reads identified is provided in Figures S38–S42. At the family level, using Mothur for both databases, all expected families were detected, and no false positive identifications occurred. Using EPI2ME and GraphMap with either reference database, a small amount of false positive identifications supported by only very limited number of reads occurred (see Table S7). At the genus level, using Mothur for both databases, all expected genera were detected and only a single false positive identification of the genus *Enterobacter* using the NCBI 16S database occurred, supported, however, by only a single read. Using EPI2ME and GraphMap with either database, a small amount of false positive identifications supported by only limited read numbers occurred, although read numbers for false positive identifications for GraphMap using the SILVA database were sometimes pronounced (see Table S6). At the species level, however, false positives occurred even when using Mothur and increased substantially for all bioinformatics workflows. False negatives also appeared; for instance, *E. coli* was only detected by GraphMap and *L. monocytogenes* was not detected by Mothur. Using read count thresholds to determine identification was explored, but did not allow increasing identification accuracy. If only the species with the highest read count per genus would be considered, *P. aeruginosa*, *S. enterica*, *L. fermentum*, *E. faecalis*, and *S. aureus*, would be classified correctly using Mothur, but not *L. monocytogenes*, *E. coli*, and *B. subtilis*. Moreover, such an approach would not be able to detect multiple species present of the same genus. An alternative approach based on enforcing an absolute reads number for determining presence and/or absence was not a viable option either. For instance, EPI2ME incorrectly identified both *B. halotolerans* and *B. mojavensis* with 357 and 397 reads, respectively, both higher numbers than the 179 reads supporting the *P. aeruginosa* identification. A detailed overview correctly classified reads for bacteria present in the reference sample at different taxonomic levels using the SILVA and NCBI 16S databases for the different bioinformatics workflows with respect to expected abundances is presented in Figures S38–S42, indicating that similarly to MiSeq data, predicted relative abundances do not correspond well with theoretically expected abundances. A trend of GC-low species being overestimated and GC-high species being underestimated could be observed. Since PCR primers from the MinION 16S amplification kit are known to exhibit some mismatches to the *Pseudomonas* 16S rRNA gene [54], the underestimation of this pathogen was especially pronounced.

## 3. Discussion

Rapid, accurate bacterial identification in (clinical) samples is important for orientation of biological samples for which 16S rRNA analysis by means of Sanger sequencing is often used, which is, however, limited by its inability to characterize bacteria in complex mixed samples without any intermediate culturing step(s). HTS technologies provide an interesting alternative to Sanger sequencing, because they do not require prior isolation [23,55,56], but comprehensive evaluation of their performance on reference standards remain largely absent. We investigated the performance of both second (Illumina) and third (ONT) generation NGS technologies for orientation by employing a well-characterized bacterial reference sample (see Figure 1). This reference sample has been used in other benchmarking studies as reference material for evaluating either the performance of short and long-read technologies for whole genome sequencing [34], or 16S targeted genomics based on PacBio long-read sequencing [57], and is, therefore, ideally suited for a comparative evaluation of the performance of short and long-read technologies for 16S-targeted genomics.

### 3.1. Employing Short-Read Second-Generation (Illumina) Sequencing to Characterize Different 16S rRNA Gene Regions

As short reads generated by the Illumina MiSeq do not allow sequencing the entire 16S rRNA gene, we performed a literature survey and selected 11 variable regions for amplification (see Table 1), after which resulting amplicons were sequenced and analyzed with the community-standard Mothur software tool suite [38,58,59,60], using both the SILVA [44,61] and NCBI 16S [61] reference databases that contain high-quality curated bacterial 16S rRNA gene sequences down to the genus and species level, respectively. Our results generally indicate large variability in classification accuracy between different variable regions. Gene regions spanning multiple variable regions resulted in higher classification accuracy at all taxonomic levels with samples 16S5 and 16S7 spanning regions V4–V6 and V1–V3, respectively, providing the best performances. This supports observations from Claesson et al. where the region spanning V4–V5 provided highly accurate results [19]. Conversely, gene regions spanning single variable regions displayed lower accuracy. For instance, although high read numbers were generated for sample 16S6 spanning V6, few unique sequences were generated. Consequently, only half of the bacteria present in the reference sample were correctly identified at the genus level using the SILVA database, despite a very low read misclassification level, indicating not enough variability is present in this region for accurate species identification. Spanning multiple variable regions is, however, also no guarantee, as evidenced by samples 16S10 and 16S11 that both span V8–V9, but for which nearly no reads were generated resulting in a very low percentage of correctly classified reads at all taxonomic levels. The low generated read numbers could potentially be due to suboptimal fragment amplification caused by problems in primer design and annealing, indicating amplification efficiency should also be considered for 16S rRNA gene region selection. Relative composition information of the reference sample was also available, allowing us to evaluate detected relative abundances. Since 16S rRNA gene copy numbers can vary between bacteria, the actual relative 16S rRNA gene abundance can, therefore, differ considerably from its total genomic composition [62]. We found, however, that, despite some exceptions (e.g., samples 16S2 and 16S3), predicted relative abundances differed strongly from their actual values without clear trends.

One particular bacterium consistently identified in the majority of samples at all taxonomic levels, *N. meningitidis*, was not part of the reference sample, but was processed during the same sequencing run as a separate mixed sample. This indicates bleed-through between different samples of the same sequencing run, despite using different barcodes for different samples. This phenomenon has been reported previously [63,64], and was dominant enough to result in a false positive identification for the majority of sequenced regions, directly implying that identification should account for the presence of dominant species sequenced in other samples, especially for orientation of unknown samples. Nevertheless, the maximum fraction of *N. meningitidis* reads in any sample was limited to 0.06%, suggesting that when multiple samples are sequenced in the same run, adjusting read thresholds for OTU detection to higher levels (e.g., 0.1%) can help with reducing false positive identifications. Additionally, several Illumina sequencing reagents contaminants have been described previously [65,66,67], some of which were also identified here in NTCs, such as *Delftia*, *Bradyrhizobium*, *Sphingomonas*, *Actinomyces*, *Corynebacterium*, *Devosia*, *Enhydrobacter*, *Mesorhizobium*, *Methylobacterium*, *Micrococcus*, *Stenotrophomonas*, *Streptococcus*, *Staphylococcus*, and *Pseudomonas* (an extended description is available in the Supplementary Information). These genera are typically filtered out by the bioinformatics workflow in actual samples during pre-processing due to their very low abundances, and did not interfere with identification, but could, nevertheless, present issues if they belong to genera such as *Pseudomonas* and *Staphylococcus* that also contain pathogenic bacteria of interest present in the reference sample (i.e., *P. aeruginosa* and *S. aureus*). Because detected read numbers for *P. aeruginosa* and *S. aureus* in the samples were always very high and their presence was expected, they could be excluded as contaminants. However, if either *P. aeruginosa* or *S. aureus* would be present in actual samples at low concentrations, this could be problematic through difficulties in differentiating between the actual presence of a highly pathogenic bacteria and a known contaminant, demonstrating that accurate species detection at very low abundances is problematic and that any pathogen identification supported by low read numbers merits scrutiny and additional follow-up investigation to provide confirmation.

An effect of the employed reference database on bacterial identification was observed with varying degrees for different regions, but with the NCBI 16S database generally leading to slightly better results. For instance, *S. enterica*, was properly identified at the genus level in two and six samples using the SILVA and NCBI 16S databases, respectively, whereas the genus *Azomonas* was incorrectly identified in four samples using the SILVA database, potentially explained by its high sequence similarity to the genus *Pseudomonas* [68,69], but never when using the NCBI 16S database. This effect can, however, most likely be explained by database size rather than quality. The SILVA database is substantially larger than the NCBI 16S database and contains more closely related sequences, consequently decreasing bootstrap support during classification. Conversely, the NCBI 16S database contains far fewer sequences so that during bootstrapping the chance of picking other bacteria is lower, resulting in higher support values.

Our results demonstrate that many confounding variables interplay during classification, such as intrinsic variability and length of selected 16S rRNA gene regions, amplification efficiency, dominant species present in other samples of the same sequencing run, potential Illumina sequencing workflow contaminants, and the reference database employed, rendering recommendation of only one particular 16S rRNA gene region difficult. A majority vote consensus approach was, therefore, considered aiming to leverage the biases of individual gene regions by considering classifications over all regions combined, while accounting for known contaminants and/or dominant species potentially, making the classification more robust compared to the separate regions (see Table 3 and Table 4). At the family level, this allowed perfect identification with both reference databases, with only sample 16S6 not identifying the *Enterococcaceae* but falsely identifying the *Carnobacteriaceae* when using the SILVA database. At the genus level, identification was perfect for the NCBI 16S database, with the exception of samples 16S1 and 16S6 not identifying *Salmonella*. For the SILVA database, because of the aforementioned database effect, employing a majority vote did not always result in correct identification, but this was mostly limited to closely related genera known to be problematic to differentiate even with traditional methods, such as *Salmonella* and *Escherichia* [70], and *Pseudomonas* and *Azomonas* [69]. This issue could potentially be mitigated by giving more weight to individual regions that provide better results, such as those spanning multiple variable regions (e.g., samples 16S5 and 16S7, spanning regions V4–V6 and V1–V3 respectively), but we could not resolve a cut-off for the presence/absence of bacteria because this would require extensive validation to derive thresholds broadly applicable, also for other species not considered here. It should be emphasized that simply employing the NCBI 16S database is not an optimal solution because, although it contains 16S sequence information for the majority of human and food pathogens, many other pathogens are not represented yet, and would, therefore, be impossible to detect. Moreover, as the NCBI database continues to grow, this database size effect will also start manifesting itself. Lastly, at the species level, identification becomes unreliable with in particular many false positive species identifications popping up.

### 3.2. Employing Long-Read Third-Generation (ONT) Sequencing to Characterize the Entire 16S rRNA Gene

The ONT technology generates long reads allowing to sequence the entire 16S rRNA gene, thereby providing an interesting alternative mitigating some of the aforementioned issues when using 16S rRNA gene regions, but it is unclear how higher read error rates affect performance. We, therefore, amplified the entire 16S rRNA gene in the reference sample, and performed sequencing with the ONT MinION. Because ONT technology represents a relatively recent innovation for which community standards and practices are still largely absent [34], three different workflows were investigated. Firstly, Mothur as a community standard for analysis of short-read data that also supports long-read data. Secondly, EPI2ME as a solution provided by ONT. Thirdly, GraphMap as a tool specifically designed for handling long error-prone reads. Both the SILVA and NCBI 16S reference databases were used, except for EPI2ME that only supports the NCBI 16S database. Our results generally indicate high accuracy at the taxonomic levels of the family and genus, but not the species. Mothur and EPI2ME respectively misclassified less than 0.02% and 0.93% of reads at the genus level, and 0% and 0.34% at the family level, for both reference databases. GraphMap showed large variation between the reference databases, misclassifying up to 2.09% and 18.48% of reads at the genus level using the NCBI and SILVA database, respectively. Using Mothur, only the genus *Enterobacter* was falsely identified supported by a single read. This is a known contaminant found in laboratory reagents [66], suggesting the potential existence of ONT workflow contaminants that currently are not well-documented due to the novelty of the technology. No bleed-through was observed, as for the MiSeq run, although its possibility cannot be excluded. At the species level, accuracy for all bioinformatics workflows decreased drastically with approximately 30%–40% of misclassified reads in each workflow. For instance, the species *L. monocytogenes* was not identified by Mothur and in only 2% of all reads classified as belonging to the genus *Listeria* by EPI2ME. This can most likely be explained by *Listeria* species exhibiting extremely high sequence similarity [71], far beyond the MinION read error rate. Similarly, *E. coli* was only identified by GraphMap, but several other species of the genera *Escherichia* and *Shigella* were incorrectly identified, most likely similarly attributable to high 16S rRNA gene sequence identity of species within and between these genera [72]. Although the majority of other species of the reference sample were correctly identified by most bioinformatic workflows, the high read error rates effectively entail that reliable species identification is infeasible due to the overwhelming number of false positives. Moreover, for the bacterial part of the reference sample, a trend for bacteria with low and high GC contents to be over and under-represented was observed, respectively, in addition to under-representation of the genus *Pseudomonas* due to primer mismatches.

Although the three different bioinformatics workflows display similar classification trends at different taxonomic levels, some workflow-specific characteristics, including the employed reference database, influence identification. For Mothur, although constituting a community standard with a SOP for MiSeq data, no such information is available yet for ONT data analysis, implying some employed settings were suboptimal. For instance, the “kmer-based bootstrapping” approach employed for MiSeq data is more robust because it accounts better for sequence similarity between closely related bacteria [73]. This method leads to high of erroneous kmers being queried due to the high ONT error rate. We therefore employed a “nearest neighbor” approach, wherein the single most similar database sequence is used for read classification. This method has been frequently used successfully [74,75,76], but does not account for inter-genus and inter-species variability. Another example is rare OTU filtering, which is advised as being set to removing singleton OTUs and OTUs containing less than 0.005% of reads for short-read data [77]. Since all ONT reads are singletons due to their high error rates, this setting had to be disabled, as otherwise, all sequenced reads were removed. For EPI2ME, lacking traceability concerning the algorithmic implementation and employed parameters and settings is a major limitation, since detailed information on this proprietary ONT workflow remains largely absent representing a “black box.” Moreover, although EPI2ME was freely available during our study, it is now hidden behind a paywall requiring users to buy credits to run the 16S workflow. For GraphMap, although no discernable reference database effects were observed for Mothur, nor could be evaluated for EPI2ME as it only spuports the NCBI 16S database, a major database effect on classification performance was observed because the GraphMap algorithm performs direct read mapping so that database size directly impact scoring values, resulting in more misclassifications when using the larger SILVA database. Since many settings can still be optimized for this approach, further research is required to determine how read mappers can account for error-prone longer reads against databases of different sizes, especially because SILVA contains sequences not present in the NCBI 16S database (see above). These observations illustrate the need for further community efforts to derive best practices for analyzing ONT data and render it difficult to confidently recommend one particular workflow, although in our set-up Mothur did distinctly outperform both EPI2ME and GraphMap.

Bioinformatics workflow considerations notwithstanding, classification performance at family and genus levels was remarkably high compared to MiSeq data, with all expected pathogens detected and nearly no false positive identifications, even when using a majority vote consensus approach for the MiSeq data. Our analysis demonstrates that despite the relatively high read error rates, the ability to capture the 16S rRNA gene sequence in its entire length improved classification accuracy considerably at the genus and family levels compared to Illumina sequencing of short 16S rRNA gene regions. Conversely, performance dropped substantially at the species level compared to MiSeq data due to the inclusion of a multitude of false positive species identifications. Although expected to some extent due to the high read error rates of ≈8%–15% [32,33], the performance drop between the genus and species level was particularly profound and suggests that using ONT data specifically for bacterial species identification is highly error-prone, especially for low-abundant species. This is in contrast to reports in literature for PacBio long-read sequencing for 16S-targeted genomics of the same reference material where accurate identification to the subspecies was reported made possible by the ability of this technology to sequence the same fragment several times so that the systematic read sequencing error rate could be reduced [57]. However, the ONT technology offers many advantages, such as its portability, possibility to perform real-time analysis, and its cost-effectiveness, as its price versus sequenced yield and buy-in price are much more attractive compared to PacBio, for which total costs remain out of reach for typical microbiology laboratories [29,78]. Moreover, as the ONT technology is still rapidly evolving through improvements in both sequencing chemistry and bioinformatics workflows, the error rate of this platform is still decreasing [79], rendering it an especially interesting avenue for future investigations and implementation for 16S-targeted genomics.

## 4. Conclusions

NGS technologies present an interesting alternative to traditional bacterial isolation and 16S rRNA gene amplification and Sanger sequencing for bacterial identification, especially for complex mixed samples, because they allow rapid orientation of samples by not requiring any intermediate culturing step(s). We performed a comparative evaluation of the performance of both second and third generation NGS technologies for bacterial identification based on 16S rRNA gene targeting and sequencing of a well-characterized reference sample, and found that both technologies offer their own advantages and disadvantages. In line with previous studies [23,59,80], the Illumina technology allowed for highly accurate characterization of 16S rRNA gene sequences, but is limited by short read lengths, allowing one to only span short gene regions. We evaluated different 16S rRNA gene regions employed in literature, and found pronounced differences in their abilities for reliable bacterial identification. Selecting regions spanning multiple 16S rRNA gene variable regions and combining information collected over different regions, substantially improved accuracy. In our analysis, the sequences spanning variable regions V4–V6 and V1–V3 yielded the best results, although other studies have recommended other regions [17]. Other confounding factors included amplification efficiency, dominant species present in other samples of the same sequencing run, potential contaminants of the Illumina sequencing workflow, and reference database employed. The ONT technology allowed sequencing the entire 16S rRNA gene region in line with other recent case studies [30,81,82], but was limited by higher read error rates complicating accurate read classification. Additionally, it represents a relatively novel technology for which community standards are still largely absent complicating the standardization and reproducibility of results. Despite these limitations, classification at the family and genus levels was remarkably accurate, especially after thorough pre-processing with Mothur, demonstrating that leveraging the entire 16S rRNA gene sequence is a powerful approach for bacterial identification in complex samples. Both sequencing technologies provided reliable classification down to the genus level and are, therefore, powerful methods for facilitating and accelerating sample orientation without any culturing steps, saving valuable time during crisis and outbreak situations. At the species level, limitations imposed by both sequencing technologies interplay with the limited discriminatory power of the 16S rRNA gene for certain genera, especially when present at low abundances, so that scrutiny is warranted requiring confirmation through additional tests in the laboratory to confidently confirm the presence or absence of bacterial species.

## 5. Materials and Methods

### 5.1. Reference Material

The ZymoBIOMICSTM Microbial Community DNA Standard (https://www.zymoresearch.com/zymobiomics-community-standard, D6305) was employed as reference sample. This mock community contains genomic DNA of eight bacterial species and two fungi. The exact composition in terms of genomic DNA and bacterial 16S rRNA gene contents is certified by the vendor, and presented in Figure 1.

### 5.2. Generation and Analysis of Sequencing Data for Different 16S rRNA Gene Regions with the Illumina (MiSeq) Technology

#### 5.2.1. Amplification and Sequencing

In total, 11 libraries were created from the mock community input DNA, each amplifying a different variable region or spanning different regions, and are presented in Table 1. The overhang adapter sequences, as specified by Illumina, were appended to these primers for compatibility with Illumina index and sequencing adapters. PCR and sequencing was performed as described in the 16S metagenomics Sequencing Library preparation protocol of Illumina (part number 15044223 rev A.) on an Illumina MiSeq instrument with a 300-bp paired-end protocol (MiSeq v3 chemistry); 15% PhiX was used as internal control.

#### 5.2.2. Bioinformatics Workflow

All bioinformatics analyses were performed using Mothur v1.39.1 [37], an open-source bioinformatics package widely employed for 16S rRNA gene analysis by the microbial ecology community, supporting different sequencing technologies and having extensive documentation and online community support. All analyses were performed according to recommendations for MiSeq data (https://www.mothur.org/wiki/MiSeq_SOP). Firstly, all reads were trimmed with Trimmomatic 0.36 [83] with the settings “LEADING:10,” “MINLEN:40,” “SLIDINGWINDOW:4:20,” and “TRAILING:10.” The trimmed paired reads were combined into contigs with the Mothur functionality “make.contigs,” after which “screen.seqs” was used to remove all contigs > 600 bases and contigs with more than five ambiguity characters when 75% of the contigs had five or less ambiguity characters. In case 75% of contigs had more than five ambiguity characters, all contigs with more ambiguity characters than the top 10% contigs were removed. Contigs with homopolymers longer than 15 bases were also removed when the longest homopolymer was shorter than 15 bases in 75% of contigs. As with ambiguity characters, when the longest homopolymer in 75% of contigs was longer than 15 bases, all contigs with longer homopolymers than the top 10% of contigs were removed. Afterwards, all unique contigs were selected with “unique.seqs.”

Selected contigs were then aligned to a 16S reference database with “align.seqs.” Two databases were employed. Firstly, the SILVA database [43,44] was employed, representing a widely used high-quality curated database containing bacterial 16S rRNA gene sequences that is freely available for academic and non-profit usage and for which software-specific versions are also available. The SILVA v128 database was downloaded from the Mothur website (https://www.mothur.org/wiki/Silva_reference_files). Because SILVA provides taxonomic resolution down to the genus level, we also employed the NCBI 16S database for bacteria (BioProject id 3317) and archaea (BioProject id 33317) that similarly contains high-quality curated 16S rRNA bacterial sequences but with a taxonomic resolution down to the species level, with as tradeoff that fewer sequences are present: 18,902 bacterial and 949 archaeal sequences (accessed 27 November 2017) compared to 168,111 bacterial and 4,337 archaeal sequences in the SILVA v128 database. As Mothur requires an aligned database, the 16S NCBI database was aligned to the SILVA database v128 with Mothur’s align.seqs functionality to make it compatible.

To ensure all sequences were overlapping in the same region, the distributions of start and end coordinates were determined with “summary.seqs.” Subsequently, all sequences were removed with “screen.seqs” that did not start at or before the 2.5th percentile start coordinate or ended before the 97.5th percentile end coordinate, if start and end coordinates were identical for both percentiles. If start and end coordinates for the 2.5th and 97.5th percentiles were not identical, median start and end coordinates were used. Alignments containing uninformative columns with only gaps or the “.” character were removed using “filter.seqs.” From the resulting filtered fasta file, only unique sequences were retained using “unique.seqs,” and low-abundant contigs likely containing sequencing errors were removed with “pre.cluster” when they were within two mismatches of highly abundant contigs. Next, the chimeric sequences were identified and removed with “chimera.vsearch,” after which the resulting fasta file was used to calculate a distance matrix before clustering sequences into operational taxonomic units (OTUs) with “cluster.” OTUs consisting out of a single contig or less than 0.005% of total contigs were removed, as they likely represented sequencing errors. Finally, contigs and OTUs were classified with the default Mothur “classify.seqs” functionality using the Wang approach [73], and a bootstrap cutoff of 80. All eukaryotic classifications were removed because the mock community also contained DNA from two fungal strains and SILVA also contains eukaryotic sequences. As Miseq read lengths are limited to 2 × 300 bp, maximum three variable regions could be spanned (see Table 1), resulting in potential differences in species identification and quantitative information for every sample. A majority vote consensus-based approach was therefore adapted wherein for any identified species, genus, and family, the number of occurrences within the different regions were counted.

### 5.3. Generation and Analysis of Sequencing Data for the Complete 16S rRNA Gene with the ONT (MinION) Technology

#### 5.3.1. Amplification and Sequencing

The library was prepared from the mock community DNA following the ONT protocol “16S Rapid Amplicon barcoding for the MinION using SQK-RAB201” (RAB_9067_v1_revD_10Apr2017) with a barcodes expansion pack (NBD103). Primers used for amplification were 27F and 1492R, which amplify the 16S rRNA gene spanning the variable regions V1–V9 and which were provided in the Rapid 16S amplicon barcoding kit (SQK-RAB201). The resulting library was sequenced with the MinION Mk 1B MIN-101B using flowcell FC-106-R9.4 for 48 h.

#### 5.3.2. Bioinformatics Workflow

Albacore v2.0.2 was used for basecalling, resulting in 1,108,690 reads that were processed with Porechop v0.2.2 (https://github.com/rrwick/Porechop) to confirm barcode assignment resulting in 1,099,067 reads, which is a substantial number necessitating computational resources beyond the availability of our infrastructure and what is typically available for clinical microbiology laboratories. We therefore retained the 10,000 first reads for further analysis. In contrast to Illumina, Nanopore sequencing represents a relatively recent endeavor and community standards are still emerging [39]. We therefore employed three different bioinformatics workflows.

Firstly, Mothur was used as described previously for MiSeq data but with some changes to account for nanopore reads: (i) generated reads were directly provided as input without combining paired end reads into contigs; (ii) reads longer than 1700 bp (the maximum expected length of the entire gene) were removed; (iii) since default classification did not yield any results because of high read error rate resulting in too low bootstrap values (<80%) for identification at the genus or species level, the nearest database sequence was used for classification; (iv) singletons were not removed, since every OTU was a singleton. Classification was performed independently against the SILVA and NCBI 16S databases described previously.

Secondly, EPI2ME (https://epi2me.nanoporetech.com, rev 2.1.0) is an online platform provided by ONT hosting several workflows for analyzing MinION data, including a 16S classification workflow that we employed (v2.2.9). This represents a user-friendly solution offered by ONT specifically tailored towards nanopore sequencing data that can be accessed through a web-interface, but cannot be installed locally so that processing time is dependent upon server load with the underlying algorithms representing a “black box.” EPI2ME only supports classification based on the 16S NCBI database, and information about its performance through independent studies is still largely absent. A version of the 16S NCBI database was used containing 18,927 16S sequences according to the Nanopore website (https://nanoporetech.com/analyse/16s, accessed 5 February 2018).

Thirdly, GraphMap v0.5.2 [42] is a mapping tool that is specifically designed for long error-prone reads, rendering it theoretically ideally suited for analyzing MinION data against reference databases. Raw nanopore reads were, therefore, directly mapped against the SILVA and NCBI 16S databases described previously with default settings.

### 5.4. Data Availability and Supplementary Material

The datasets supporting the conclusions of this study have been deposited in the NCBI Sequence Read Archive under BioProject number PRJNA587452, and are included within this manuscript and its supplementary files. A supplementary manuscript containing supplementary information, supplementary figures and tables is available as “Winand_supplementaryMaterial.pdf.”

## Figures and Tables

**Table 1:** Overview of samples generated for MiSeq sequencing, their corresponding amplified regions, primer pairs employed for amplification, fragment lengths, and references.

Sample Name	Amplified Region	Primer Pair	Fragment Length	Reference
16S1	V4	515F/806R1	292	[46]
16S2	V3–V4	341F1/806R2	466	[46]
16S3	V3–V4	341F2/805R	400–500	[47]
16S4	V4–V5	515F/907R	393	[46,48]
16S5	V4–V6	515F/1061R	546	[49]
16S6	V6	926F/1061R	135	[49,50]
16S7	V1–V3	8F/516R	488 (614)	[51]
16S8	V3–V4	341B4F/806R2	400–500	[47]
16S9	V8	1243F/1459R	217	[52]
16S10	V8–V9	1522F/1189R1	370	[53]
16S11	V8–V9	1522F/1189R2	370	[53]

**Table 2:** Classification results for MiSeq data for the different gene regions and taxonomic levels using the SILVA and NCBI 16S databases. Read numbers are expressed as percentages.

Sample	Number of Raw Contigs	SILVA							NCBI									
		Classified Contigs	Family			Genus			Classified Contigs	Family			Genus			Species		
			CC	MC	UC *	CC	MC	UC *		CC	MC	UC *	CC	MC	UC *	CC	MC	UC *
16S1	442,223	387,306	99.71%	0.03%	0.27%	48.18%	0.03%	51.79%	384,501	99.84%	0.03%	0.13%	80.92%	0.03%	19.05%	41.41%	8.09%	50.50%
16S2	370,429	307,971	99.97%	0.02%	0.02%	88.16%	0.02%	11.81%	308,254	99.98%	0.02%	0.00%	99.91%	0.02%	0.07%	57.57%	23.29%	19.14%
16S3	480,933	351,488	99.82%	0.03%	0.16%	87.05%	0.08%	12.87%	352,246	99.90%	0.03%	0.07%	99.52%	0.03%	0.45%	59.70%	22.89%	17.41%
16S4	336,363	193,320	99.61%	0.06%	0.33%	71.24%	0.09%	28.67%	206,531	99.70%	0.05%	0.24%	90.40%	0.05%	9.55%	52.19%	13.34%	34.48%
16S5	377,697	114,743	99.77%	0.00%	0.23%	99.74%	0.00%	0.26%	114,777	99.88%	0.00%	0.12%	93.01%	0.00%	6.99%	48.56%	14.02%	37.42%
16S6	550,931	506,640	68.91%	0.20%	30.89%	66.94%	0.01%	33.05%	522,128	58.16%	0.01%	41.83%	57.34%	0.01%	42.66%	33.32%	0.01%	66.68%
16S7	455,347	268,527	99.98%	0.02%	0.00%	99.98%	0.02%	0.00%	242,467	99.98%	0.02%	0.00%	99.98%	0.02%	0.00%	54.54%	27.47%	17.99%
16S8	557,407	440,145	99.94%	0.01%	0.05%	80.17%	0.04%	19.79%	440,811	99.98%	0.01%	0.01%	99.93%	0.01%	0.05%	58.32%	23.11%	18.57%
16S9	259	103	49.51%	50.49%	0.00%	29.13%	0.00%	70.87%	50	100.00%	0.00%	0.00%	64.00%	0.00%	36.00%	16.00%	24.00%	60.00%
16S10	158	0	-	-	-	-	-	-	0	-	-	-	-	-	-	-	-	-
16S11	210	16	0.00%	0.00%	100.00%	0.00%	0.00%	100.00%	9	0.00%	0.00%	100.00%	0.00%	0.00%	100.00%	0.00%	0.00%	100.00%

Abbreviations: CC: correctly classified; MC: misclassified; UC: unclassified; * Unclassified reads are reads that are not classified at the given level but at any higher level.

**Table 3:** Overview of bacteria identified in each gene region for MiSeq data using the SILVA database at different taxonomic levels. The first column lists the identification at the taxonomic level considered. The second column lists the total number of gene regions where the bacterium was identified. The next columns list the different samples (0 = not detected/1 = detected). Taxonomic names are colored per taxonomic level by a green gradient for bacteria present in the mock community (darker = correctly identified in more samples), and an orange gradient for bacteria not present in the mock community (darker = incorrectly identified in more samples).

	Total	16S1	16S2	16S3	16S4	16S5	16S6	16S7	16S8
**Family**									
*Pseudomonadaceae* †	**8**	1	1	1	1	1	1	1	1
*Enterobacteriaceae*	**8**	1	1	1	1	1	1	1	1
*Lactobacillaceae*	**8**	1	1	1	1	1	1	1	1
*Enterococcaceae*	**7**	1	1	1	1	1	0	1	1
*Staphylococcaceae* †	**8**	1	1	1	1	1	1	1	1
*Listeriaceae*	**8**	1	1	1	1	1	1	1	1
*Bacillaceae*	**8**	1	1	1	1	1	1	1	1
*Neisseriaceae* *	**7**	1	1	1	1	0	1	1	1
*Carnobacteriaceae*	**1**	0	0	0	0	0	1	0	0
**Genus**									
*Pseudomonadas* †	**4**	0	1	1	0	1	0	1	0
*Escherichia-Shigella*	**7**	1	1	1	1	1	0	1	1
*Salmonella*	**2**	0	0	0	0	1	0	1	0
*Lactobacillus*	**8**	1	1	1	1	1	1	1	1
*Enterococcus*	**5**	0	1	1	0	1	0	1	1
*Staphylococcus* †	**8**	1	1	1	1	1	1	1	1
*Listeria*	**8**	1	1	1	1	1	1	1	1
*Bacillus*	**7**	0	1	1	1	1	1	1	1
*Trabulsiella*	**3**	0	1	1	0	0	0	0	1
*Melissococcus*	**1**	0	0	0	1	0	0	0	0
*Azomonas*	**4**	1	0	1	1	0	0	0	1
*Neisseria* *	**7**	1	1	1	1	0	1	1	1
*Falsibacillus*	**1**	0	0	1	0	0	0	0	0

* Bleed-through originating from sequencing another mixed sample on the same sequencing run containing almost exclusively *Neisseria meningitidis*. † Known as potential Illumina contaminants.

**Table 4:** Overview of bacteria identified in each gene region for MiSeq data using the NCBI 16S database at different taxonomic levels. The first column lists the taxonomic name at the taxonomic level considered. The second column lists the total number of gene regions where the bacterium was identified. The next columns list the different samples (0 = not detected/1 = detected). Taxonomic names are colored per taxonomic level by a green gradient for bacteria present in the mock community (darker = correctly identified in more samples), and an orange gradient for bacteria not present in the mock community (darker = incorrectly identified in more samples).

	Total	16S1	16S2	16S3	16S4	16S5	16S6	16S7	16S8
**Family**									
*Pseudomonadaceae* †	**8**	1	1	1	1	1	1	1	1
*Enterobacteriaceae*	**8**	1	1	1	1	1	1	1	1
*Lactobacillaceae*	**8**	1	1	1	1	1	1	1	1
*Enterococcaceae*	**8**	1	1	1	1	1	1	1	1
*Staphylococcaceae* †	**8**	1	1	1	1	1	1	1	1
*Listeriaceae*	**8**	1	1	1	1	1	1	1	1
*Bacillaceae*	**8**	1	1	1	1	1	1	1	1
*Neisseriaceae* *	**7**	1	1	1	1	0	1	1	1
**Genus**									
*Pseudomonadas* †	**8**	1	1	1	1	1	1	1	1
*Escherichia-Shigella*	**8**	1	1	1	1	1	1	1	1
*Salmonella*	**6**	0	1	1	1	1	0	1	1
*Lactobacillus*	**8**	1	1	1	1	1	1	1	1
*Enterococcus*	**8**	1	1	1	1	1	1	1	1
*Staphylococcus* †	**8**	1	1	1	1	1	1	1	1
*Listeria*	**8**	1	1	1	1	1	1	1	1
*Bacillus*	**8**	1	1	1	1	1	1	1	1
*Neisseria* *	**7**	1	1	1	1	0	1	1	1
**Species**									
*Pseudomonadas aeruginosa*	**7**	1	1	1	1	1	0	1	1
*Escherichia coli*	**1**	0	0	0	0	0	1	0	0
*Salmonella enterica*	**6**	0	1	1	1	1	0	1	1
*Lactobacillus fermentum*	**8**	1	1	1	1	1	1	1	1
*Enterococcus faecalis*	**4**	0	1	1	0	0	0	1	1
*Staphylococcus aureus*	**8**	1	1	1	1	1	1	1	1
*Listeria monocytogenes*	**0**	0	0	0	0	0	0	0	0
*Bacillus subtilis*	**0**	0	0	0	0	0	0	0	0
*Neisseria polysaccharea* *	**4**	0	1	1	1	0	0	0	1
*Neisseria weaveri* *	**1**	0	0	0	0	0	1	0	0
*Enterococcus hirae*	**1**	1	0	0	0	0	0	0	0
*Enterococcus saccharolyticus*	**1**	0	0	0	0	1	0	0	0
*Listeria welshimeri*	**5**	0	1	1	1	1	0	0	1
*Escherichia fergusonii*	**6**	0	1	1	1	1	0	1	1
*Bacillus halotolerans*	**1**	0	0	0	0	0	0	1	0

* Bleed-through originating from sequencing another mixed sample on the same sequencing run containing almost exclusively *Neisseria meningitidis*. † Known as potential Illumina contaminants.

**Table 5:** Classification results for MinION data for the different bioinformatics workflows and taxonomic levels using the SILVA and NCBI 16S databases. The total number of analyzed reads was 10,000 for all three bioinformatics workflows while the number of reads each bioinformatics workflow could identify at any taxonomic level is indicated. The values of correctly classified, misclassified, and unclassified reads at a given level sum to the total number of classified reads at any level.

Workflow	SILVA						NCBI 16S								
	Family			Genus			Family			Genus			Species		
	CC	MC	UC *	CC	MC	UC *	CC	MC	UC *	CC	MC	UC *	CC	MC	UC *
Mothur	4799	0	0	4799	0	0	4429	0	0	4428	1	0	2593	1836	0
EPI2ME	-	-	-	-	-	-	8673	30	1135	7265	68	2505	4436	2897	2505
GraphMap	6949	145	0	5783	1311	0	7777	35	0	7649	163	0	4739	3073	0

Abbreviations: CC: correctly classified; MC: misclassified; UC: unclassified. * Unclassified reads are reads that are not classified at the given level but at any higher level.

## Supplementary Materials

The following are available online at https://www.mdpi.com/1422-0067/21/1/298/s1.

## Abbreviations

The following abbreviations are used in this manuscript:

HTS	high-throughput sequencing
NGS	next-generation sequencing
NTC	non-template control
ONT	Oxford Nanopore Technologies
SOP	standard operating procedure
