# Supplementary material for: Targeting the 16S rRNA Gene for Bacterial Identification in Complex Mixed Samples: Comparative Evaluation of Second (Illumina) and Third (Oxford Nanopore Technologies) Generation Sequencing Technologies
Source: Int J Mol Sci. 2019 Dec 31;21(1):298. doi: 10.3390/ijms21010298 (PMC6982111; doi:10.3390/ijms21010298)
Supplement: Supplementary file 1 [file ijms-21-00298-s001.zip › ijms-659682-proofreading si/ijms-659682-proofreading si.pdf]

Supplementary Materials: TARGETING THE 16S RRNA GENE FOR BACTERIAL IDENTIFICATION IN COMPLEX MIXED SAMPLES: COMPARATIVE EVALUATION OF SECOND (ILLUMINA) AND THIRD (OXFORD NANOPORE TECHNOLOGIES) GENERATION SEQUENCING TECHNOLOGIES

Raf Winand[1], Bert Bogaerts[1], Stefan Hoffman[1], Loïc Lefevre[1], Maud Delvoye [1], Julien Van Braekel[1], Qiang Fu[1], Nancy HC Roosens[1], Sigrid CJ De Keersmaecker[1,†] and Kevin Vanneste[1,†]

1. Description of Illumina MiSeq Raw and Processed Read Statistics for All Sequenced 16S rRNA Gene Regions

The total number of raw reads and raw contigs that were generated can be found in Table S1, as well as the number of preprocessed and unique contigs when using either the SILVA and NCBI 16S databases. Nearly no reads were generated for samples 16S9, 16S10, and 16S11, whereas sample 16S6 generated 573,391 raw reads and 550,391 contigs, with other samples varying between these two extremes. Of particular interest are the numbers of unique contigs obtained when using either database, which also varied widely between samples. For instance, sample 16S7 generated 272,475 preprocessed and 31,662 unique contigs using the SILVA database, and 245,850 preprocessed and 29,179 unique contigs using the NCBI 16S database. Closer inspection revealed that the number of ambiguity characters was very high, influencing the alignment and subsequent filtering steps. In contrast, sample 16S6 generated 509,679 preprocessed and 3215 unique contigs using the SILVA database, and 525,024 preprocessed and 3165 unique contigs using the NCBI 16S database, suggesting that not all diversity of the sample was captured by the reads, because the ratio between preprocessed and unique contigs is very large compared to the other samples. The number of reads classified per sample at each taxonomic level is presented in Table S2. The number of reads classified at the family level corresponds roughly with the number of pre-processed contigs for each sample indicating that the majority of pre-processed reads could be classified at the family level. The only exception is the aforementioned sample 16S6, where only 350,136 out of 509,679 reads, and 303,720 out of 525,024 reads, could be identified at the family level using the SILVA and NCBI 16S databases, respectively, which can be explained by the relatively low number of unique reads in this sample. Sample 16S6 was, however, a clear outlier with 30.89% and 41.83% of unclassified reads using the SILVA and NCBI 16S databases, respectively, which can most likely be explained by the relatively low number of unique reads compared to total reads resulting in large fluctuations when even a few unique reads are misclassified.

2. Description of Bacterial Species Detected in the Mock Community for All Sequenced 16S rRNA Gene Regions for the Illumina MiSeq Data

Below, a concise summary is provided for every bacterial species present in the mock community. Note that species identification was always only possible using the NCBI 16S database. For *B. subtilis*, the family *Bacillaceae* was identified in all samples using both the SILVA and NCBI 16S databases. The genus *Bacillus* was identified in all samples using the NCBI and SILVA databases, except for sample 16S1 using the SILVA database. There were no samples in which *B. subtilis* was identified at the species level. For *E. coli*, the family *Enterobacteriaceae* was identified in all samples using both the SILVA and NCBI 16S databases. The genus *Escherichia-Shigella* was identified in all samples except 16S9 using the NCBI 16S database, and in all samples except 16S9 and 16S6 using the SILVA database. Interestingly enough, the species *E. coli* was only identified in sample 16S6 despite its lower sequence quality. For *Enterococcus faecalis*, the family *Enterococcaceae* was identified in all samples using both the NCBI and SILVA databases, except for 16S6 using the SILVA database. The genus *Enterococcus* was identified in all

samples using the NCBI 16S database but not in samples 16S1, 16S4, and 16S6 using the SILVA database. The species *E. faecalis* was identified in samples 16S2, 16S3, 16S7, 16S8, and 16S9. For *Salmonella enterica*, the family *Enterobacteriaceae* was identified in all samples using both the SILVA and NCBI 16S databases, but classification at lower taxonomic levels was more problematic. The genus *Salmonella* was identified in only six samples (16S2, 16S3, 16S4, 16S5, 16S7, and 16S8) using the NCBI 16S database, and two samples (16S5 and 16S7) using the SILVA database. The species *S. enterica* was identified in the same six aforementioned samples. For *Listeria monocytogenes*, both the family *Listeriaceae* and the genus *Listeria* were identified in all samples using the SILVA and NCBI 16S databases. The species *L. monocytogenes* was, however, only identified in sample 16S9. For *Pseudomonas aeruginosa*, the family *Pseudomonadaceae* was identified in all samples using the SILVA and NCBI 16S databases. The genus *Pseudomonas* was identified in all samples using the NCBI 16S database, but only in four samples (16S2, 16S3, 16S5, and 16S7) using the SILVA database. The species *P. aeruginosa* was identified in all samples except 16S6 and 16S9. For *Lactobacillus fermentum*, both the family *Lactobacillaceae* and the genus *Lactobacillus* were identified in all samples using the SILVA and NCBI 16S databases. The species *L. fermentum* was identified in all samples except 16S9. For *S. aureus*, both the family *Staphylococcaceae* and the genus *Staphylococcus* were identified in all samples except 16S9 using the SILVA and NCBI 16S databases. The species *S. aureus* was similarly detected in all samples except 16S9.

3. Desription of Bacterial Species Detected in the Non-Template Controls for All Sequenced 16S rRNA Gene Regions for the Illumina MiSeq Data

All gene regions were accompanied by a NTC that was subjected to the same laboratory and bioinformatics workflow as the actual samples. The numbers of reads surviving preprocessing and resulting in classification were generally low in the NTC samples. We found that none of the false positives identified in the actual samples were present in the NTCs. Only bacteria known to be contaminants of the Illumina MiSeq sequencing workflow were detected in the NTCs, and were typically only supported by limited read numbers. At the family level, few reads were classified with sample 16S9, an outlier that had up to 2917 reads classified. At the genus level, for the organisms present in the mock community, only *Pseudomonas* and *Staphylococcus* were identified in between one (16S7) and three (16S1, 16S3, and 16S9) NTC samples, respectively, using both the SILVA and NCBI 16S databases. *Bacillus* was identified in two samples (16S4 and 16S9) only when using the NCBI 16S database. For organisms not present in the mock community, the large majority were only sporadically identified in one or two samples, with the exception of *Delftia* being identified in three samples (16S1, 16S3, and 16S4) using both the SILVA and NCBI 16S databases, and *Bradyrhizobium* and *Sphingomonas* being identified in five samples (16S1, 16S3, 16S4, 16S7, and 16S9) using the NCBI 16S database. Some of the other genera identified only sporadically in the NTC samples included *Actinomyces*, *Corynebacterium*, *Devosia*, *Enhydrobacter*, *Mesorhizobium*, *Methylobacterium*, *Micrococcus*, *Stenotrophomonas*, and *Streptococcus*. All these genera are, however, known contaminants of the Illumina sequencing process, as described previously [1–3]. A complete overview of all reads that were classified in the NTC samples can be found in Supplementary Tables 4–8. These contaminants were also found back in the actual samples, but were filtered out by the bioinformatics workflow during the preprocessing steps due to their extremely low abundance, and did not interfere with identification. Such contaminants could nevertheless present issues because some of them belong to genera such as *Pseudomonas* and *Staphylococcus*, which also contain pathogenic bacteria of interest present in the mock community (i.e., *P. aeruginosa* and *S. aureus*). Because the number of detected reads for the former bacteria in the actual samples was always very high, they could be excluded as contaminants. However, if either *P. aeruginosa* or *S. aureus* would be present in actual samples at low concentrations, this could be problematic through difficulties in differentiating between the actual presence of a highly pathogenic bacteria and a known contaminant.

4. Supplementary tables

Table S1. Overview of read and contigs statistics for MiSeq data. The first, second, third, and fourth columns list the sample name, number of raw reads for the sample, the exact community sequenced, and the number of contigs, respectively. The fifth and sixth columns list the number of preprocessed and unique contigs when the SILVA database was used. The seventh and eight columns list the numbers of preprocessed and unique contigs when the SILVA database was used.

Sample	Raw reads	Community	Contigs	SILVA		NCBI	
				Preprocessed contigs	Unique contigs	Preprocessed contigs	Unique contigs
16S1	550,363	ZymoBIOMICS	442,223	388,451	7,499	385,571	7,287
16S1	177,859	NTC	882	351	96	352	95
16S2	374,064	ZymoBIOMICS	370,429	309,038	13,446	309,281	13,227
16S2	186	NTC	20	4	4	8	5
16S3	485,522	ZymoBIOMICS	480,933	354,082	19,314	354,730	18,887
16S3	364	NTC	152	120	60	120	61
16S4	344,096	ZymoBIOMICS	336,363	194,088	7,980	207,346	8,171
16S4	779	NTC	210	174	55	174	55
16S5	390,381	ZymoBIOMICS	377,697	115,380	8,184	115,441	8,058
16S5	8,172	NTC	63	8	3	7	3
16S6	573,391	ZymoBIOMICS	550,931	509,679	3,215	525,024	3,165
16S6	842	NTC	43	10	5	10	5
16S7	461,396	ZymoBIOMICS	455,347	272,475	31,662	245,850	29,179
16S7	13,351	NTC	131	63	22	58	20
16S9	562,425	ZymoBIOMICS	557,407	443,250	26,506	443,732	25,814
16S9	193	NTC	30	5	4	7	6
16S10	314	ZymoBIOMICS	259	106	13	52	8
16S10	4,285	NTC	3,399	3,035	60	3,008	60
16S11	235	ZymoBIOMICS	158	0	0	0	0
16S11	20,841	NTC	866	3	3	8	3
16S12	278	ZymoBIOMICS	210	30	17	19	13
16S12	2,441	NTC	2,234	4	4	9	9

NTC: Non-template control

Table S2. Overview of number of reads classified in each sample at different taxonomic levels for the MiSeq data. The first column lists the sample name. The second, third, and fourth columns list the number of reads identified down to the family, genus, and species level, respectively, when using the NCBI 16S database. The fifth and sixth columns list the numbers of reads identified down to the family, and genus level, respectively, when using the SILVA database.

Sample	NCBI			SILVA	
	Family	Genus	Species	Family	Genus
16S1	383,986	311,248	190,338	386,270	186,732
16S2	308,254	308,038	249,264	307,920	271,592
16S3	352,002	350,652	290,912	350,938	306,269
16S4	206,029	186,816	135,323	192,676	137,890
16S5	114,640	106,755	71,825	114,479	114,448
16S6	303,720	299,408	173,996	350,136	339,190
16S7	242,467	242,467	198,856	268,527	268,527
16S9	440,754	440,577	358,968	439,940	353,022
16S10	50	32	20	103	30
16S11	0	0	0	0	0
16S12	0	0	0	0	0

Table S3. Overview of number of reads classified in sample 16S9 of the MiSeq data at all taxonomic levels. The first column lists the taxonomic level, the second column the taxon name, and the third and fourth column, the number of identified reads at that level using the SILVA and NCBI 16S databases, respectively.

Taxonomic level	Taxon	SILVA	NCBI 16S
Kingdom	*Bacteria*	103	50
Phylum	*Firmicutes*	30	29
Class	*Bacilli*	30	29
Order	*Bacillales*	17	17
Family	*Bacillaceae*	12	12
Genus	*Bacillus*	12	12
Species	*Bacillus halotolerans*	-	12
Family	*Listeriaceae*	5	5
Genus	*Listeria*	5	5
Species	*Listeria monocytogenes*	-	5
Order	*Lactobacillales*	13	12
Family	*Enterococcaceae*	3	3
Genus	*Enterococcus*	3	3
Species	*Enterococcus faecalis*	-	3
Family	*Lactobacillaceae*	10	9
Genus	*Lactobacillus*	10	9
Genus	*Lactobacillus_unclassified*	-	9
Phylum	*Proteobacteria*	73	21
Class	*Betaproteobacteria*	52	-
Order	*Neisseriales*	52	-
Family	*Neisseriaceae*	52	-
Genus	*Neisseriaceae_unclassified*	52	-
Class	*Gammaproteobacteria*	21	21
Order	*Enterobacteriales*	18	18
Family	*Enterobacteriaceae*	18	18
Family	*Enterobacteriaceae_unclassified*	18	18
Order	*Pseudomonadales*	3	3
Family	*Pseudomonadaceae*	3	3
Family	*Pseudomonadaceae_unclassified*	3	-
Genus	*Pseudomonas*	-	3
Genus	*Pseudomonas_unclassified*	-	3

Table S4. False positive genera and the percentage of reads assigned to them in each gene region for the MiSeq data when using the NCBI 16S and SILVA database.

NCBI 16S

	Sample										
**genus**	**16S1**	**16S2**	**16S3**	**16S4**	**16S5**	**16S6**	**16S7**	**16S8**	**16S9**	**16S10**	**16S11**
Neisseria	0.03	0.02	0.03	0.05	0.00	0.01	0.02	0.01	0.00	-	0.00
Azomonas	0.00	0.00	0.00	0.00	0.00	0.00	0.00	0.00	0.00	-	0.00
Trabulsiella	0.00	0.00	0.00	0.00	0.00	0.00	0.00	0.00	0.00	-	0.00
Melissococcus	0.00	0.00	0.00	0.00	0.00	0.00	0.00	0.00	0.00	-	0.00
Falsibacillus	0.00	0.00	0.00	0.00	0.00	0.00	0.00	0.00	0.00	-	0.00

SILVA

	Sample										
**genus**	**16S1**	**16S2**	**16S3**	**16S4**	**16S5**	**16S6**	**16S7**	**16S8**	**16S9**	**16S10**	**16S11**
Neisseria	0.03	0.02	0.03	0.06	0.00	0.01	0.02	0.01	50.49	-	0.00
Azomonas	0.01	0.00	0.02	0.02	0.00	0.00	0.00	0.01	0.00	-	0.00
Trabulsiella	0.00	0.00	0.03	0.00	0.00	0.00	0.00	0.02	0.00	-	0.00
Melissococcus	0.00	0.00	0.00	0.01	0.00	0.00	0.00	0.00	0.00	-	0.00
Falsibacillus	0.00	0.00	0.01	0.00	0.00	0.00	0.00	0.00	0.00	-	0.00

Table S5. Overview of number of reads that were assigned to each species using the NCBI 16S database when using EPI2ME, Mothur, and GraphMap for classification for the MinION data. For each species present in the mock community (indicated in the first column), all other species belonging to the same genus that were assigned at least one read are presented (indicated in the second column). The theoretical abundance of 16S rRNA belonging to species of the mock community is indicated in the third column. The next columns list the number of reads identified at the taxonomic level of the family, genus, and species for the three different bioinformatics workflows. The entries "OTHER HITS n=1" and "OTHER HITS n>1" refer to all reads identified as species for which a genus was not present in the mock community, including and excluding single hits, respectively.

			**EPI2ME**			**Mothur**			**GraphMap**		
**Species in sample**	**%sample**	**Identified species**	**Family**	**Genus**	**Species**	**Family**	**Genus**	**Species**	**Family**	**Genus**	**Species**
Pseudomonas aeruginosa	4.20%	TOTAL	192	192		85	85		190	190	
		Pseudomonas aeruginosa			179			85			172
		Pseudomonas alcaligenes			1			0			2
		Pseudomonas delhiensis			0			0			1
		Pseudomonas graminis			1			0			0
		Pseudomonas knackmussii			1			0			0
		Pseudomonas mendocina			0			0			1
		Pseudomonas nitroreducens			1			0			0
		Pseudomonas oleovorans			0			0			2
		Pseudomonas otitidis			5			0			11
		Pseudomonas pelagia			0			0			1
		Pseudomonas resinovorans			4			0			0
Escherichia coli	10.10%	TOTAL	1,607	36		1,117	432		2,057	728	
		Escherichia coli			0			0			12
		Escherichia albertii			0			0			1
		Escherichia fergusonii			11			33			324
		Escherichia marmotae			5			88			320
		Shigella boydii			0			0			9
		Shigella dysenteriae			2			103			18
		Shigella flexneri			13			128			16
		Shigella sonnei			5			80			28
Salmonella enterica	10.40%	TOTAL	1,607	289		1,117	684		2,057	1,209	
		Salmonella enterica			288			684			1,168
		Salmonella bongori			1			0			41
Lactobacillus fermentum	18.40%	TOTAL	934	929		193	193		969	968	
		Lactobacillus fermentum			773			193			560
		Lactobacillus alvi			1			0			0
		Lactobacillus collinoides			1			0			0
		Lactobacillus equigenerosi			6			0			4
		Lactobacillus gastricus			85			0			1

			EPI2ME			Mothur			GraphMap		
**Species in sample**	**%sample**	**Identified species**	**Family**	**Genus**	**Species**	**Family**	**Genus**	**Species**	**Family**	**Genus**	**Species**
		Lactobacillus gorillae			6			0			397
		Lactobacillus ingluviei			7			0			0
		Lactobacillus mixtipabuli			1			0			0
		Lactobacillus mucosae			15			0			3
		Lactobacillus nagelii			0			0			2
		Lactobacillus reuteri			31			0			0
		Lactobacillus siliginis			1			0			0
		Lactobacillus vaccinostercus			1			0			0
		Lactobacillus wasatchensis			1			0			1
Enterococcus faecalis	9.90%	TOTAL	886	870		565	565		1,141	1,141	
		Enterococcus faecalis			781			564			1,120
		Enterococcus asini			1			0			0
		Enterococcus avium			8			0			0
		Enterococcus bulliens			1			0			0
		Enterococcus canintestini			1			0			0
		Enterococcus canis			5			0			0
		Enterococcus dispar			8			0			0
		Enterococcus durans			1			0			0
		Enterococcus eurekensis			0			0			1
		Enterococcus faecium			4			0			5
		Enterococcus gallinarum			4			0			0
		Enterococcus haemoperoxidus			2			0			2
		Enterococcus hirae			17			0			0
		Enterococcus lemanii			0			0			1
		Enterococcus malodoratus			1			0			0
		Enterococcus moraviensis			4			0			4
		Enterococcus mundtii			1			0			0
		Enterococcus pseudoavium			4			0			1
		Enterococcus rivorum			3			0			0
		Enterococcus rotai			0			1			0
		Enterococcus saccharolyticus			11			0			1
		Enterococcus silesiacus			1			0			0
		Enterococcus sulfureus			10			0			6
		Enterococcus termitis			2			0			0
Staphylococcus aureus	15.50%	TOTAL	1,547	1,536		863	863		1,555	1,554	
		Staphylococcus aureus			1,416			860			1,412
		Staphylococcus capitis			3			0			0
		Staphylococcus caprae			20			0			5

Species in sample	%sample	Identified species	EPI2ME Family	EPI2ME Genus	EPI2ME Species	Mothur Family	Mothur Genus	Mothur Species	GraphMap Family	GraphMap Genus	GraphMap Species
		Staphylococcus carnosus			0			0			2
		Staphylococcus chromogenes			1			0			0
		Staphylococcus cohnii			2			0			1
		Staphylococcus delphini			0			0			1
		Staphylococcus devriesei			3			0			2
		Staphylococcus epidermidis			1			0			0
		Staphylococcus equorum			0			0			2
		Staphylococcus haemolyticus			4			0			15
		Staphylococcus hominis			27			0			5
		Staphylococcus kloosii			1			0			0
		Staphylococcus pasteuri			1			0			1
		Staphylococcus petrasii			10			0			36
		Staphylococcus pettenkoferi			1			0			0
		Staphylococcus piscifermentans			2			0			0
		Staphylococcus pseudintermedius			0			0			3
		Staphylococcus saccharolyticus			34			0			36
		Staphylococcus saprophyticus			5			0			0
		Staphylococcus sciuri			2			0			0
		Staphylococcus simiae			2			3			31
		Staphylococcus simulans			0			0			1
		Staphylococcus stepanovicii			0			0			1
		Staphylococcus xylosus			1			0			0
Listeria monocytogenes	14.10%	TOTAL	1,320	1,280		588	588		1,233	1,233	
		Listeria monocytogenes			26			0			13
		Listeria aquatica			0			0			1
		Listeria grayi			1			0			1
		Listeria innocua			205			1			771
		Listeria ivanovii			19			0			2
		Listeria marthii			3			0			3
		Listeria riparia			1			0			1
		Listeria rocourtiae			0			0			1
		Listeria seeligeri			97			100			149
		Listeria weihenstephanensis			2			0			7
		Listeria welshimeri			926			487			284
Bacillus subtilis	17.40%	TOTAL	2,187	2,133		1,018	1,018		632	626	
		Bacillus subtilis			973			207			282
		Bacillus acidicola			6			0			0
		Bacillus altitudinis			1			0			0

**Species in sample**	**%sample**	**Identified species**	**EPI2ME**			**Mothur**			**GraphMap**		
			**Family**	**Genus**	**Species**	**Family**	**Genus**	**Species**	**Family**	**Genus**	**Species**
		Bacillus amyloliquefaciens			88			0			2
		Bacillus aquimaris			4			0			0
		Bacillus asahii			1			0			0
		Bacillus atrophaeus			89			0			1
		Bacillus australimaris			2			0			0
		Bacillus berkeleyi			1			0			0
		Bacillus carboniphilus			1			0			0
		Bacillus cereus			1			0			0
		Bacillus cytotoxicus			1			0			0
		Bacillus depressus			1			0			0
		Bacillus fengqiuensis			1			0			1
		Bacillus firmus			8			0			0
		Bacillus flexus			2			0			0
		Bacillus galliciensis			2			0			0
		Bacillus ginsengihumi			1			0			0
		Bacillus gobiensis			1			0			0
		Bacillus gottheilii			5			0			0
		Bacillus halotolerans			357			675			166
		Bacillus hemicellulosilyticus			1			0			0
		Bacillus isabeliae			1			0			0
		Bacillus kribbensis			1			0			0
		Bacillus lentus			1			0			0
		Bacillus licheniformis			14			0			1
		Bacillus massiliosenegalensis			1			0			0
		Bacillus massiloanorexius			1			0			0
		Bacillus mediterraneensis			0			0			1
		Bacillus mesonae			0			0			1
		Bacillus mojavensis			397			131			24
		Bacillus nakamurai			0			1			91
		Bacillus nematocida			18			1			8
		Bacillus oleronius			7			0			0
		Bacillus pseudofirmus			2			0			0
		Bacillus seohaeanensis			1			0			0
		Bacillus shackletonii			1			0			0
		Bacillus siamensis			11			0			3
		Bacillus songklensis			2			0			0
		Bacillus sonorensis			1			0			0
		Bacillus sporothermodurans			2			0			0

Species in sample	%sample	Identified species	EPI2ME Family	EPI2ME Genus	EPI2ME Species	Mothur Family	Mothur Genus	Mothur Species	GraphMap Family	GraphMap Genus	GraphMap Species
		Bacillus stratosphericus			2			0			0
		Bacillus subterraneus			1			0			0
		Bacillus tequilensis			8			0			12
		Bacillus testis			1			0			0
		Bacillus toyonensis			1			0			0
		Bacillus vallismortis			108			3			19
		Bacillus velezensis			1			0			14
		Bacillus vietnamensis			1			0			0
		Bacillus wakoensis			1			0			0
		Bacillus xiamenensis			1			0			0
OTHER HITS n = 1	N/A	N/A	30	68	68	0	1	1	35	163	163
OTHER HITS n >1	N/A	N/A	28	62	50	0	0	0	20	133	121

Table S6. Overview of number of reads that were classified at the genus level using the NCBI and SILVA databases when using EPI2ME, Mothur, and GraphMap for the MinION data. Only genera that were not expected to be present are listed. The first column lists the genus names. The second, third, and fourth columns list the number of reads identified for each genus when using the NCBI 16S database and EPI2ME, Mothur, and GraphMap, respectively. The fifth and sixth columns list the number of reads identified for each genus when using the SILVA database and Mothur, and GraphMap, respectively.

	NCBI			SILVA	
**Genus**	**EPI2ME**	**Mothur**	**GraphMap**	**Mothur**	**GraphMap**
0319-6M6_ge	-	-	-	-	1
Alkalibacterium	1	-	-	-	-
Anoxybacillus	-	-	1	-	13
Arsukibacterium	-	-	1	-	-
Atlantibacter	-	-	1	-	-
Brevibacillus	-	-	-	-	3
Burkholderia-Paraburkholderia	-	-	-	-	4
Buttiauxella	-	-	3	-	-
Caldicellulosiruptor	-	-	-	-	3
Cardiobacterium	-	-	-	-	1
Cedecea	2	-	-	-	-
Chitinophaga	-	-	1	-	-
Citrobacter	18	-	21	-	81
Colwellia	-	-	-	-	1
Cronobacter	1	-	3	-	-
Enterobacter	18	1	49	-	455
Erwinia	-	-	2	-	-
Frischella	-	-	1	-	-
Idiomarina	-	-	1	-	-
Klebsiella	2	-	1	-	510
Kluyvera	-	-	-	-	1
Kosakonia	4	-	10	-	-
Leclercia	1	-	4	-	-
Lysinibacillus	-	-	1	-	1
Macrococcus	4	-	1	-	-
Mangrovibacter	-	-	1	-	-
Massilia	-	-	1	-	-
Moraxella	-	-	1	-	-
Mycoplasma	-	-	1	-	-
Oceanimonas	-	-	-	-	1
Oceanobacillus	3	-	1	-	-
Orenia	-	-	1	-	-
Ornithinibacillus	-	-	1	-	-
Pandoraea	-	-	1	-	-
Pantoea	2	-	4	-	6
Pectobacterium	-	-	1	-	11
Pediococcus	-	-	1	-	-
Phaeochromatium	-	-	1	-	-
Photobacterium	-	-	-	-	1

	NCBI			SILVA	
**Genus**	**EPI2ME**	**Mothur**	**GraphMap**	**Mothur**	**GraphMap**
Photorhabdus	-	-	1	-	-
Planococcus	-	-	-	-	1
Planomicrobium	-	-	-	-	2
Pluralibacter	-	-	19	-	-
Pontibacillus	-	-	-	-	54
Proteus	-	-	-	-	1
Pseudescherichia	-	-	5	-	-
Pseudoalteromonas	-	-	2	-	38
Raoultella	-	-	1	-	21
Rhodovulum	-	-	1	-	-
Rickettsiales mitochondria_ge	-	-	-	-	1
Ruminiclostridium	-	-	-	-	7
Salinococcus	1	-	-	-	-
Serratia	4	-	-	-	3
Shewanella	-	-	3	-	-
Sinobaca	-	-	1	-	-
Sporolactobacillus	-	-	-	-	1
Sporotomaculum	-	-	2	-	-
Streptococcus	-	-	-	-	33
Sva0996_marine_group_ge	-	-	-	-	1
Tatumella	1	-	-	-	-
Terrilactibacillus	-	-	1	-	-
Thauera	-	-	1	-	-
Thioprofundum	-	-	1	-	-
Thiothrix	-	-	-	-	7
Thorsellia	-	-	1	-	1
Trabulsiella	-	-	-	-	3
Ursidibacter	-	-	1	-	-
Vagococcus	-	-	-	-	2
Vibrio	5	-	2	-	37
Virgibacillus	-	-	1	-	1
Xenorhabdus	-	-	2	-	-
Yersinia	1	-	-	-	3
Yokenella	-	-	2	-	-
uncultured	-	-	-	-	1
**TOTAL**	**68**	**1**	**163**	**0**	**1,311**

Table S7. Overview of number of reads that were classified at the family level using the NCBI and SILVA databases when using EPI2ME, Mothur, and GraphMap for the MinION data. Only families that were not expected to be present are listed. The first column lists the family name. The second, third, and fourth columns list the number of reads identified for the family when using the NCBI 16S database and EPI2ME, Mothur, and GraphMap, respectively. The fifth and sixth columns list the number of reads identified for the genus when using the SILVA database and Mothur, and GraphMap, respectively.

	NCBI			SILVA	
**Family**	**EPI2ME**	**Mothur**	**GraphMap**	**Mothur**	**GraphMap**
0319-6M6	-	-	-	-	1
Aeromonadaceae	-	-	-	-	1
Budviciaceae	1	-	-	-	-
Burkholderiaceae	-	-	1	-	4
Cardiobacteriaceae	-	-	-	-	1
Carnobacteriaceae	8	-	-	-	-
Chitinophagaceae	-	-	1	-	-
Chromatiaceae	-	-	2	-	-
Colwelliaceae	-	-	-	-	1
Erwiniaceae	6	-	6	-	-
Family_III	-	-	-	-	3
Halobacteroidaceae	-	-	1	-	-
Idiomarinaceae	-	-	1	-	-
Mitochondria	-	-	-	-	1
Moraxellaceae	-	-	1	-	-
Morganellaceae	-	-	3	-	-
Mycoplasmataceae	-	-	1	-	-
Orbaceae	-	-	1	-	-
Oxalobacteraceae	-	-	1	-	-
Paenibacillaceae	-	-	-	-	3
Pasteurellaceae	-	-	1	-	-
Pectobacteriaceae	-	-	1	-	-
Peptococcaceae	-	-	2	-	-
Planococcaceae	1	-	-	-	4
Pseudoalteromonadaceae	-	-	2	-	38
Rhodobacteraceae	-	-	1	-	-
Ruminococcaceae	-	-	-	-	8
Shewanellaceae	-	-	3	-	-
Sporolactobacillaceae	-	-	1	-	1
Streptococcaceae	-	-	-	-	33
Sva0996_marine_group	-	-	-	-	1
Thioprofundaceae	-	-	1	-	-
Thiotrichaceae	-	-	-	-	7
Thorselliaceae	-	-	1	-	-
Vibrionaceae	8	-	2	-	38
Yersiniaceae	6	-	-	-	-
Zoogloeaceae	-	-	1	-	-
**TOTAL**	**30**	**0**	**35**	**0**	**145**

Table S8. Overview of number of reads classified in the negative-template control samples at the family level using the SILVA database for the MiSeq data. The first column lists the family name, and the next columns all list the negative-template control samples. Bold entries represent families that were part of the mock community.

**Family**	**Sample**										
	**16S1**	**16S2**	**16S3**	**16S4**	**16S5**	**16S6**	**16S7**	**16S8**	**16S9**	**16S10**	**16S11**
Pseudomonadaceae	6	-	12	-	-	-	-	-	15	-	-
Staphylococcaceae	-	-	-	-	-	-	2	-	-	-	-
Bacillaceae	-	-	-	7	-	-	-	-	1,614	-	-
Actinomycetaceae	-	-	-	-	-	-	-	-	13	-	-
Bradyrhizobiaceae	30	-	9	20	-	-	8	-	115	-	-
Brucellaceae	-	-	14	-	-	-	-	-	84	-	-
Caulobacteraceae	-	-	-	10	-	-	-	-	164	-	-
Chitinophagaceae	20	-	-	-	-	-	-	-	-	-	-
Comamonadaceae	70	-	17	36	-	-	7	-	-	-	-
Corynebacteriaceae	-	-	-	-	-	-	-	-	50	-	-
Deinococcaceae	-	-	-	-	-	-	-	-	34	-	-
Elev-16S-1332	-	-	-	-	-	-	-	-	30	-	-
Halomonadaceae	-	-	-	-	-	-	-	-	92	-	-
Hyphomicrobiaceae	-	-	-	-	-	-	-	-	13	-	-
Intrasporangiaceae	-	-	-	-	-	-	5	-	-	-	-
Methylobacteriaceae	-	-	-	-	-	-	-	-	5	-	-
Micrococcaceae	21	-	-	-	-	5	-	-	79	-	-
Mitochondria	-	-	11	-	-	-	-	-	-	-	-
Moraxellaceae	-	-	-	-	-	-	-	-	37	-	-
Neisseriaceae	2	2	-	-	-	-	-	2	-	-	-
Oxalobacteraceae	36	-	-	22	-	-	4	-	-	-	-
Pasteurellaceae	-	-	-	-	-	-	-	-	75	-	-
Phyllobacteriaceae	37	-	5	2	-	-	-	-	-	-	-
Planococcaceae	-	-	-	-	-	-	-	-	141	-	-
Propionibacteriaceae	3	-	-	-	-	-	19	-	-	-	-
Rhizobiaceae	27	-	-	-	-	-	-	-	-	-	-
Rhodocyclaceae	-	-	-	16	-	-	-	-	-	-	-
Sphingomonadaceae	25	-	-	-	-	-	4	-	-	-	-
Streptococcaceae	-	-	-	-	-	-	-	-	231	-	-
Xanthomonadaceae	-	-	7	-	-	-	2	-	-	-	-
env.OPS_17	69	-	8	25	-	-	3	-	125	-	-
**TOTAL**	**346**	**2**	**83**	**138**	**0**	**5**	**54**	**2**	**2,917**	**0**	**0**

Table S9. Overview of number of reads classified in the negative-template control samples at the family level using the NCBI 16S database the MiSeq data. The first column lists the family name, and the next columns all list the negative-template control samples. Bold entries represent families that were part of the mock community.

**Family**	**Sample**										
	**16S1**	**16S2**	**16S3**	**16S4**	**16S5**	**16S6**	**16S7**	**16S8**	**16S9**	**16S10**	**16S11**
Pseudomonadaceae	6	-	12	-	-	-	-	-	15	-	-
Staphylococcaceae	-	-	-	-	-	-	2	-	-	-	-
Bacillaceae	-	-	-	7	-	-	-	-	1,755	-	-
Actinomycetaceae	-	-	-	-	-	-	-	-	13	-	-
Bradyrhizobiaceae	30	-	9	20	-	-	8	-	78	-	-
Brucellaceae	-	-	14	-	-	-	-	-	-	-	-
Caulobacteraceae	2	-	-	10	-	-	-	-	164	-	-
Chitinophagaceae	20	-	-	-	-	-	-	-	-	-	-
Comamonadaceae	70	-	14	36	-	-	7	-	-	-	-
Corynebacteriaceae	-	-	-	-	-	-	-	-	22	-	-
Deinococcaceae	-	-	-	-	-	-	-	-	34	-	-
Gordoniaceae	-	-	-	-	-	-	-	-	37	-	-
Halomonadaceae	-	-	-	-	-	-	-	-	92	-	-
Hyphomicrobiaceae	-	-	-	-	-	-	-	-	13	-	-
Methylobacteriaceae	-	-	-	-	-	-	-	-	5	-	-
Micrococcaceae	22	-	-	-	-	5	-	-	79	-	-
Moraxellaceae	-	-	-	-	-	-	-	-	37	-	-
Neisseriaceae	2	-	-	-	-	-	-	-	-	-	-
Oxalobacteraceae	36	-	-	22	-	-	4	-	-	-	-
Pasteurellaceae	-	-	-	-	-	-	-	-	75	-	-
Phyllobacteriaceae	-	-	5	2	-	-	-	-	-	-	-
Propionibacteriaceae	3	-	-	-	-	-	19	-	-	-	-
Rhizobiaceae	27	-	-	-	-	-	-	-	34	-	-
Sphingomonadaceae	25	-	14	31	-	-	4	-	23	-	-
Streptococcaceae	-	-	-	-	-	-	-	-	232	-	-
Xanthomonadaceae	-	-	7	-	-	-	2	-	-	-	-
Zoogloeaceae	-	-	-	16	-	-	-	-	-	-	-
**TOTAL**	**243**	**0**	**75**	**144**	**0**	**5**	**46**	**0**	**2,708**	**0**	**0**

Table S10. Overview of number of reads classified in the negative-template control samples at the genus level using the SILVA database for the MiSeq data. The first column lists the genus name, and the next columns all list the negative-template control samples. Bold entries represent families that were part of the mock community.

**Genus**	**Sample**										
	**16S1**	**16S2**	**16S3**	**16S4**	**16S5**	**16S6**	**16S7**	**16S8**	**16S9**	**16S10**	**16S11**
Pseudomonas	6	-	12	-	-	-	-	-	-	-	-
Staphylococcus	-	-	-	-	-	-	2	-	-	-	-
Actinobacillus	-	-	-	-	-	-	-	-	75	-	-
Anaerobacillus	-	-	-	7	-	-	-	-	1,607	-	-
Bosea	15	-	-	-	-	-	-	-	37	-	-
Bradyrhizobium	-	-	9	-	-	-	-	-	78	-	-
Brevundimonas	-	-	-	-	-	-	-	-	24	-	-
Brucella	-	-	14	-	-	-	-	-	-	-	-
Deinococcus	-	-	-	-	-	-	-	-	34	-	-
Delftia	53	-	12	36	-	-	-	-	-	-	-
Devosia	-	-	-	-	-	-	-	-	13	-	-
Enhydrobacter	-	-	-	-	-	-	-	-	37	-	-
Herbaspirillum	-	-	-	22	-	-	-	-	-	-	-
Lacibacter	20	-	-	-	-	-	-	-	-	-	-
Lysinibacillus	-	-	-	-	-	-	-	-	141	-	-
Mesorhizobium	-	-	5	2	-	-	-	-	-	-	-
Methylobacterium	-	-	-	-	-	-	-	-	5	-	-
Micrococcus	21	-	-	-	-	-	-	-	-	-	-
Neisseria	2	-	-	-	-	-	-	-	-	-	-
Ornithinimicrobium	-	-	-	-	-	-	5	-	-	-	-
Pelomonas	-	-	-	-	-	-	7	-	-	-	-
Propionibacterium	3	-	-	-	-	-	19	-	-	-	-
Rhizobium	27	-	-	-	-	-	-	-	-	-	-
Sphingomonas	-	-	-	-	-	-	4	-	-	-	-
Stenotrophomonas	-	-	7	-	-	-	2	-	-	-	-
Streptococcus	-	-	-	-	-	-	-	-	231	-	-
Thauera	-	-	-	16	-	-	-	-	-	-	-
Undibacterium	-	-	-	-	-	-	4	-	-	-	-
uncultured	-	-	-	10	-	-	-	-	-	-	-
**TOTAL**	**147**	**0**	**59**	**93**	**0**	**0**	**43**	**0**	**2,282**	**0**	**0**

Table S11. Overview of number of reads classified in the negative-template control samples at the genus level using the NCBI 16S database for the MiSeq data. The first column lists the genus name, and the next columns all list the negative-template control samples. Bold entries represent families that were part of the mock community.

**Genus**	**Sample**										
	**16S1**	**16S2**	**16S3**	**16S4**	**16S5**	**16S6**	**16S7**	**16S8**	**16S9**	**16S10**	**16S11**
Pseudomonas	6	-	12	-	-	-	-	-	15	-	-
Staphylococcus	-	-	-	-	-	-	2	-	-	-	-
Bacillus	-	-	-	7	-	-	-	-	4	-	-
Actinomyces	-	-	-	-	-	-	-	-	13	-	-
Agrobacterium	-	-	-	-	-	-	-	-	34	-	-
Bosea	15	-	-	-	-	-	-	-	-	-	-
Bradyrhizobium	15	-	9	20	-	-	8	-	78	-	-
Brevundimonas	-	-	-	-	-	-	-	-	24	-	-
Caulobacter	-	-	-	-	-	-	-	-	140	-	-
Corynebacterium	-	-	-	-	-	-	-	-	22	-	-
Cutibacterium	3	-	-	-	-	-	19	-	-	-	-
Deinococcus	-	-	-	-	-	-	-	-	34	-	-
Delftia	53	-	12	36	-	-	-	-	-	-	-
Devosia	-	-	-	-	-	-	-	-	13	-	-
Gordonia	-	-	-	-	-	-	-	-	37	-	-
Halomonas	-	-	-	-	-	-	-	-	92	-	-
Herbaspirillum	36	-	-	22	-	-	-	-	-	-	-
Lysinibacillus	-	-	-	-	-	-	-	-	141	-	-
Mesorhizobium	-	-	5	2	-	-	-	-	-	-	-
Methylobacterium	-	-	-	-	-	-	-	-	5	-	-
Micrococcus	22	-	-	-	-	-	-	-	-	-	-
Moraxella	-	-	-	-	-	-	-	-	37	-	-
Neisseria	2	-	-	-	-	-	-	-	-	-	-
Nesterenkonia	-	-	-	-	-	5	-	-	-	-	-
Ochrobactrum	-	-	14	-	-	-	-	-	-	-	-
Pelomonas	-	-	-	-	-	-	7	-	-	-	-
Phenylobacterium	2	-	-	10	-	-	-	-	-	-	-
Rhizobium	27	-	-	-	-	-	-	-	-	-	-
Sphingomonas	25	-	14	31	-	-	4	-	23	-	-
Stenotrophomonas	-	-	7	-	-	-	2	-	-	-	-
Streptococcus	-	-	-	-	-	-	-	-	232	-	-
Thauera	-	-	-	16	-	-	-	-	-	-	-
Undibacterium	-	-	-	-	-	-	4	-	-	-	-
Variovorax	-	-	2	-	-	-	-	-	-	-	-
**TOTAL**	**206**	**0**	**75**	**144**	**0**	**5**	**46**	**0**	**944**	**0**	**0**

Table S12. Overview of number of reads classified in the negative-template control samples at the species level using the NCBI 16S database for the MiSeq data. The first column lists the species name, and the next columns all list the negative-template control samples. Bold entries represent families that were part of the mock community.

**Species**	**Sample**										
	**16S1**	**16S2**	**16S3**	**16S4**	**16S5**	**16S6**	**16S7**	**16S8**	**16S9**	**16S10**	**16S11**
Bradyrhizobium japonicum	-	-	-	-	-	-	-	-	78	-	-
Brevundimonas diminuta	-	-	-	-	-	-	-	-	24	-	-
Caulobacter henricii	-	-	-	-	-	-	-	-	140	-	-
Cutibacterium acnes	3	-	-	-	-	-	19	-	-	-	-
Delftia acidovorans	53	-	12	36	-	-	-	-	-	-	-
Gordonia rubripertincta	-	-	-	-	-	-	-	-	37	-	-
Herbaspirillum huttiense	36	-	-	22	-	-	-	-	-	-	-
Lysinibacillus sphaericus	-	-	-	-	-	-	-	-	141	-	-
Micrococcus luteus	22	-	-	-	-	-	-	-	-	-	-
Nesterenkonia lacusekhoensis	-	-	-	-	-	5	-	-	-	-	-
Ochrobactrum grignonense	-	-	14	-	-	-	-	-	-	-	-
Pelomonas saccharophila	-	-	-	-	-	-	7	-	-	-	-
Pseudomonas migulae	-	-	4	-	-	-	-	-	-	-	-
Rhizobium tropici	27	-	-	-	-	-	-	-	-	-	-
Sphingomonas aquatilis	-	-	-	14	-	-	4	-	-	-	-
Sphingomonas echinoides	-	-	-	17	-	-	-	-	23	-	-
Staphylococcus epidermidis	-	-	-	-	-	-	2	-	-	-	-
Stenotrophomonas maltophilia	-	-	7	-	-	-	2	-	-	-	-
Thauera selenatis	-	-	-	16	-	-	-	-	-	-	-
Undibacterium oligocarboniphilum	-	-	-	-	-	-	4	-	-	-	-
**TOTAL**	**141**	**0**	**37**	**105**	**0**	**5**	**38**	**0**	**443**	**0**	**0**

5. Supplementary figures

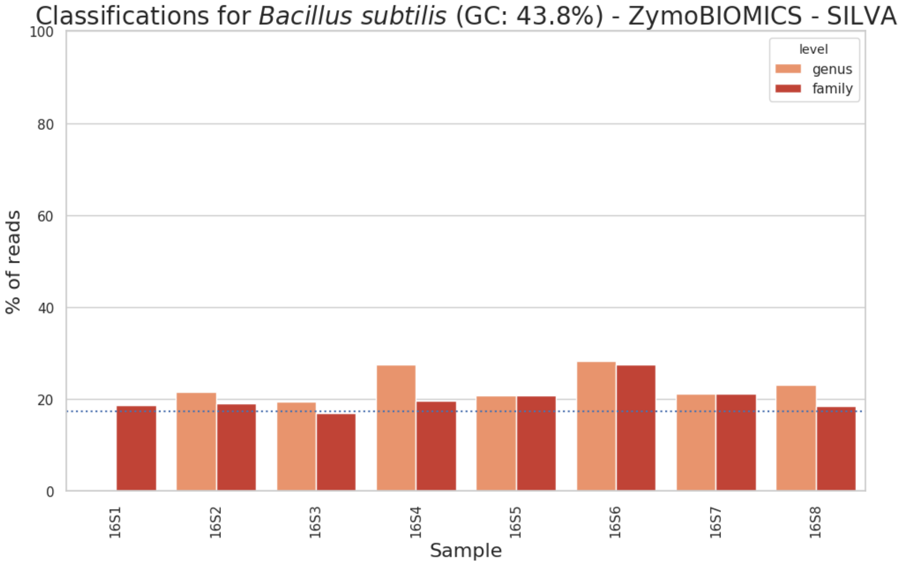

Figure S1. Overview of the correct identification of *Bacillus subtilis* in the different samples when using the SILVA database for the MiSeq data. Bars represent the percentages of reads that are correctly identified at the indicated taxonomic level. The dotted line represents the fraction of 16S rRNA present for this organism in the mock community.

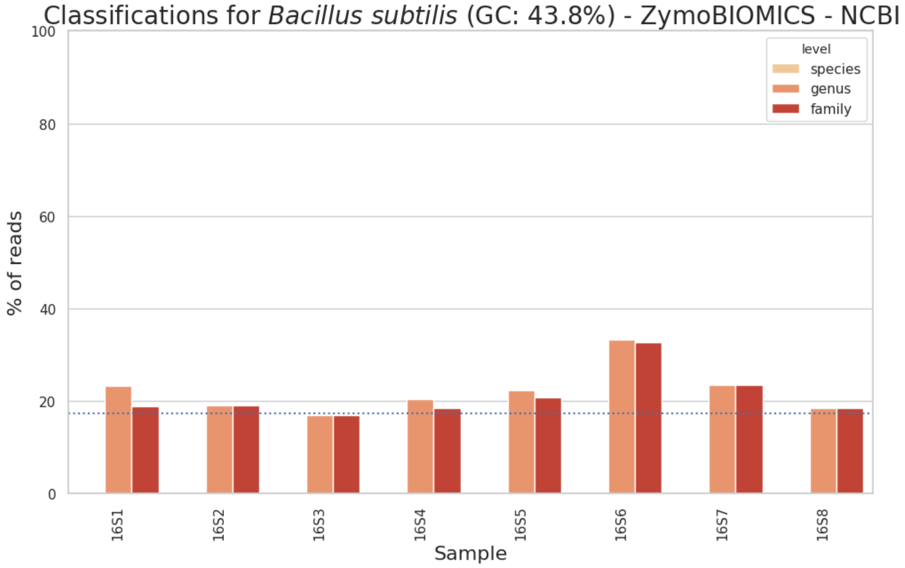

,

Figure S2. Overview of the correct identification of *Bacillus subtilis* in the different samples when using the NCBI 16S database for the MiSeq data. Bars represent the percentages of reads that are correctly identified at the indicated taxonomic level. The dotted line represents the fraction of 16S rRNA present for this organism in the mock community.

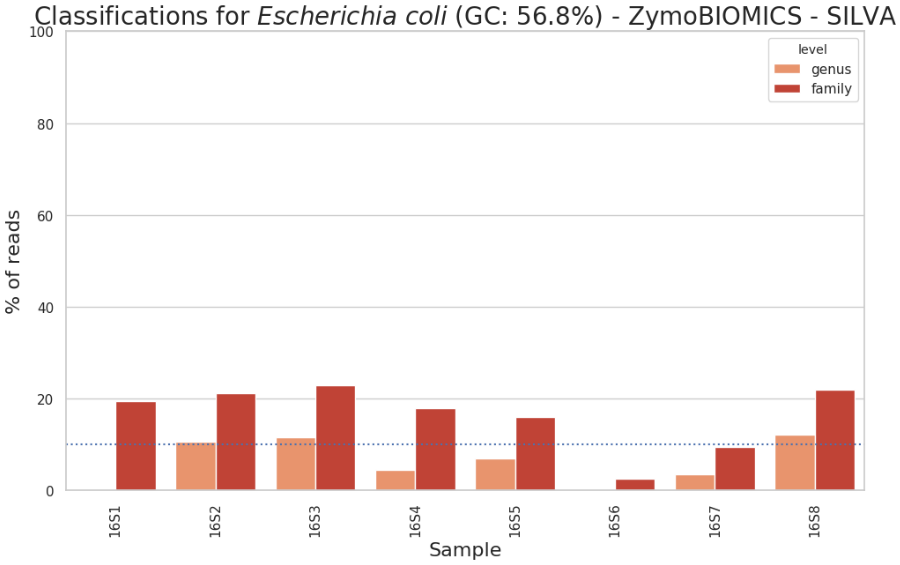

Figure S3. Overview of the correct identification of *Escherichia coli* in the different samples when using the SILVA database for the MiSeq data. Bars represent the percentages of reads that are correctly identified at the indicated taxonomic level. The dotted line represents the fraction of 16S rRNA present for this organism in the mock community.

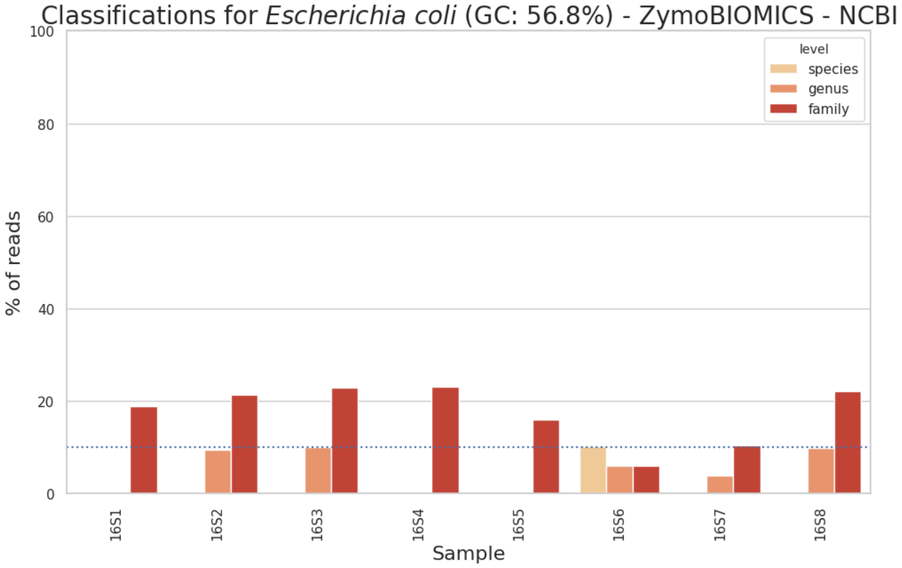

Figure S4. Overview of the correct identification of *Escherichia coli* in the different samples when using the NCBI 16S database for the MiSeq data. Bars represent the percentages of reads that are correctly identified at the indicated taxonomic level. The dotted line represents the fraction of 16S rRNA present for this organism in the mock community.

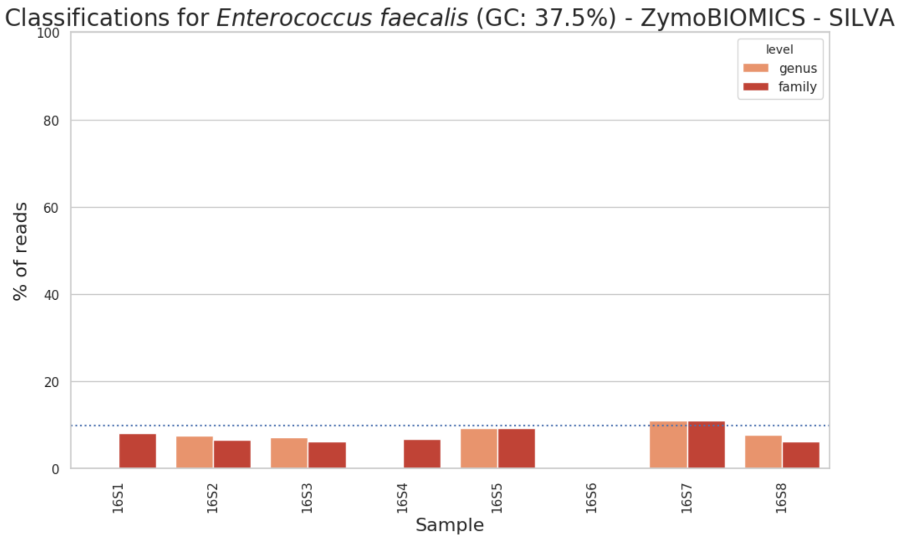

Figure S5. Overview of the correct identification of *Enterococcus faecalis* in the different samples when using the SILVA database for the MiSeq data. Bars represent the percentages of reads that are correctly identified at the indicated taxonomic level. The dotted line represents the fraction of 16S rRNA present for this organism in the mock community.

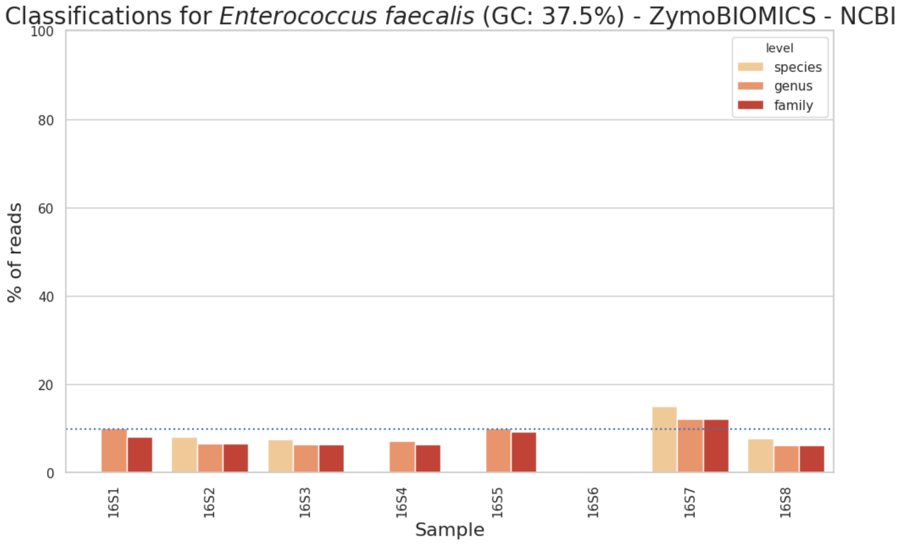

Figure S6. Overview of the correct identification of *Enterococcus faecalis* in the different samples when using the NCBI 16S database for the MiSeq data. Bars represent the percentages of reads that are correctly identified at the indicated taxonomic level. The dotted line represents the fraction of 16S rRNA present for this organism in the mock community.

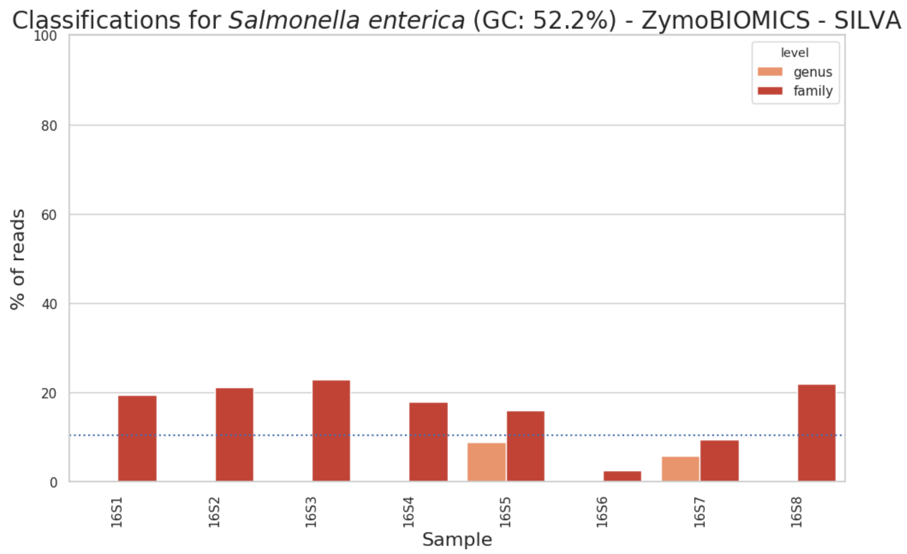

Figure S7. Overview of the correct identification of *Salmonella enterica* in the different samples when using the SILVA database for the MiSeq data. Bars represent the percentages of reads that are correctly identified at the indicated taxonomic level. The dotted line represents the fraction of 16S rRNA present for this organism in the mock community.

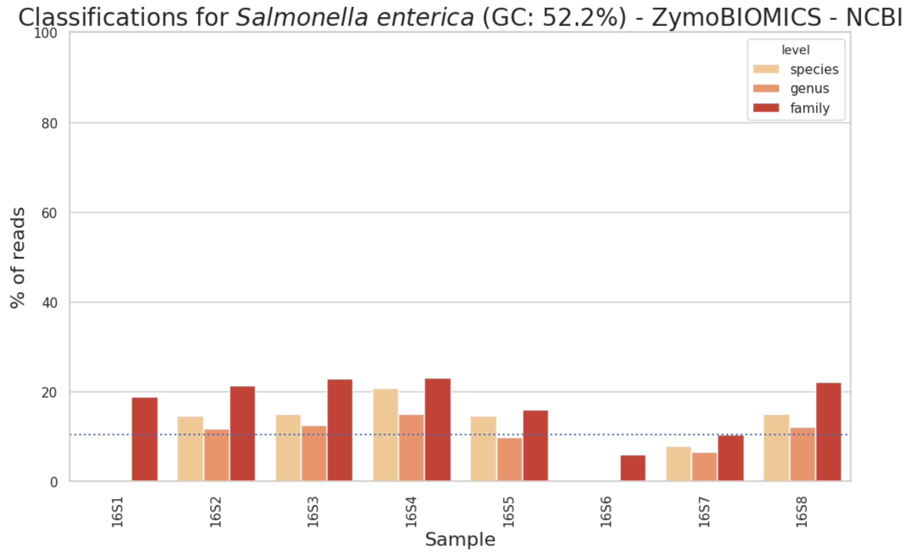

Figure S8. Overview of the correct identification of *Salmonella enterica* in the different samples when using the NCBI 16S database for the MiSeq data. Bars represent the percentages of reads that are correctly identified at the indicated taxonomic level. The dotted line represents the fraction of 16S rRNA present for this organism in the mock community.

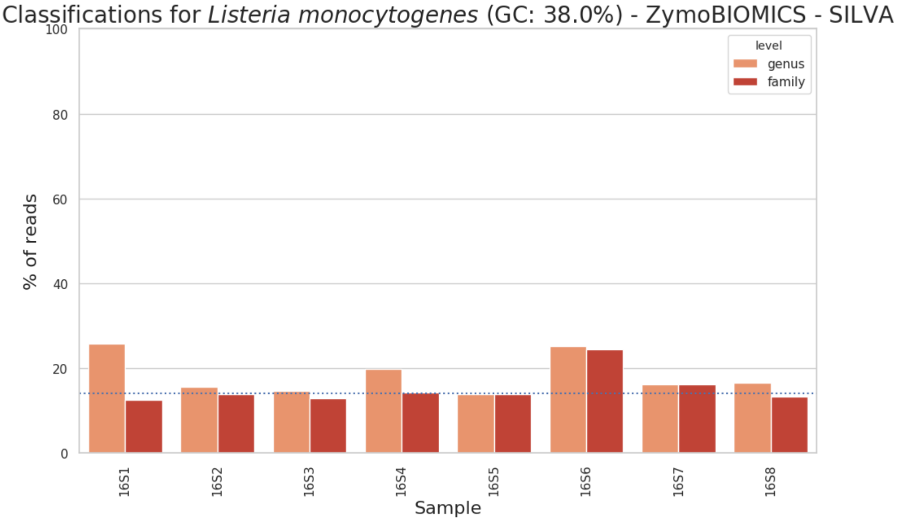

Figure S9. Overview of the correct identification of *Listeria monocytogenes* in the different samples when using the SILVA database for the MiSeq data. Bars represent the percentages of reads that are correctly identified at the indicated taxonomic level. The dotted line represents the fraction of 16S rRNA present for this organism in the mock community.

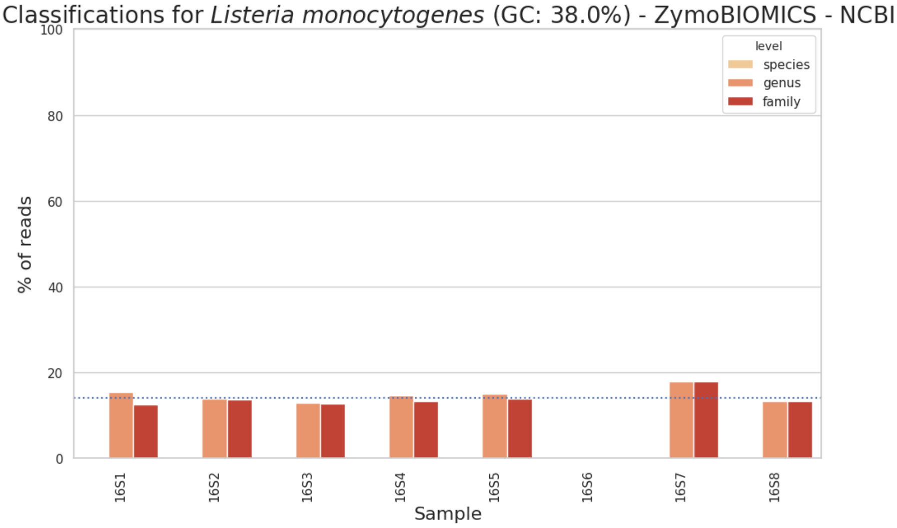

Figure S10. Overview of the correct identification of *Listeria monocytogenes* in the different samples when using the NCBI 16S database for the MiSeq data. Bars represent the percentages of reads that are correctly identified at the indicated taxonomic level. The dotted line represents the fraction of 16S rRNA present for this organism in the mock community.

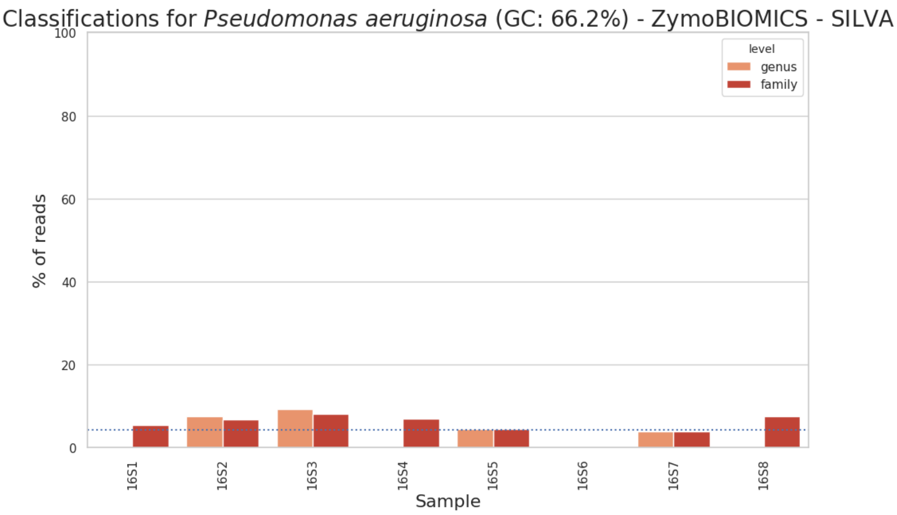

Figure S11. Overview of the correct identification of *Pseudomonas aeruginosa* in the different samples when using the SILVA database for the MiSeq data. Bars represent the percentages of reads that are correctly identified at the indicated taxonomic level. The dotted line represents the fraction of 16S rRNA present for this organism in the mock community.

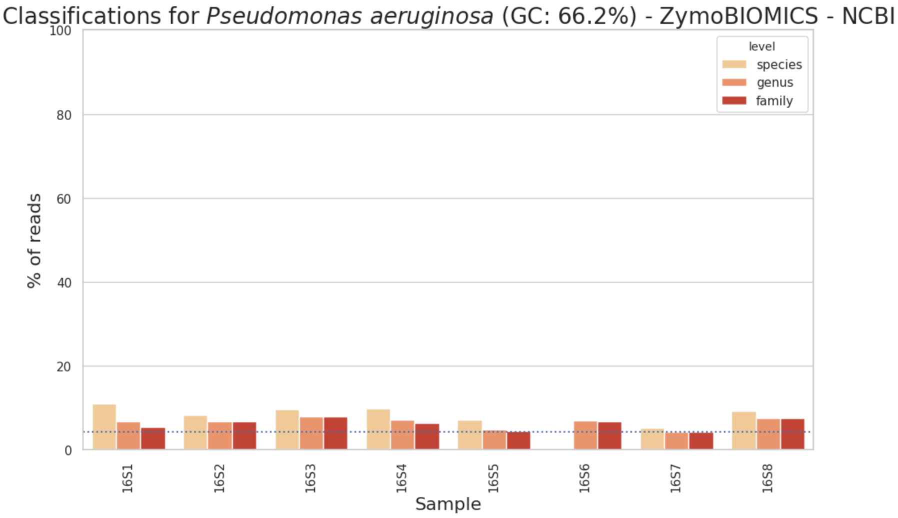

Figure S12. Overview of the correct identification of *Pseudomonas aeruginosa* in the different samples when using the NCBI 16S database for the MiSeq data. Bars represent the percentages of reads that are correctly identified at the indicated taxonomic level. The dotted line represents the fraction of 16S rRNA present for this organism in the mock community.

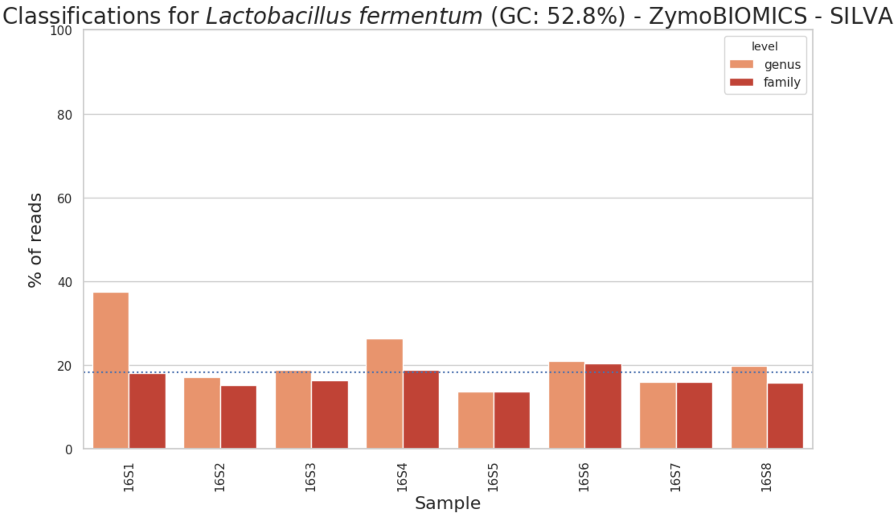

Figure S13. Overview of the correct identification of *Lactobacillus fermentum* in the different samples when using the SILVA database for the MiSeq data. Bars represent the percentages of reads that are correctly identified at the indicated taxonomic level. The dotted line represents the fraction of 16S rRNA present for this organism in the mock community.

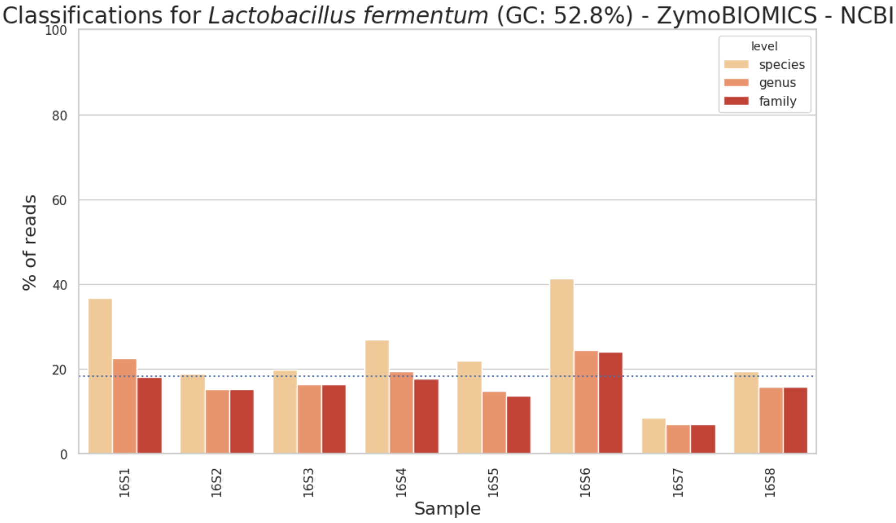

Figure S14. Overview of the correct identification of *Lactobacillus fermentum* in the different samples when using the NCBI 16S database for the MiSeq data. Bars represent the percentages of reads that are correctly identified at the indicated taxonomic level. The dotted line represents the fraction of 16S rRNA present for this organism in the mock community.

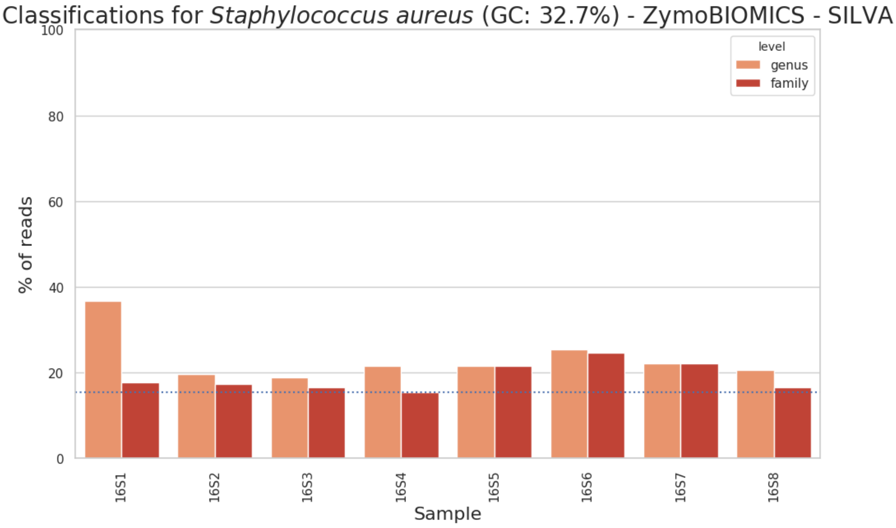

Figure S15. Overview of the correct identification of *Staphylococcus aureus* in the different samples when using the SILVA database for the MiSeq data. Bars represent the percentages of reads that are correctly identified at the indicated taxonomic level. The dotted line represents the fraction of 16S rRNA present for this organism in the mock community.

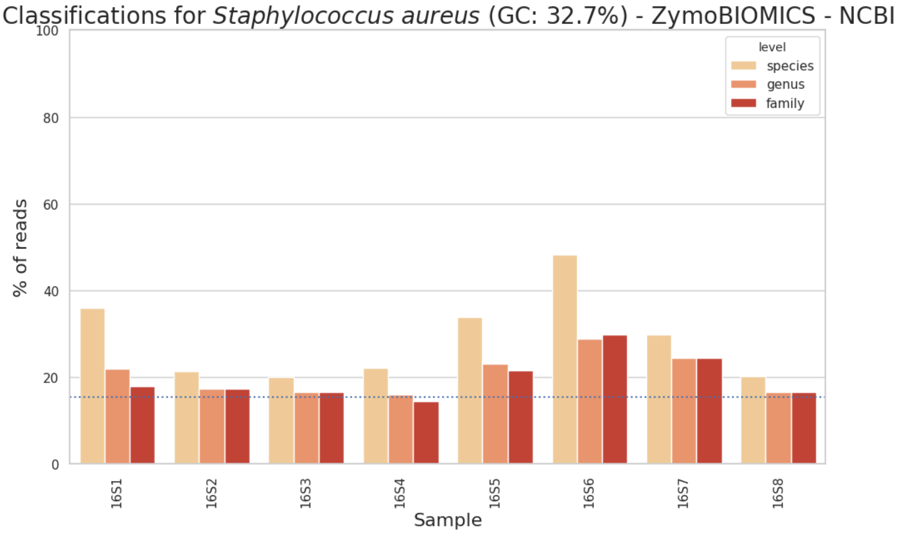

Figure S16. Overview of the correct identification of *Staphylococcus aureus* in the different samples when using the NCBI 16S database for the MiSeq data. Bars represent the percentages of reads that are correctly identified at the indicated taxonomic level. The dotted line represents the fraction of 16S rRNA present for this organism in the mock community.

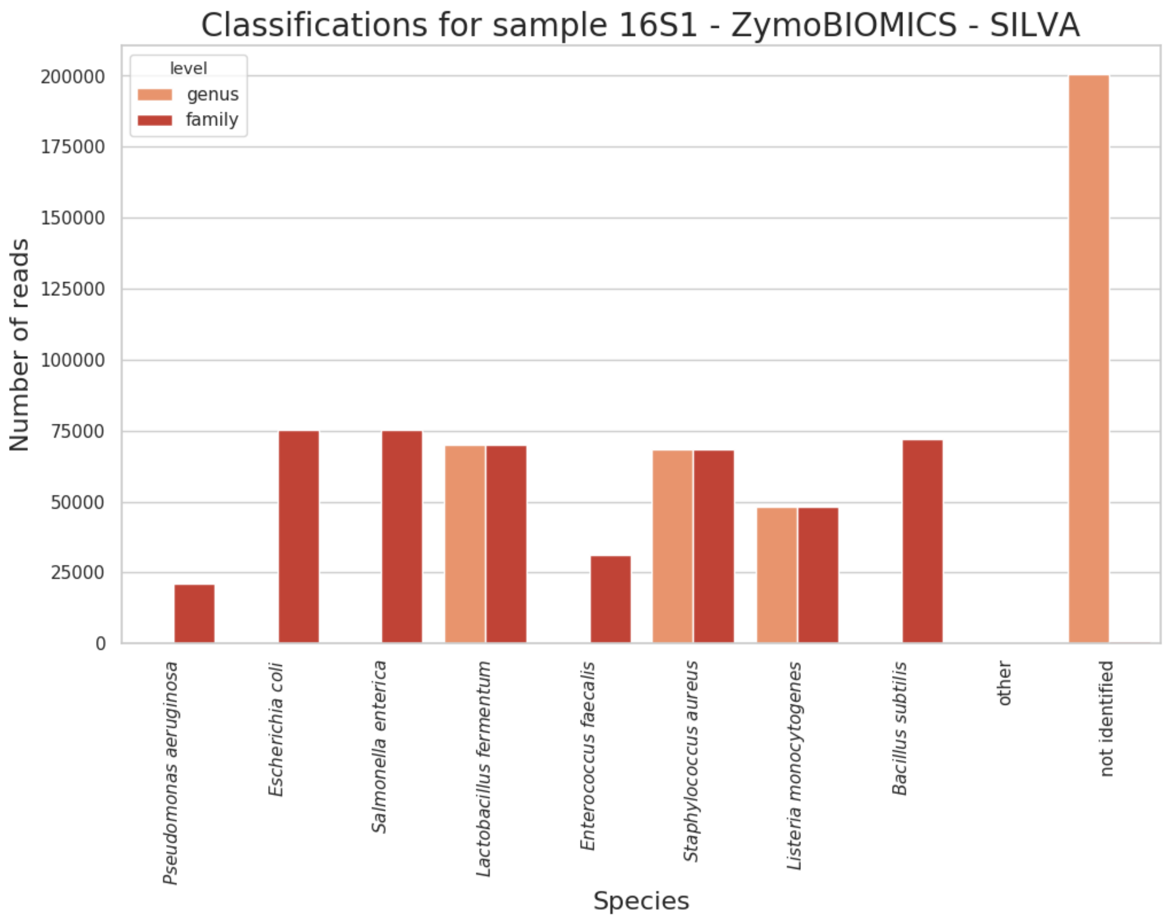

Figure S17. Overview of the number of reads that were correctly identified at the different taxonomic levels for sample 16S1 when using the SILVA database for the MiSeq data. The entry "other" considers all reads that were classified but did not match any of the bacteria present in the sample. The entry "not identified" considers all reads that were not identified at the given taxonomic level but were identified at a higher taxonomic level.

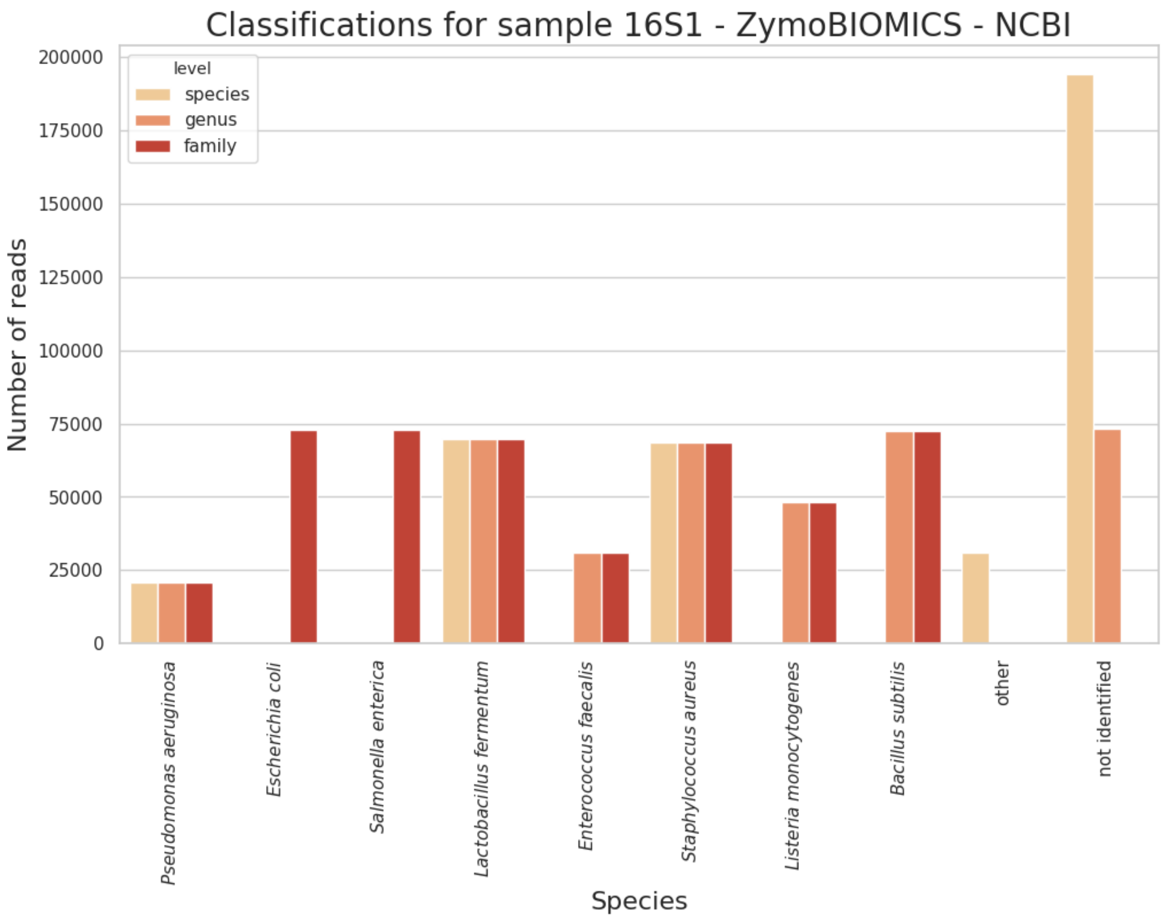

Figure S18. Overview of the number of reads that were correctly identified at the different taxonomic levels for sample 16S1 when using the NCBI 16S database for the MiSeq data. The entry "other" considers all reads that were classified but did not match any of the bacteria present in the sample. The entry "not identified" considers all reads that were not identified at the given taxonomic level but were identified at a higher taxonomic level.

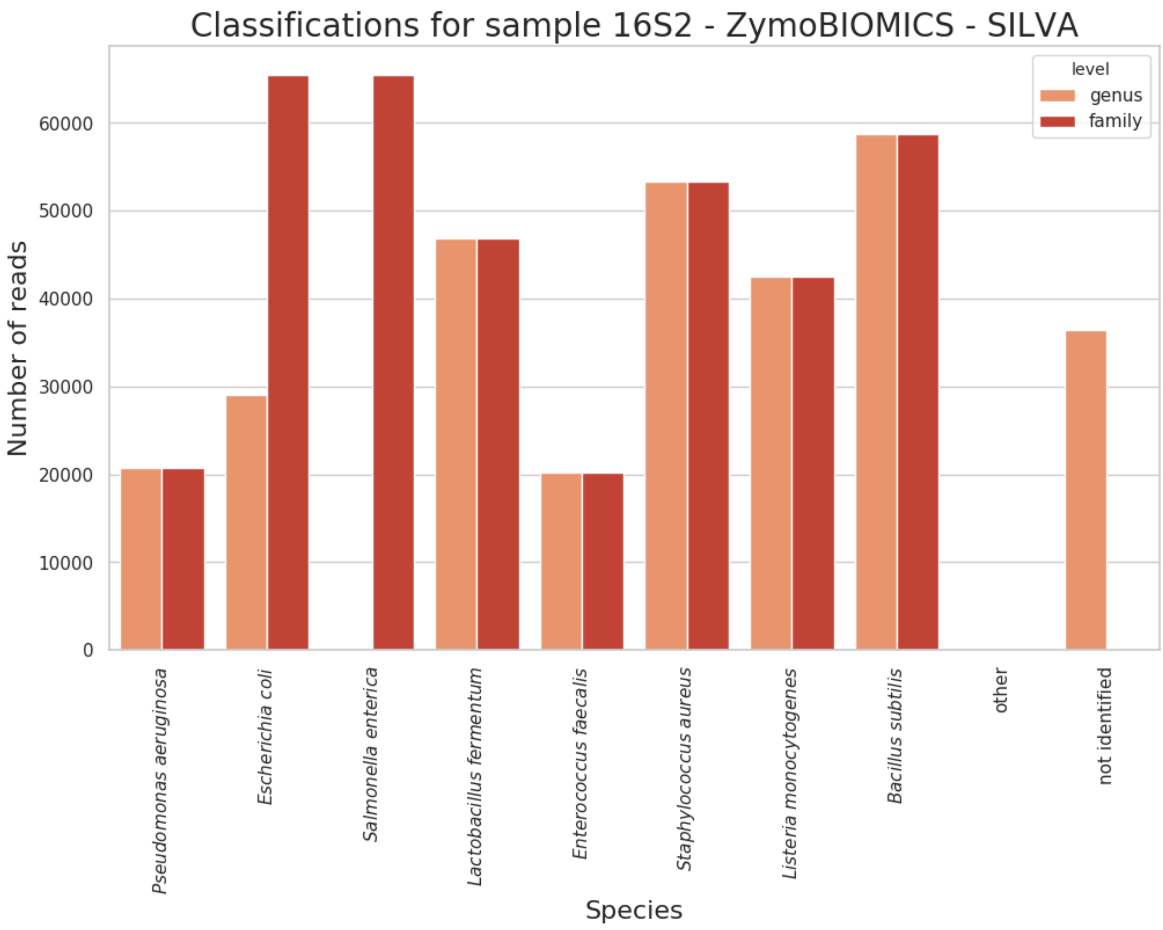

Figure S19. Overview of the number of reads that were correctly identified at the different taxonomic levels for sample 16S2 when using the SILVA database for the MiSeq data. The entry "other" considers all reads that were classified but did not match any of the bacteria present in the sample. The entry "not identified" considers all reads that were not identified at the given taxonomic level but were identified at a higher taxonomic level.

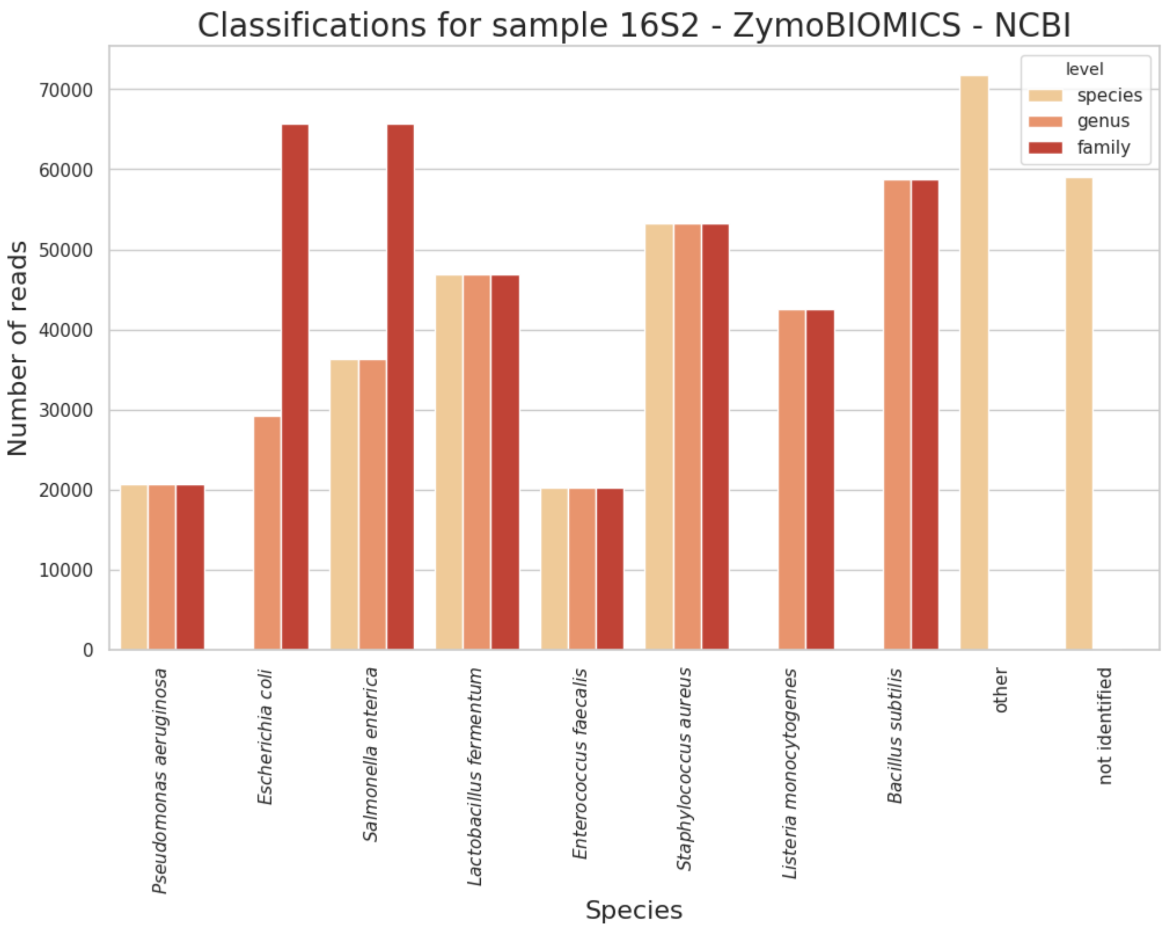

Figure S20. Overview of the number of reads that were correctly identified at the different taxonomic levels for sample 16S2 when using the NCBI 16S database for the MiSeq data. The entry "other" considers all reads that were classified but did not match any of the bacteria present in the sample. The entry "not identified" considers all reads that were not identified at the given taxonomic level but were identified at a higher taxonomic level.

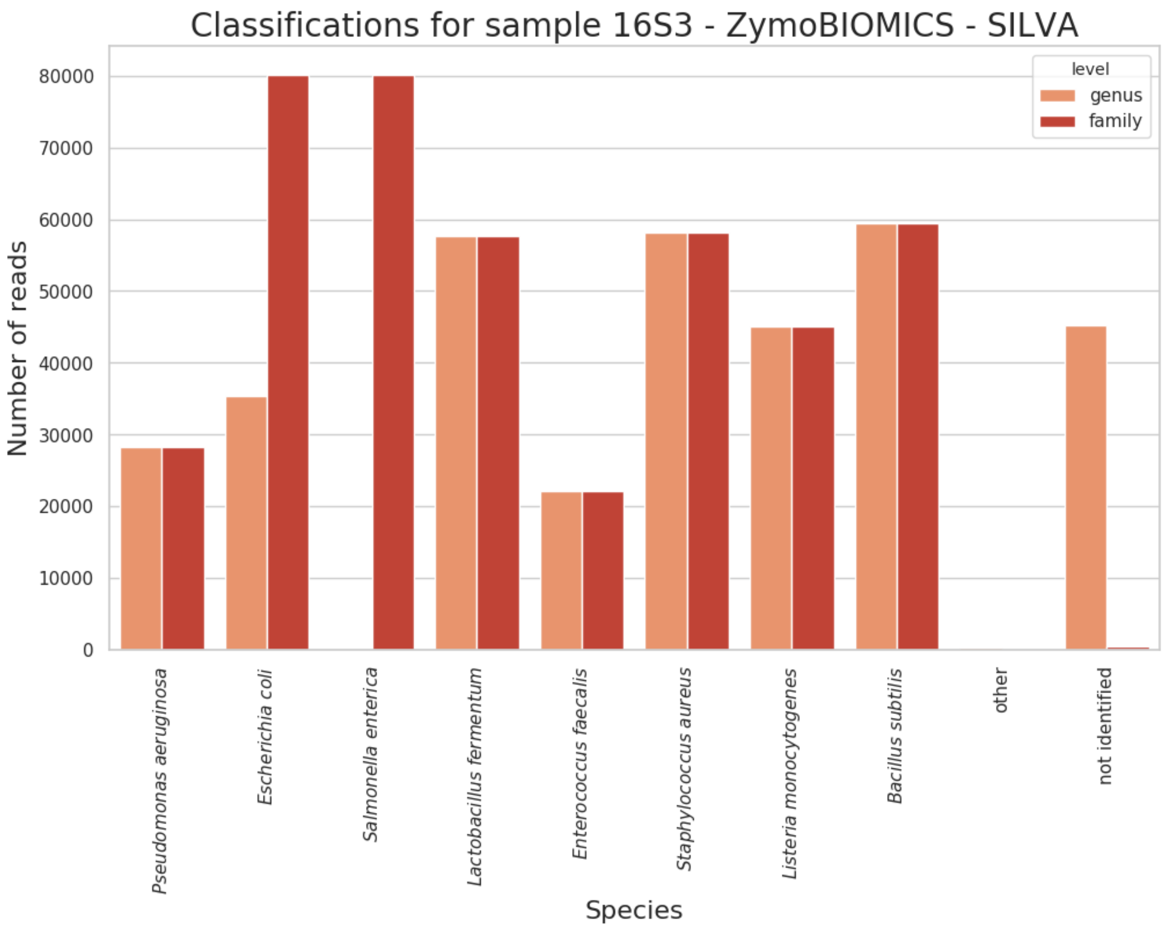

Figure S21. Overview of the number of reads that were correctly identified at the different taxonomic levels for sample 16S3 when using the SILVA database for the MiSeq data. The entry "other" considers all reads that were classified but did not match any of the bacteria present in the sample. The entry "not identified" considers all reads that were not identified at the given taxonomic level but were identified at a higher taxonomic level.

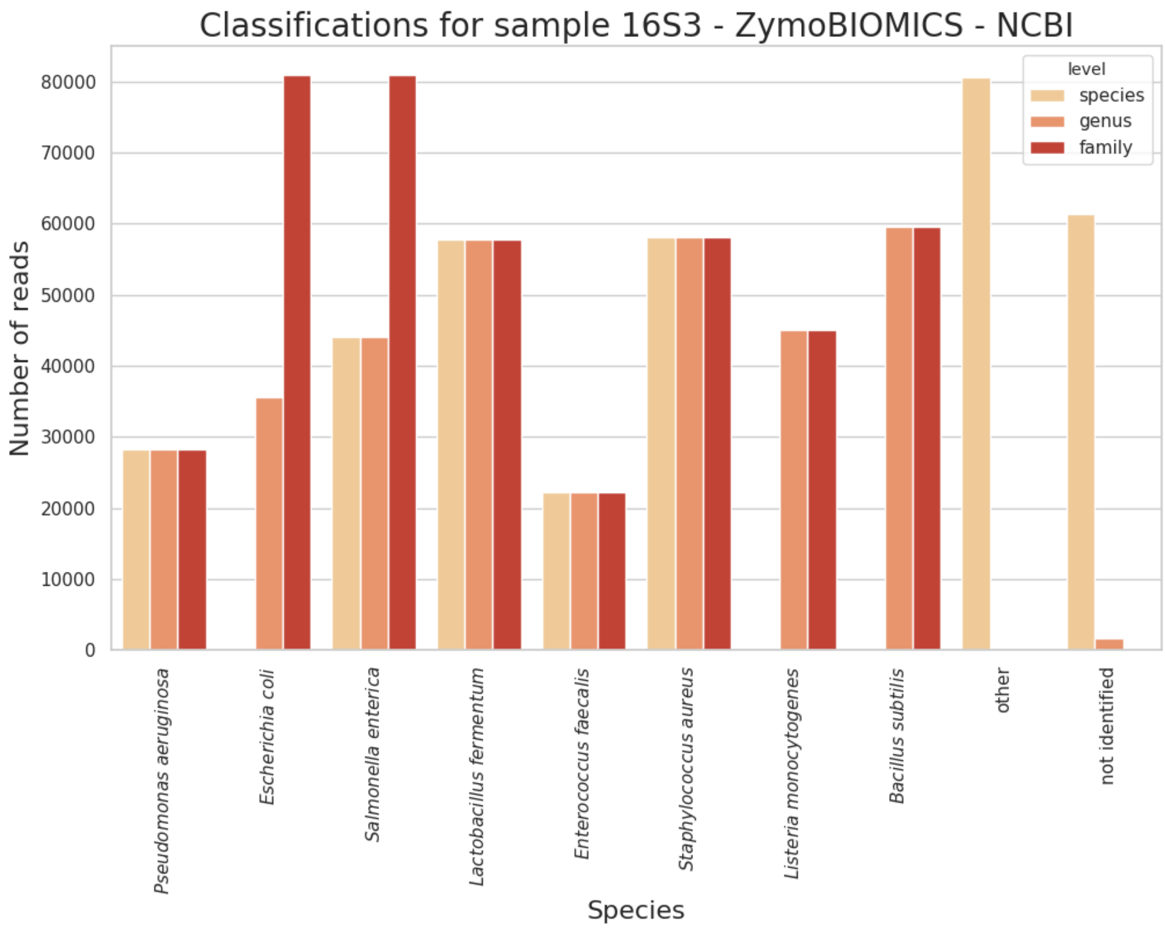

Figure S22. Overview of the number of reads that were correctly identified at the different taxonomic levels for sample 16S3 when using the NCBI 16S database for the MiSeq data. The entry "other" considers all reads that were classified but did not match any of the bacteria present in the sample. The entry "not identified" considers all reads that were not identified at the given taxonomic level but were identified at a higher taxonomic level.

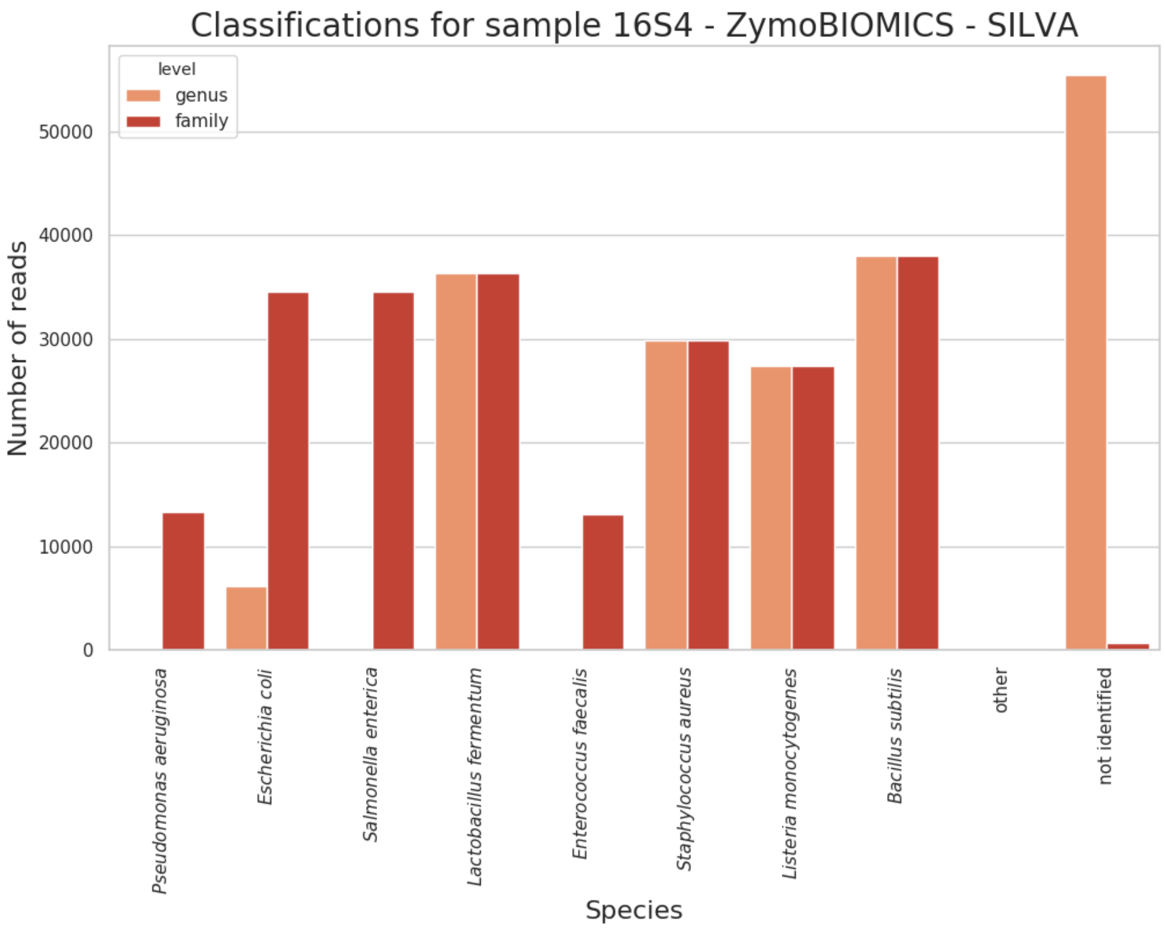

Figure S23. Overview of the number of reads that were correctly identified at the different taxonomic levels for sample 16S4 when using the SILVA database for the MiSeq data. The entry "other" considers all reads that were classified but did not match any of the bacteria present in the sample. The entry "not identified" considers all reads that were not identified at the given taxonomic level but were identified at a higher taxonomic level.

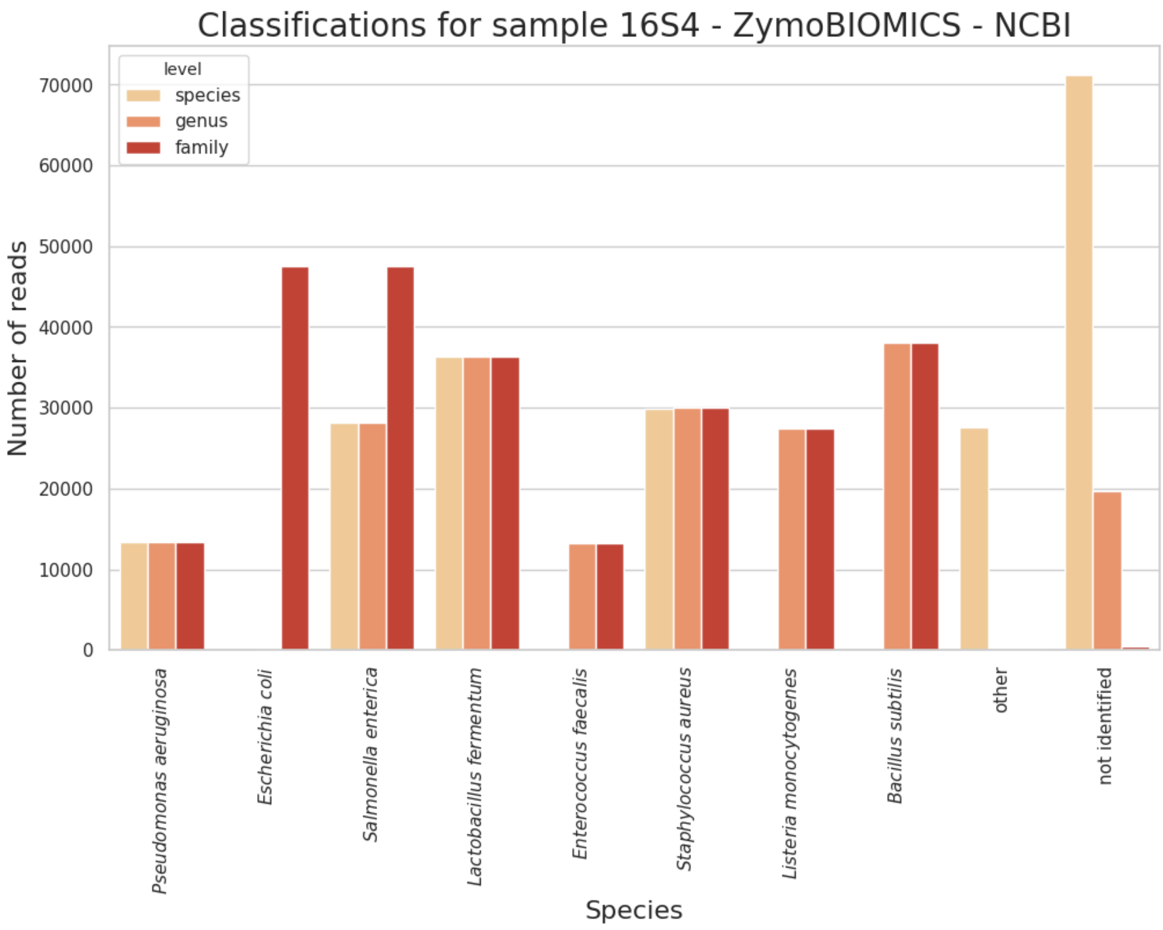

Figure S24. Overview of the number of reads that were correctly identified at the different taxonomic levels for sample 16S4 when using the NCBI 16S database for the MiSeq data. The entry "other" considers all reads that were classified but did not match any of the bacteria present in the sample. The entry "not identified" considers all reads that were not identified at the given taxonomic level but were identified at a higher taxonomic level.

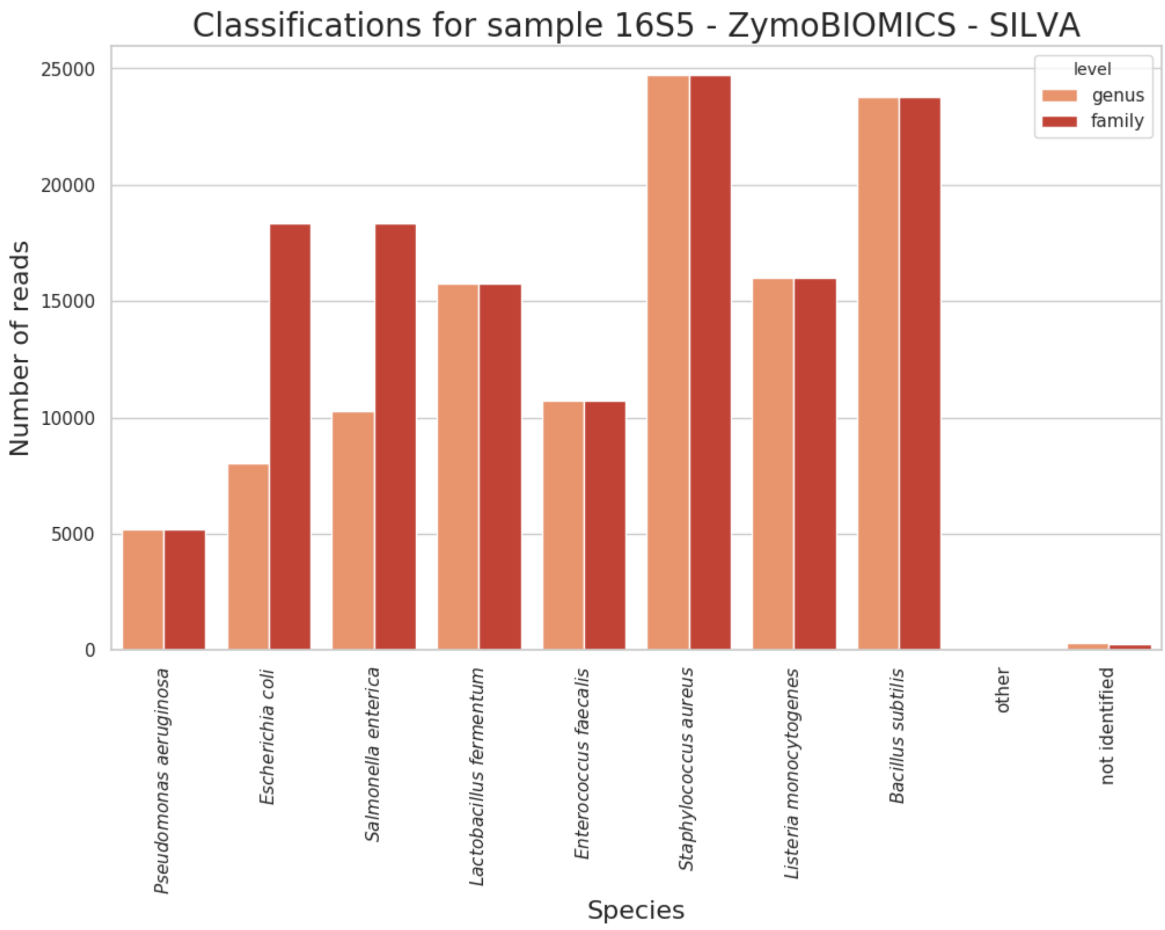

Figure S25. Overview of the number of reads that were correctly identified at the different taxonomic levels for sample 16S5 when using the SILVA database for the MiSeq data. The entry "other" considers all reads that were classified but did not match any of the bacteria present in the sample. The entry "not identified" considers all reads that were not identified at the given taxonomic level but were identified at a higher taxonomic level.

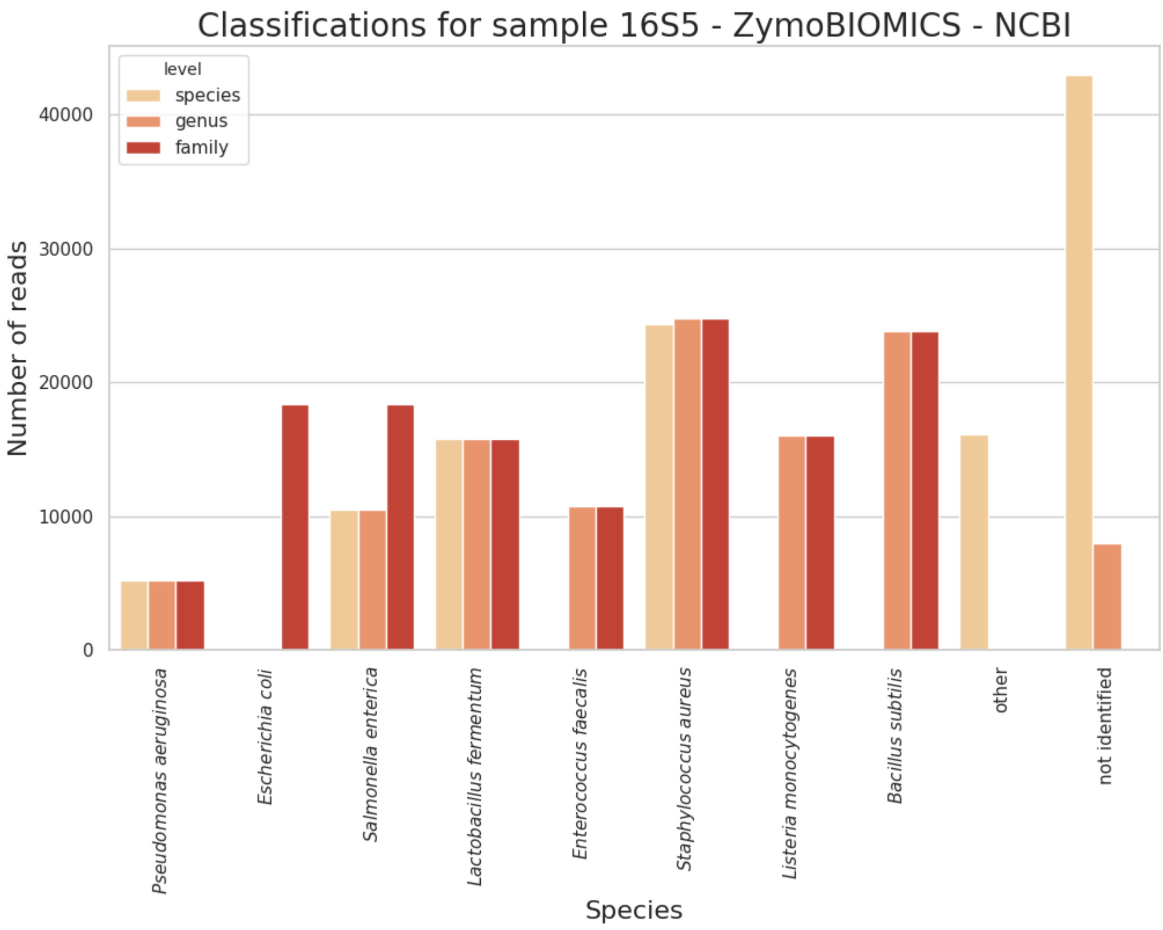

Figure S26. Overview of the number of reads that were correctly identified at the different taxonomic levels for sample 16S5 when using the NCBI 16S database for the MiSeq data. The entry "other" considers all reads that were classified but did not match any of the bacteria present in the sample. The entry "not identified" considers all reads that were not identified at the given taxonomic level but were identified at a higher taxonomic level.

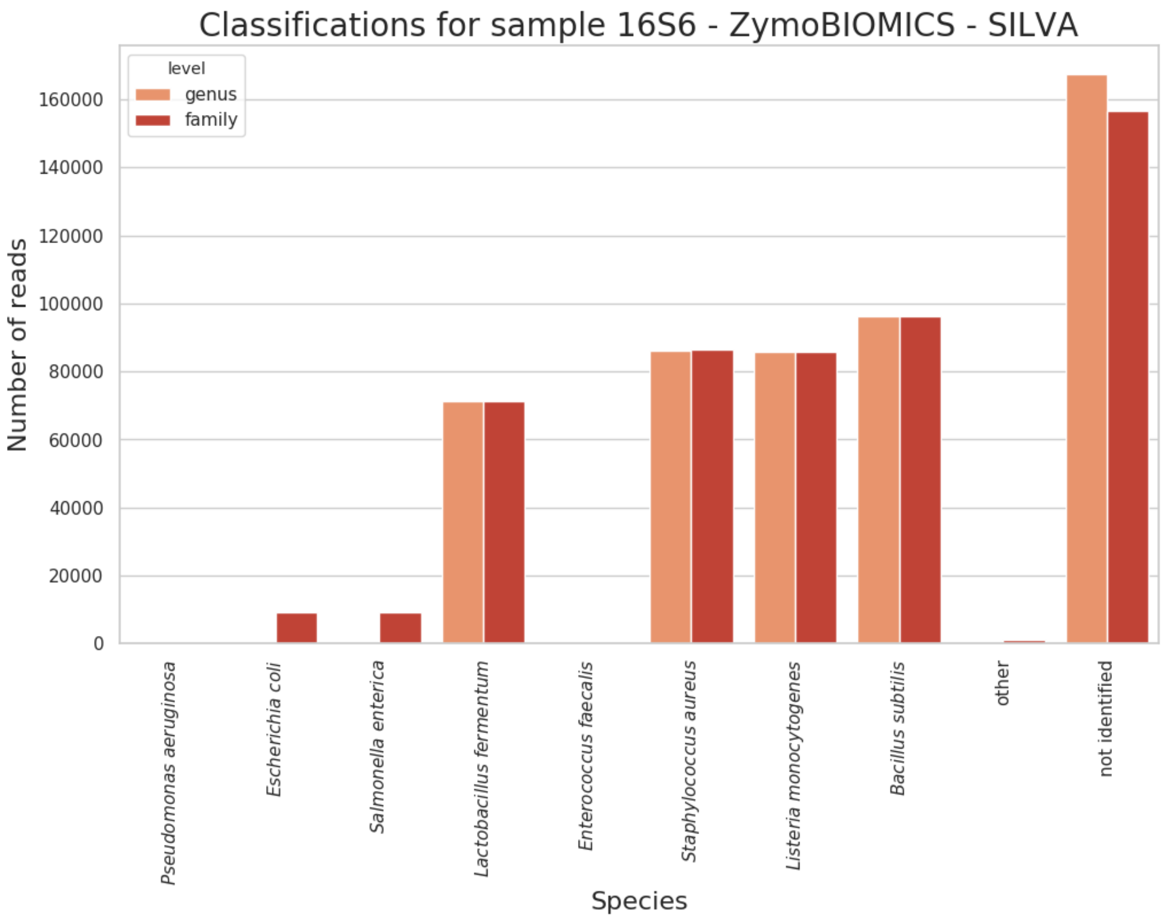

Figure S27. Overview of the number of reads that were correctly identified at the different taxonomic levels for sample 16S6 when using the SILVA database for the MiSeq data. The entry "other" considers all reads that were classified but did not match any of the bacteria present in the sample. The entry "not identified" considers all reads that were not identified at the given taxonomic level but were identified at a higher taxonomic level.

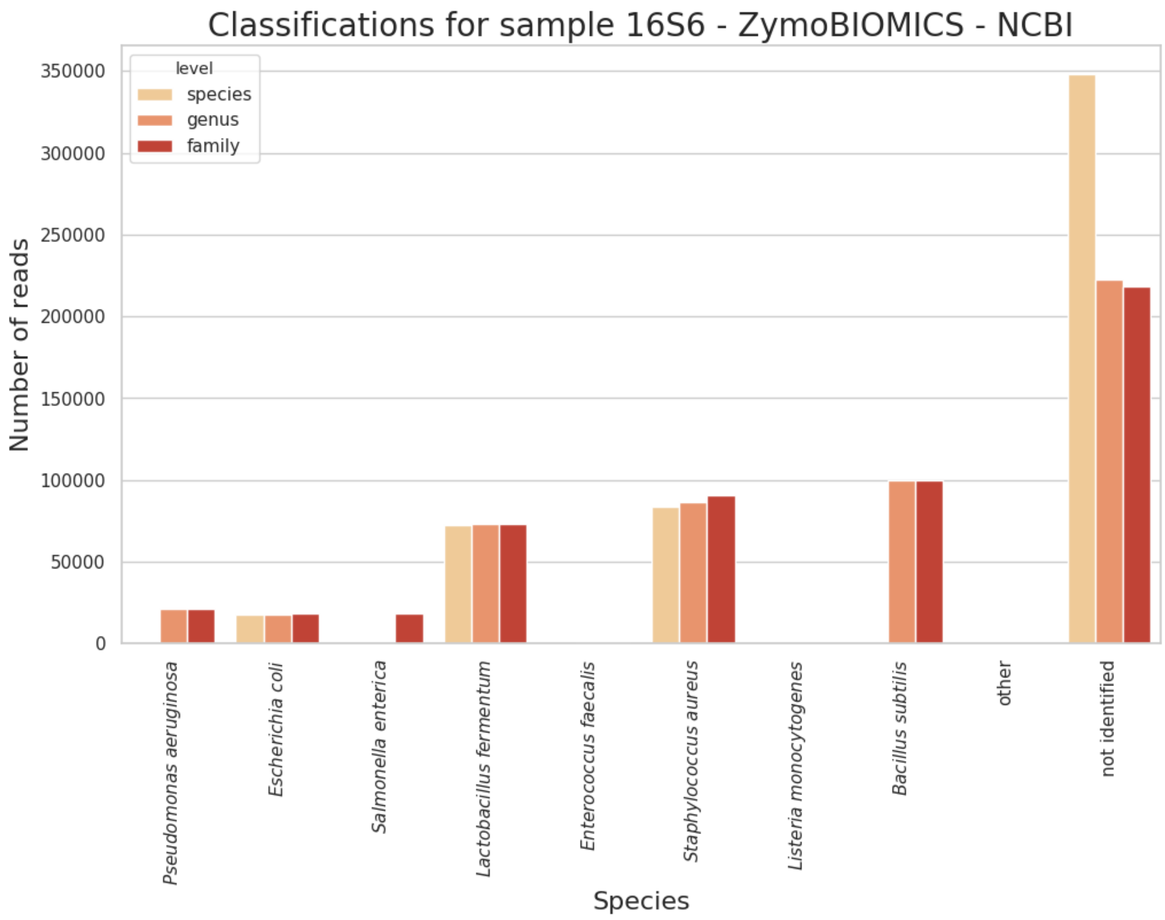

Figure S28. Overview of the number of reads that were correctly identified at the different taxonomic levels for sample 16S6 when using the NCBI 16S database for the MiSeq data. The entry "other" considers all reads that were classified but did not match any of the bacteria present in the sample. The entry "not identified" considers all reads that were not identified at the given taxonomic level but were identified at a higher taxonomic level.

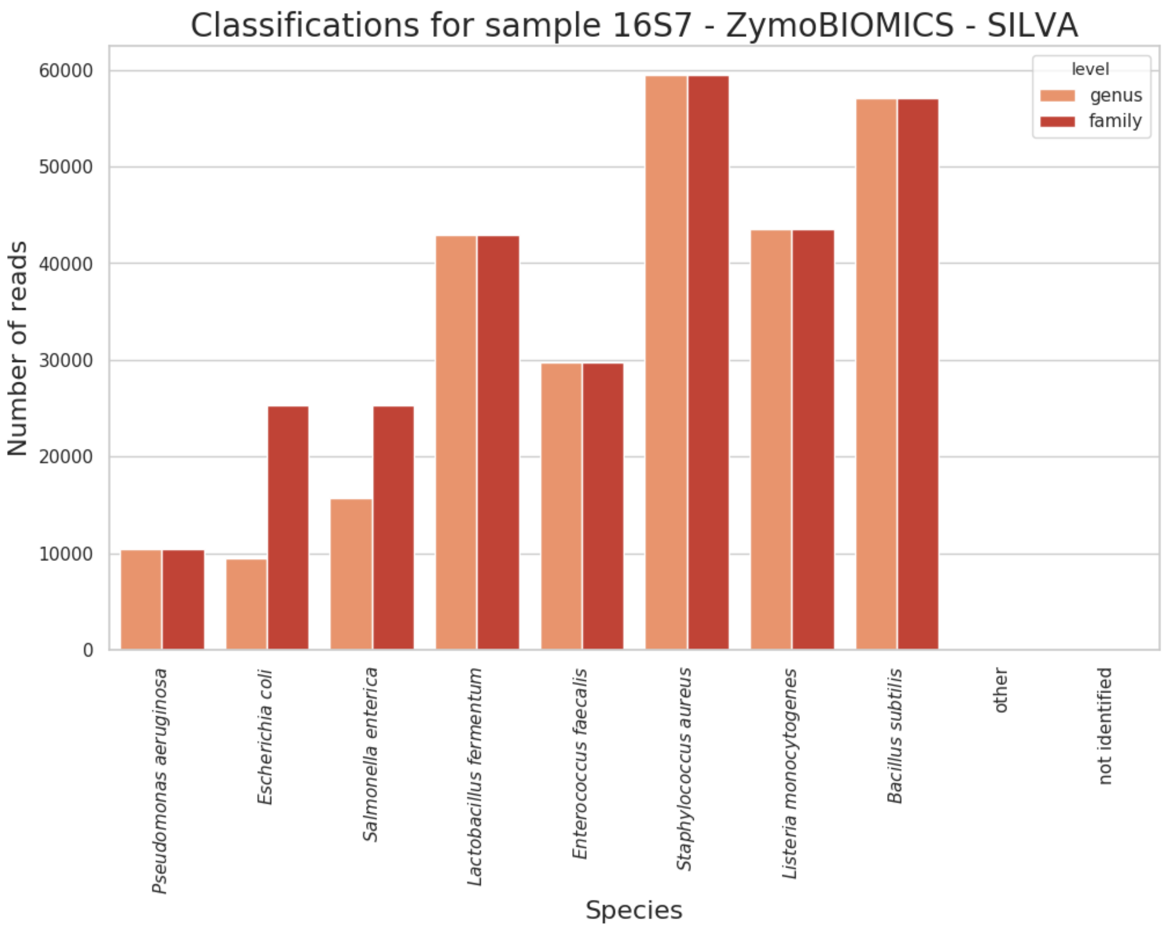

Figure S29. Overview of the number of reads that were correctly identified at the different taxonomic levels for sample 16S7 when using the SILVA database for the MiSeq data. The entry "other" considers all reads that were classified but did not match any of the bacteria present in the sample. The entry "not identified" considers all reads that were not identified at the given taxonomic level but were identified at a higher taxonomic level.

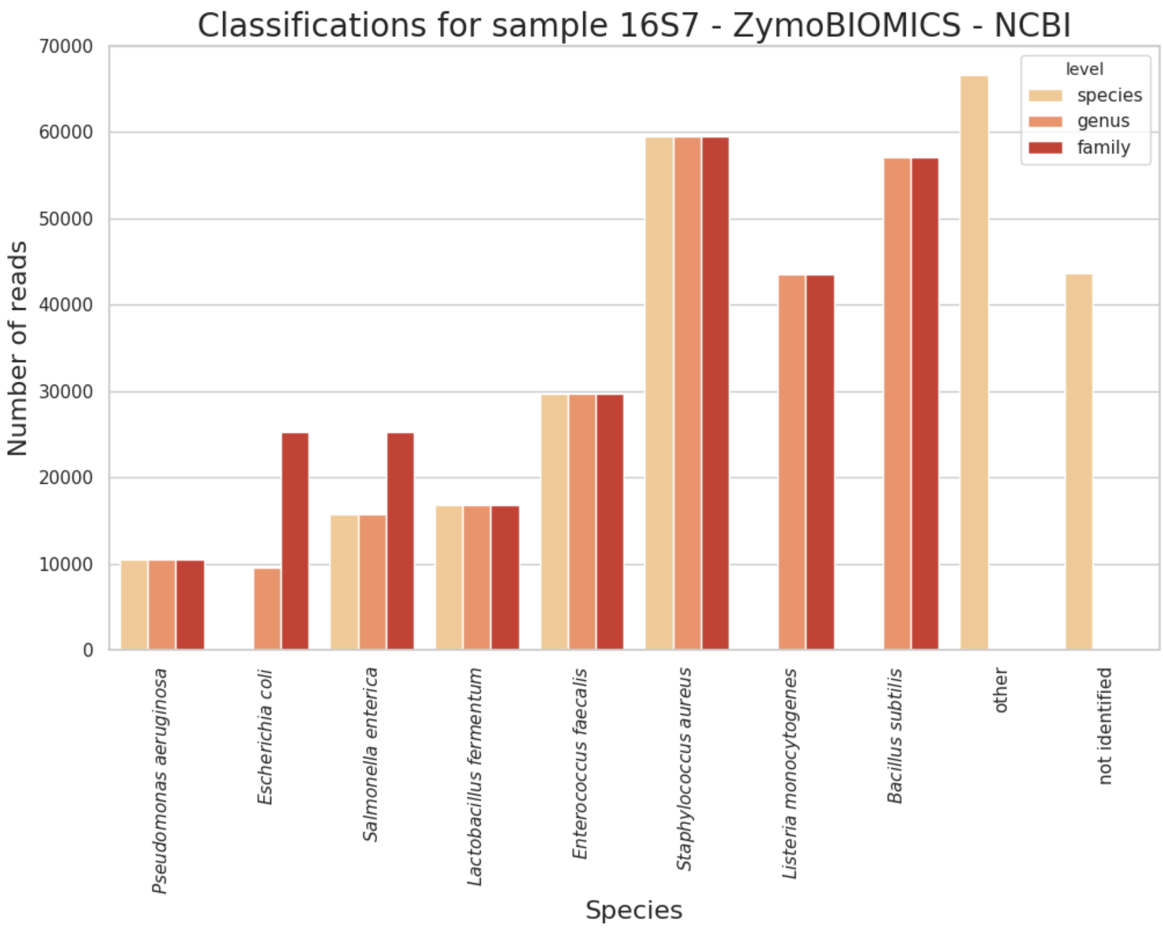

Figure S30. Overview of the number of reads that were correctly identified at the different taxonomic levels for sample 16S7 when using the NCBI 16S database for the MiSeq data. The entry "other" considers all reads that were classified but did not match any of the bacteria present in the sample. The entry "not identified" considers all reads that were not identified at the given taxonomic level but were identified at a higher taxonomic level.

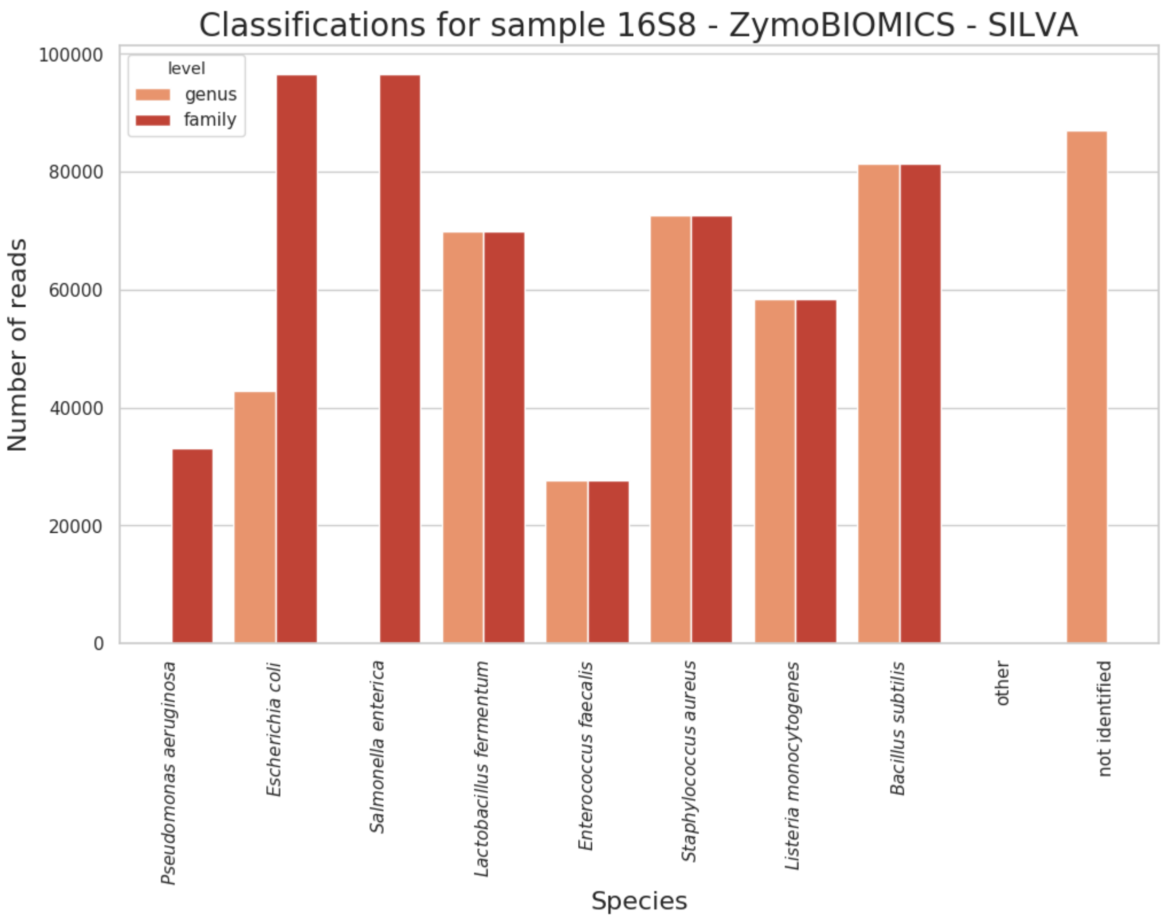

Figure S31. Overview of the number of reads that were correctly identified at the different taxonomic levels for sample 16S8 when using the SILVA database for the MiSeq data. The entry "other" considers all reads that were classified but did not match any of the bacteria present in the sample. The entry "not identified" considers all reads that were not identified at the given taxonomic level but were identified at a higher taxonomic level.

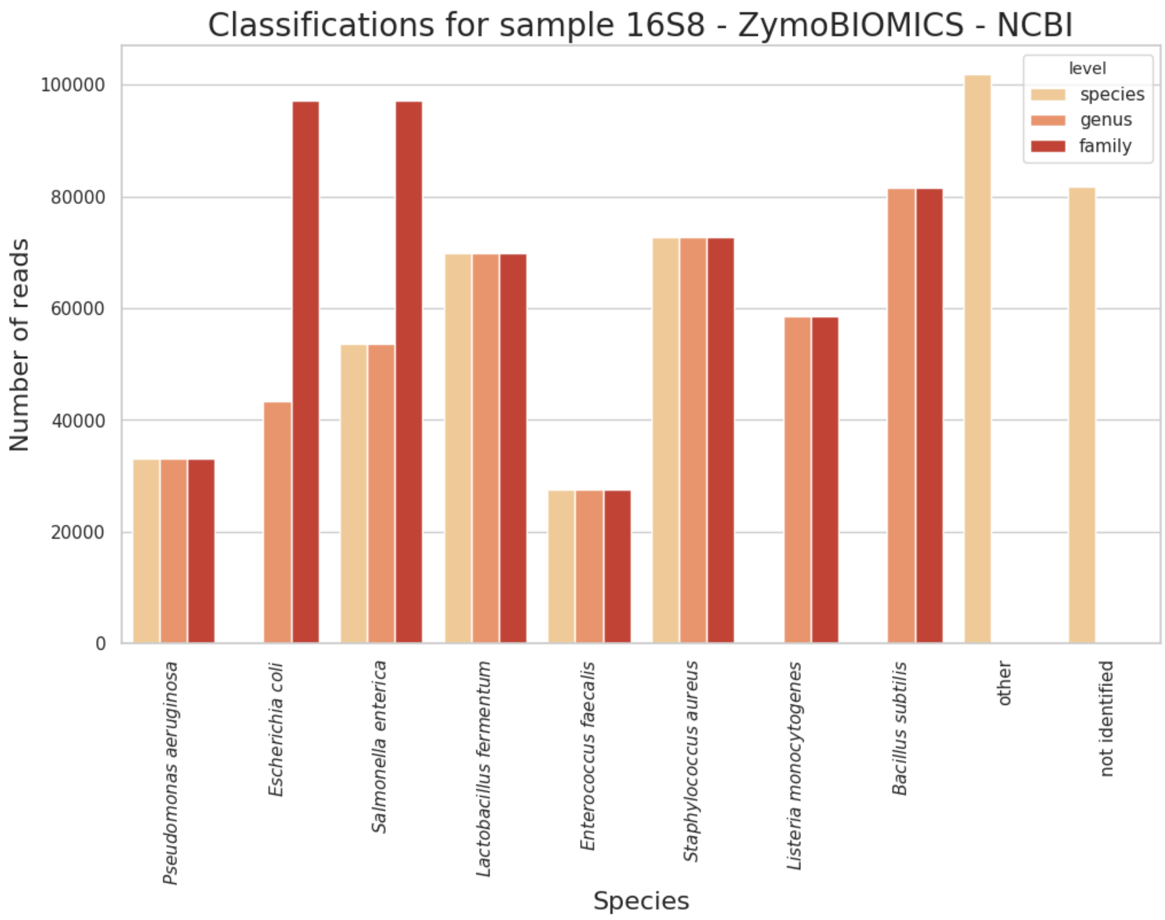

Figure S32. Overview of the number of reads that were correctly identified at the different taxonomic levels for sample 16S8 when using the NCBI 16S database for the MiSeq data. The entry "other" considers all reads that were classified but did not match any of the bacteria present in the sample. The entry "not identified" considers all reads that were not identified at the given taxonomic level but were identified at a higher taxonomic level.

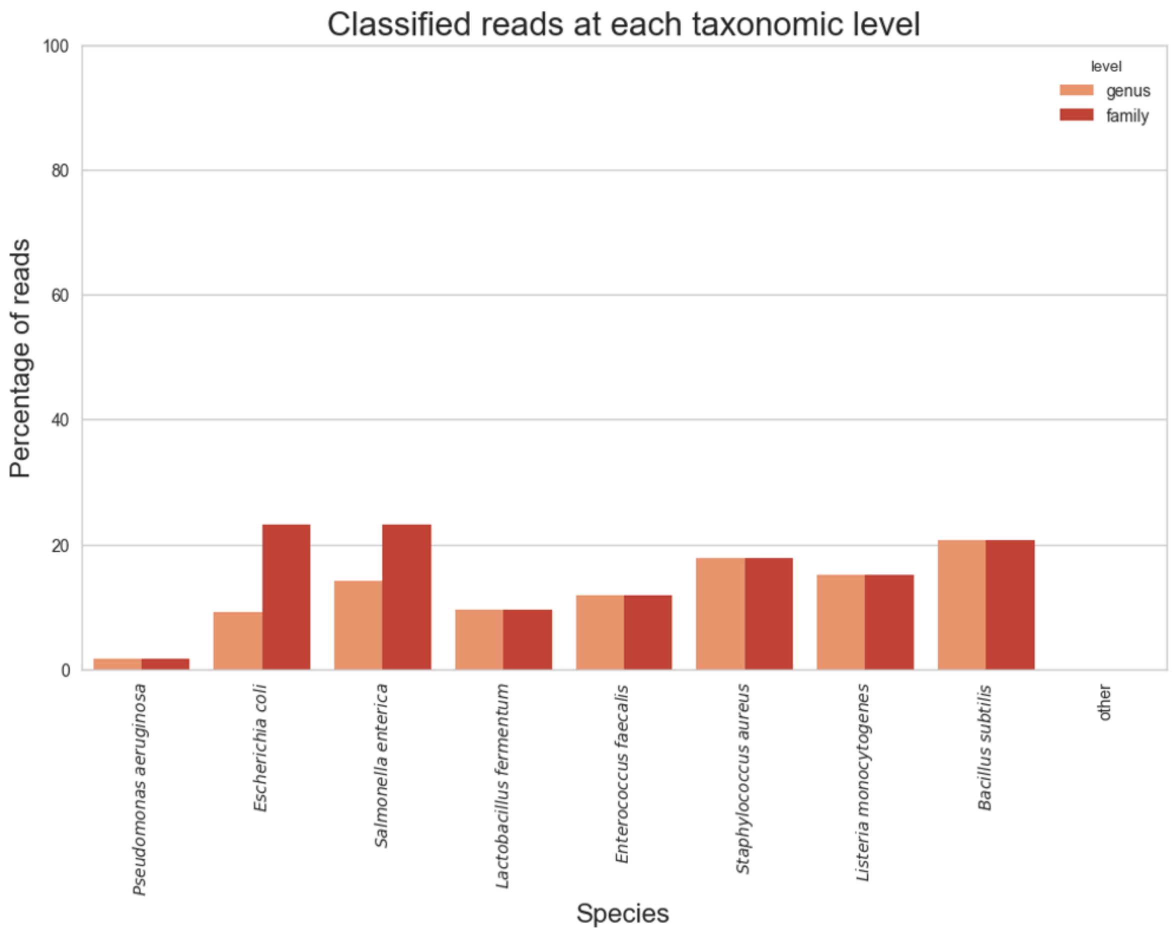

Figure S33. Overview of the percentages of reads that were correctly identified at the different taxonomic levels when using the Mothur workflow and SILVA database for the MinION data. All percentages were calculated against the number of classified reads at that level.

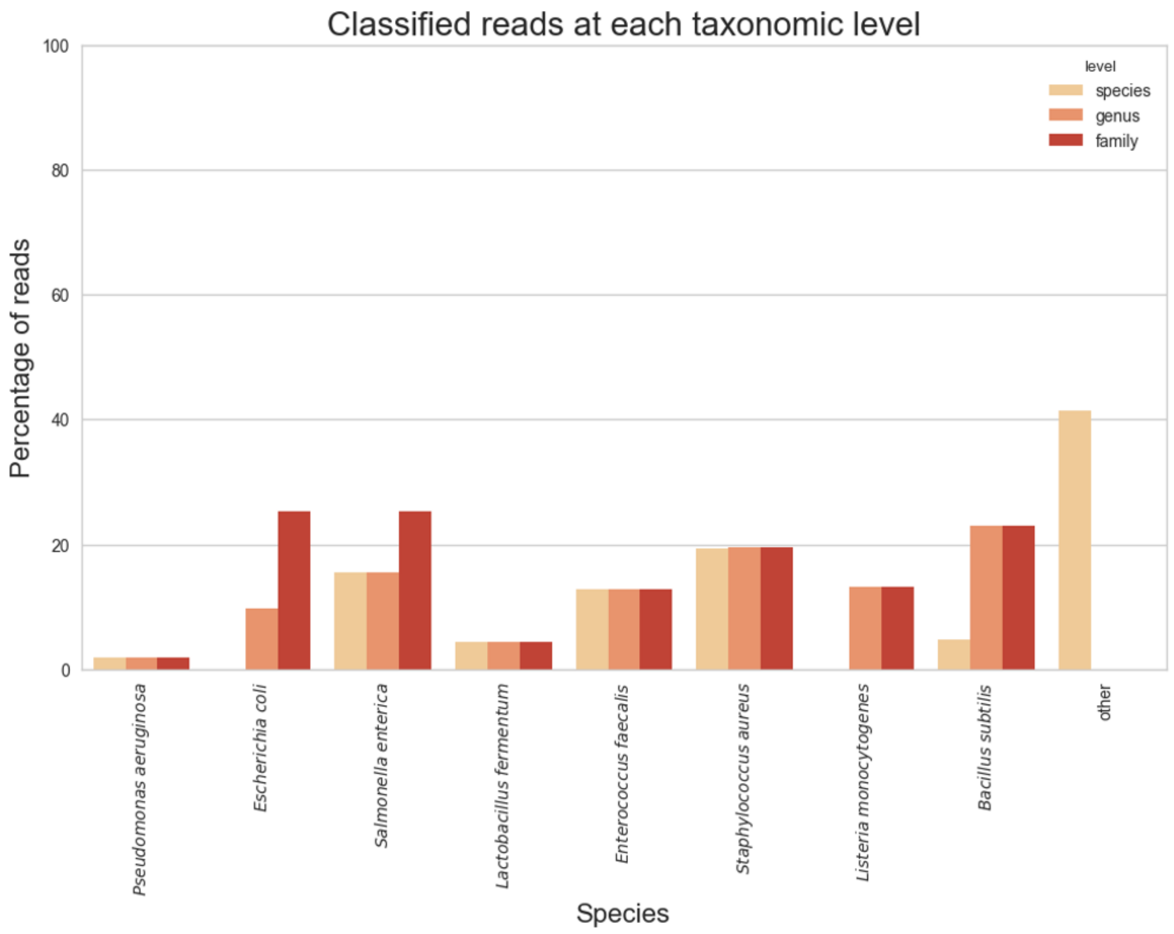

Figure S34. Overview of the percentages of reads that were correctly identified at the different taxonomic levels when using the Mothur workflow and NCBI 16S database for the MinION data. All percentages were calculated against the number of classified reads at that level.

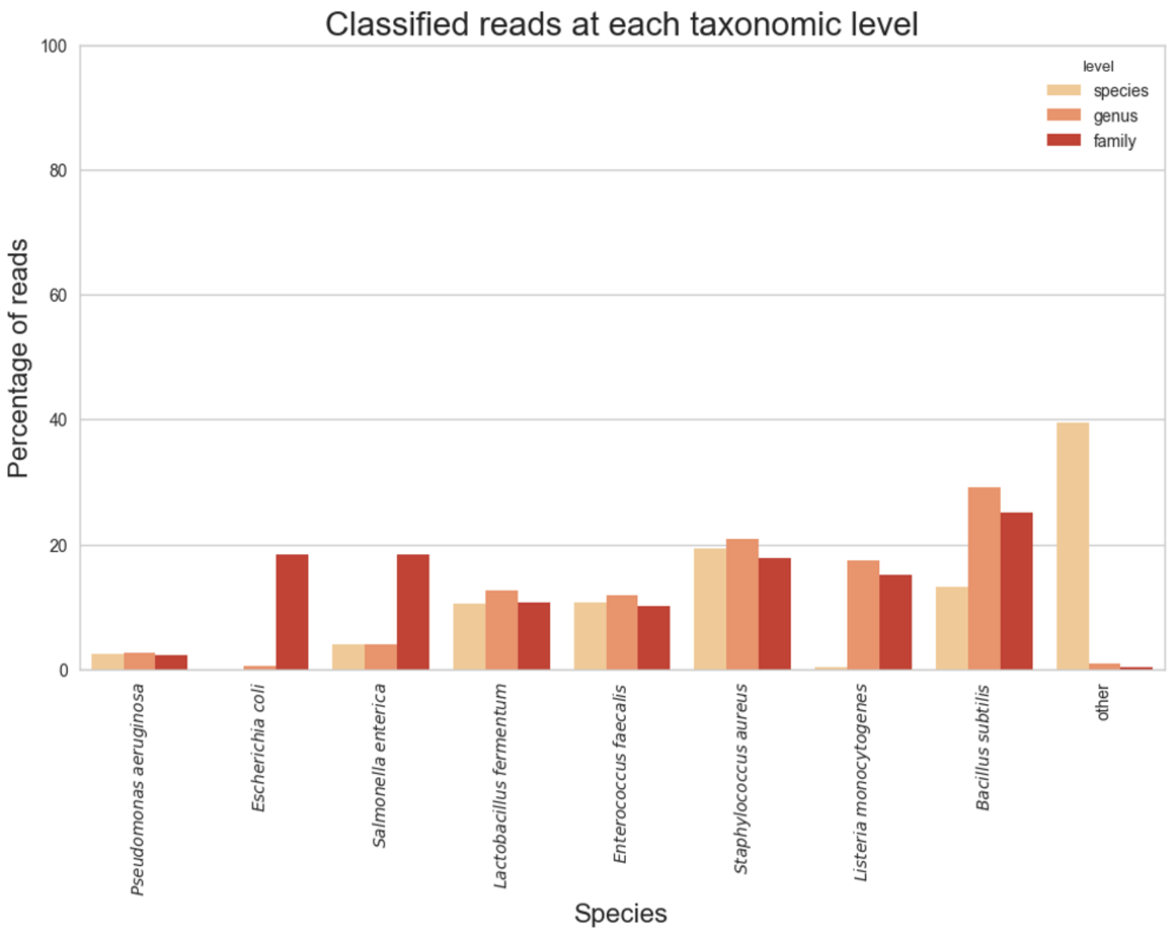

Figure S35. Overview of the percentages of reads that were correctly identified at the different taxonomic levels when using the EPI2ME workflow and NCBI 16S database for the MinION data. All percentages were calculated against the number of classified reads at that level.

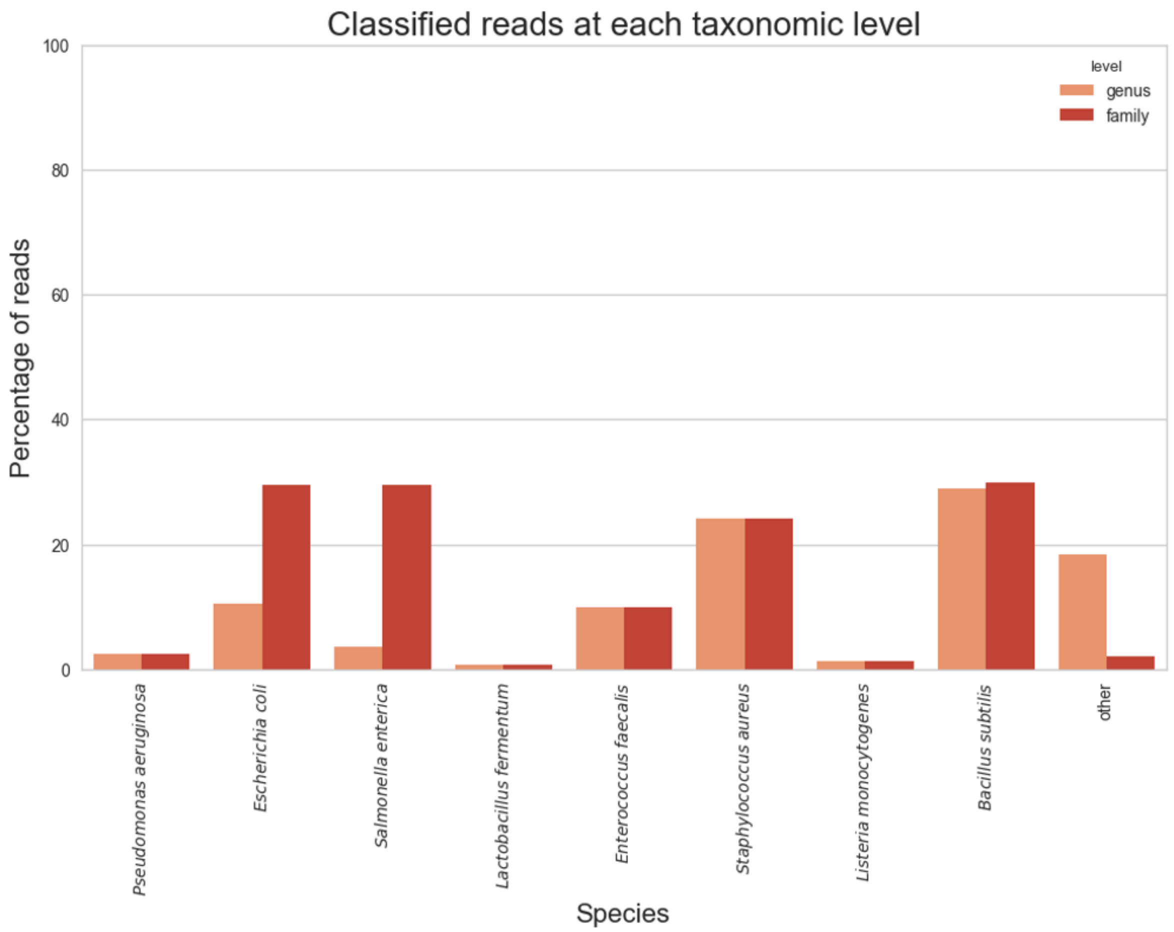

Figure S36. Overview of the percentages of reads that were correctly identified at the different taxonomic levels when using the GraphMap workflow and SILVA database for the MinION data. All percentages were calculated against the number of classified reads at that level.

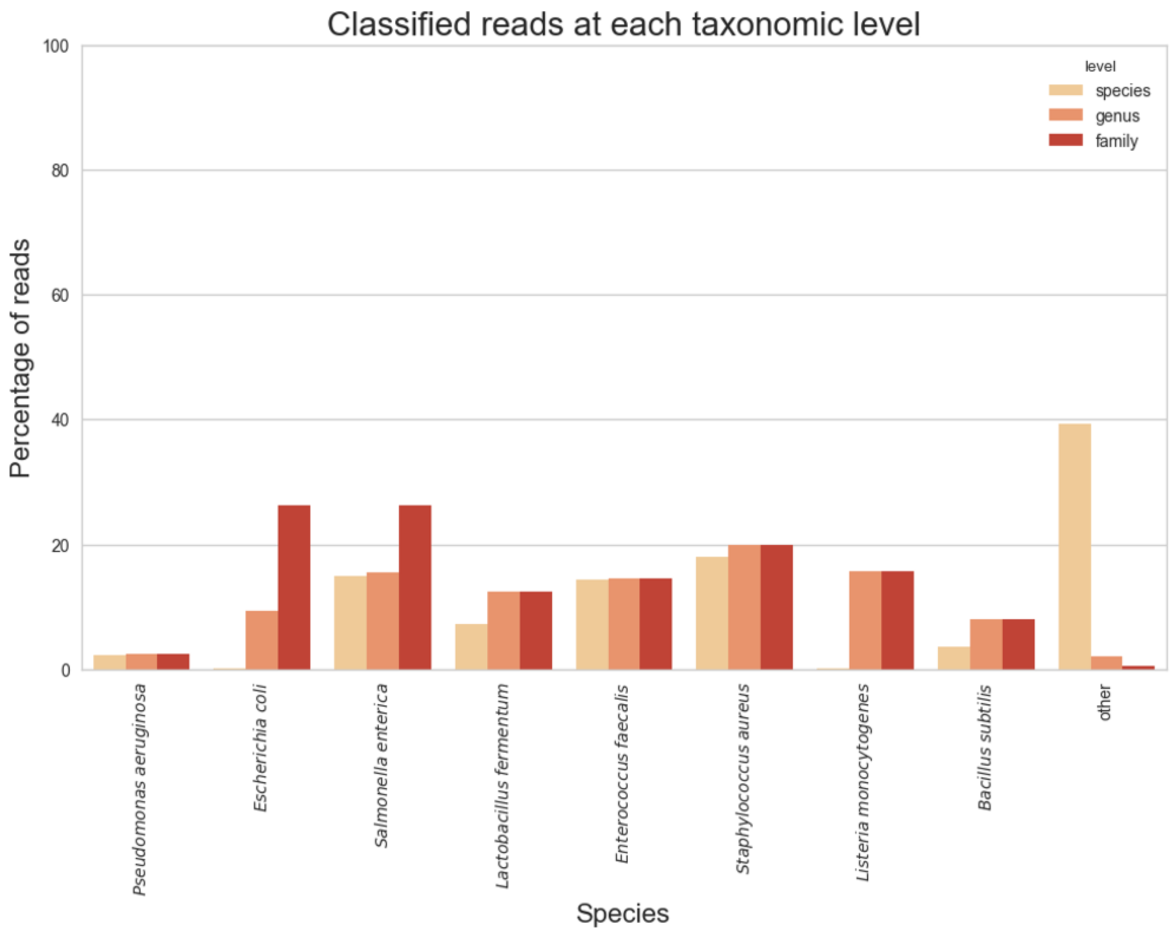

Figure S37. Overview of the percentages of reads that were correctly identified at the different taxonomic levels when using the GraphMap workflow and NCBI 16S database for the MinION data. All percentages were calculated against the number of classified reads at that level.

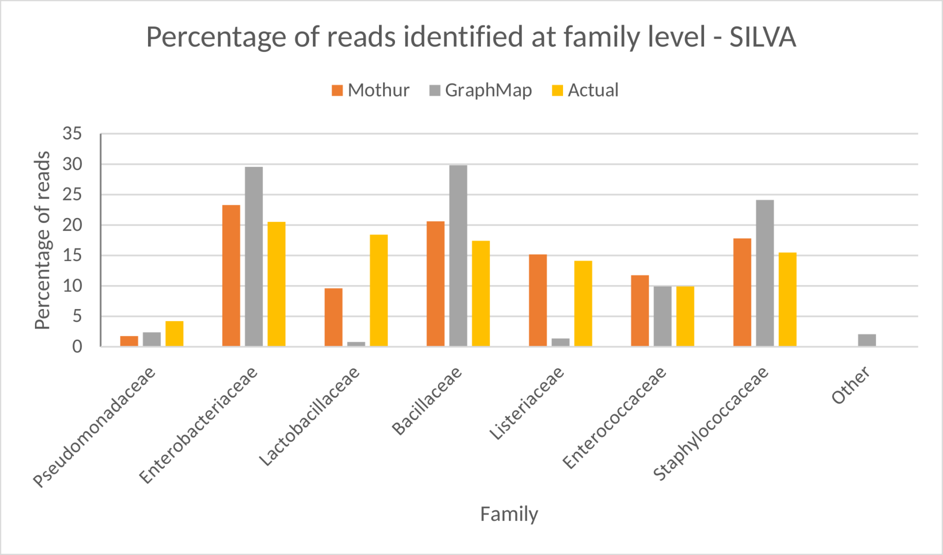

Figure S38. Overview of the percentages of reads that were correctly identified at the family level for the different families part of the mock community with the three different workflows and SILVA database for the MinION data. The "other" entry shows reads that were classified as a family not present in the mock community. The "Actual" bar shows the percentage of 16S rRNA that is present in the mock community for each species belonging that family.

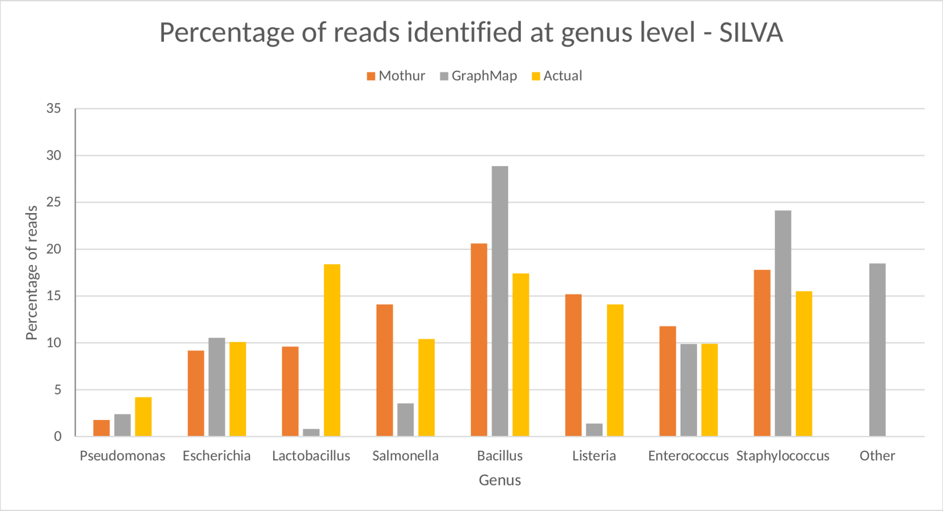

Figure S39. Overview of the percentages of reads that were correctly identified at the genus level for the different genera part of the mock community with the three different workflows and SILVA database for the MinION data. The "other" entry shows reads that were classified as a genus not present in the mock community. The "Actual" bar shows the percentage of 16S rRNA that is present in the mock community for each species belonging that genus.

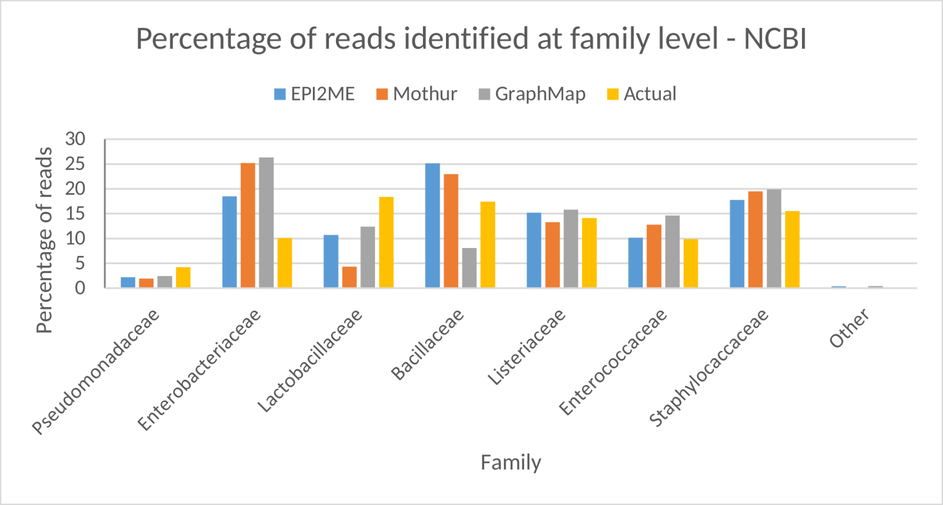

Figure S40. Overview of the percentages of reads that were correctly identified at the family level for the different families part of the mock community with the three different workflows and NCBI 16S database for the MinION data. The "other" entry shows reads that were classified as a family not present in the mock community. The "Actual" bar shows the percentage of 16S rRNA that is present in the mock community for each species belonging that family.

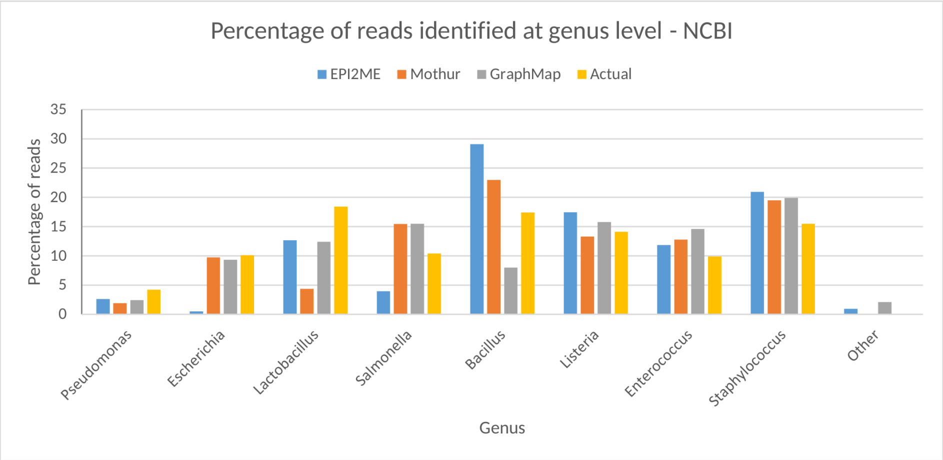

Figure S41. Overview of the percentages of reads that were correctly identified at the genus level for the different genera part of the mock community with the three different workflows and NCBI 16S database for the MinION data. The "other" entry shows reads that were classified as a genus not present in the mock community. The "Actual" bar shows the percentage of 16S rRNA that is present in the mock community for each species belonging that genus.

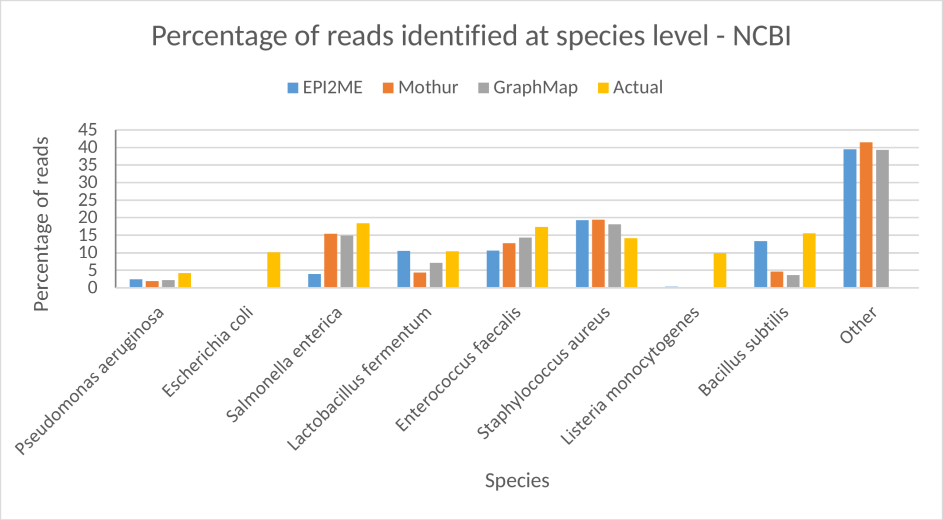

Figure S42. Overview of the percentages of reads that were correctly identified at the species level for the different species part of the mock community with the three different workflows and NCBI 16S database for the MinION data. The "other" entry shows reads that were classified as a species not present in the mock community. The "Actual" bar shows the percentage of 16S rRNA that is present in the mock community for each species.

1. Laurence, M.; Hatzis, C.; Brash, D.E. Common contaminants in next-generation sequencing that hinder discovery of low-abundance microbes. *PLoS ONE* **2014**, *9*, 1–8. doi:10.1371/journal.pone.0097876.
2. Salter, S.J.; Cox, M.J.; Turek, E.M.; Calus, S.T.; Cookson, W.O.; Moffatt, M.F.; Turner, P.; Parkhill, J.; Loman, N.J.; Walker, A.W. Reagent and laboratory contamination can critically impact sequence-based microbiome analyses. *BMC Biology* **2014**, *12*, 87. doi:10.1186/s12915-014-0087-z.
3. Weyrich, L.; Farrer, A.G.; Eisenhofer, R.; Arriola, L.A.; Young, J.; Selway, C.A.; Handsley-Davis, M.; Adler, C.; Breen, J.; Cooper, A. Laboratory contamination over time during low-biomass sample analysis [Preprint]. *bioRxiv* **2018**.
