# Supplementary figures and images for: Targeting the 16S rRNA Gene for Bacterial Identification in Complex Mixed Samples: Comparative Evaluation of Second (Illumina) and Third (Oxford Nanopore Technologies) Generation Sequencing Technologies
Source: Int J Mol Sci. 2019 Dec 31;21(1):298. doi: 10.3390/ijms21010298 (PMC6982111; doi:10.3390/ijms21010298)

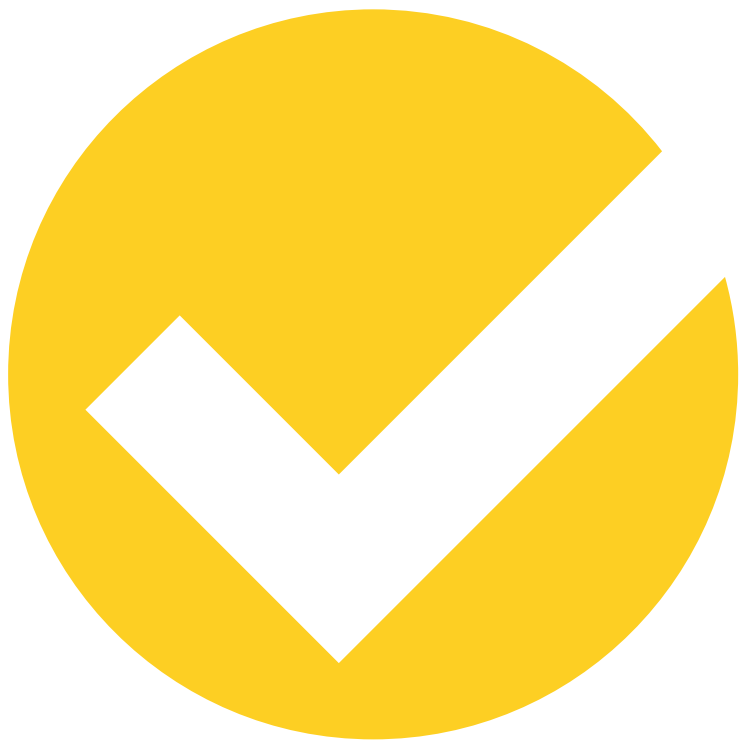

check for  
updates

Supplement: Supplementary file 1 [file ijms-21-00298-s001.zip › ijms-659682-proofreading si/Definitions/logo-updates.pdf]

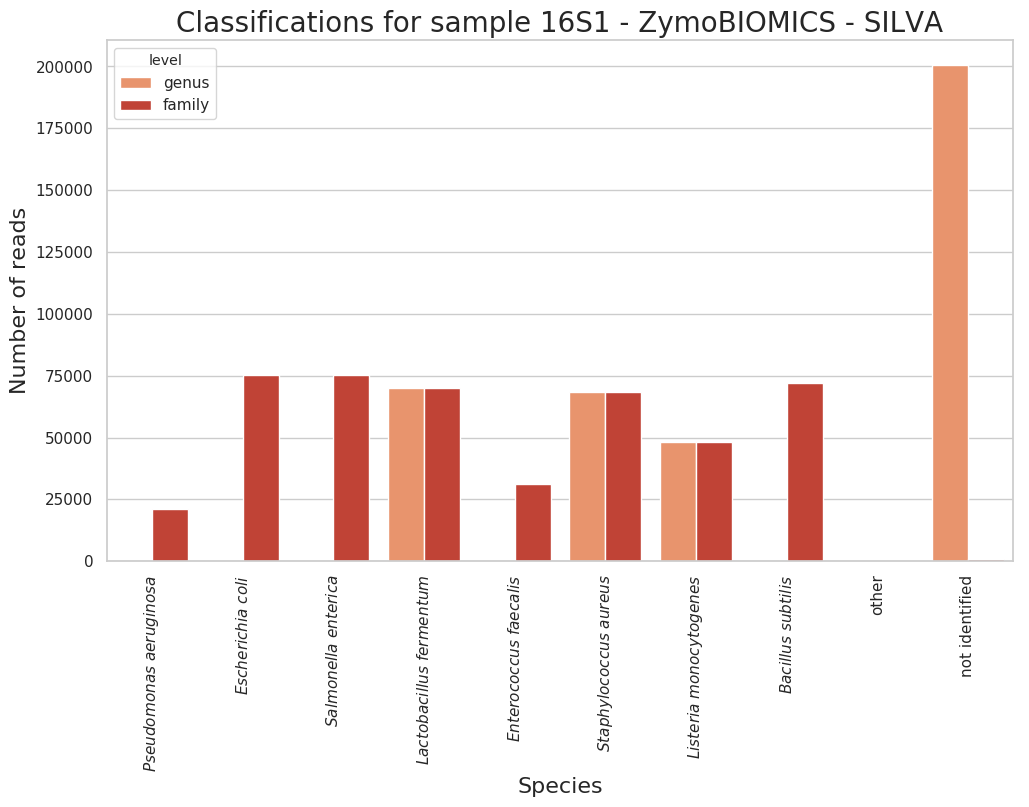

Supplement: Supplementary file 1 [file ijms-21-00298-s001.zip › ijms-659682-proofreading si/figures/16S1_mothur_silva_Zymo_Mock.png]

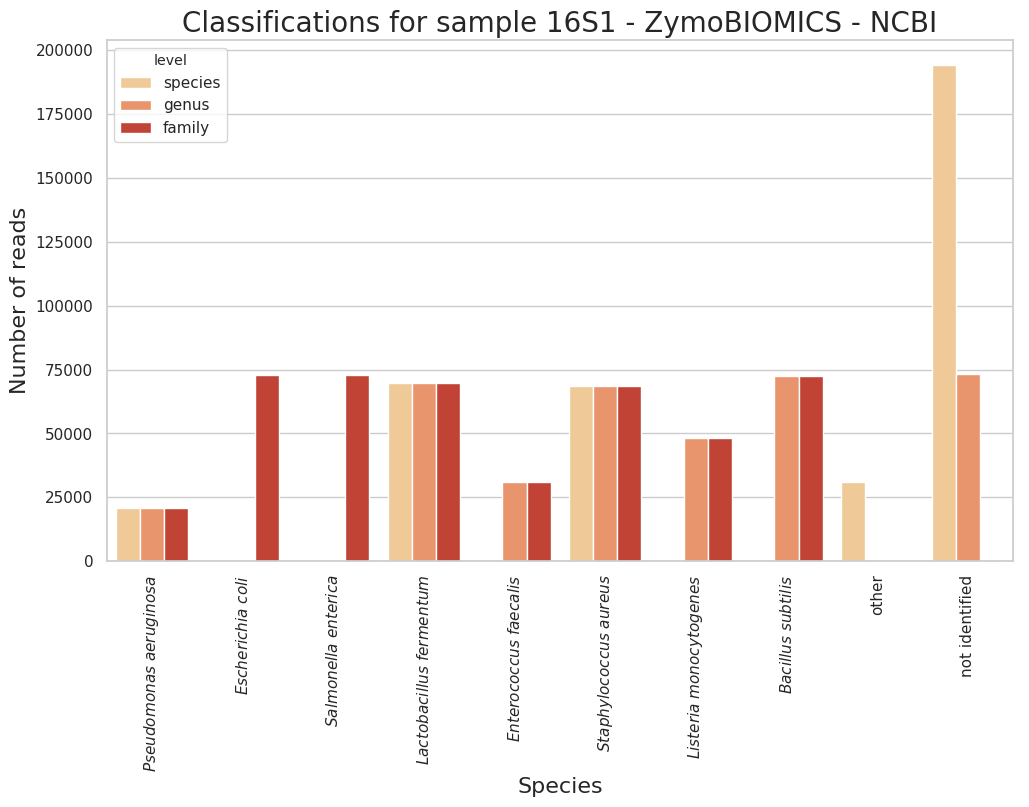

Supplement: Supplementary file 1 [file ijms-21-00298-s001.zip › ijms-659682-proofreading si/figures/16S1_ncbi_16s_Zymo_Mock.png]

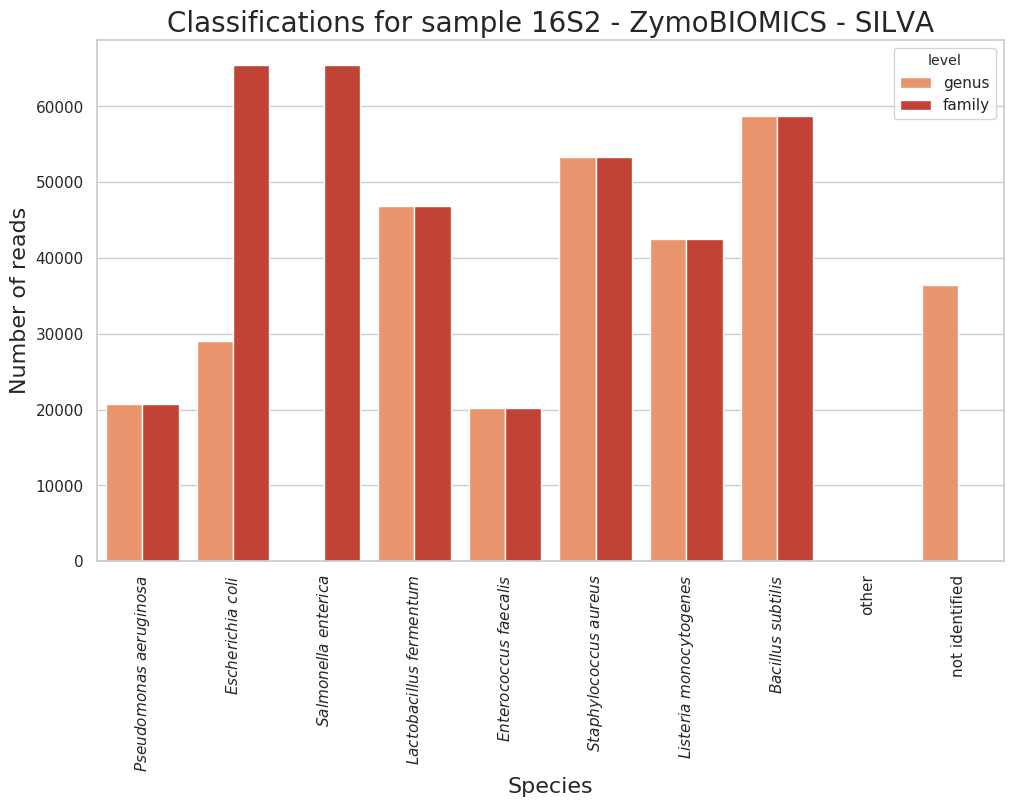

Supplement: Supplementary file 1 [file ijms-21-00298-s001.zip › ijms-659682-proofreading si/figures/16S2_mothur_silva_Zymo_Mock.png]

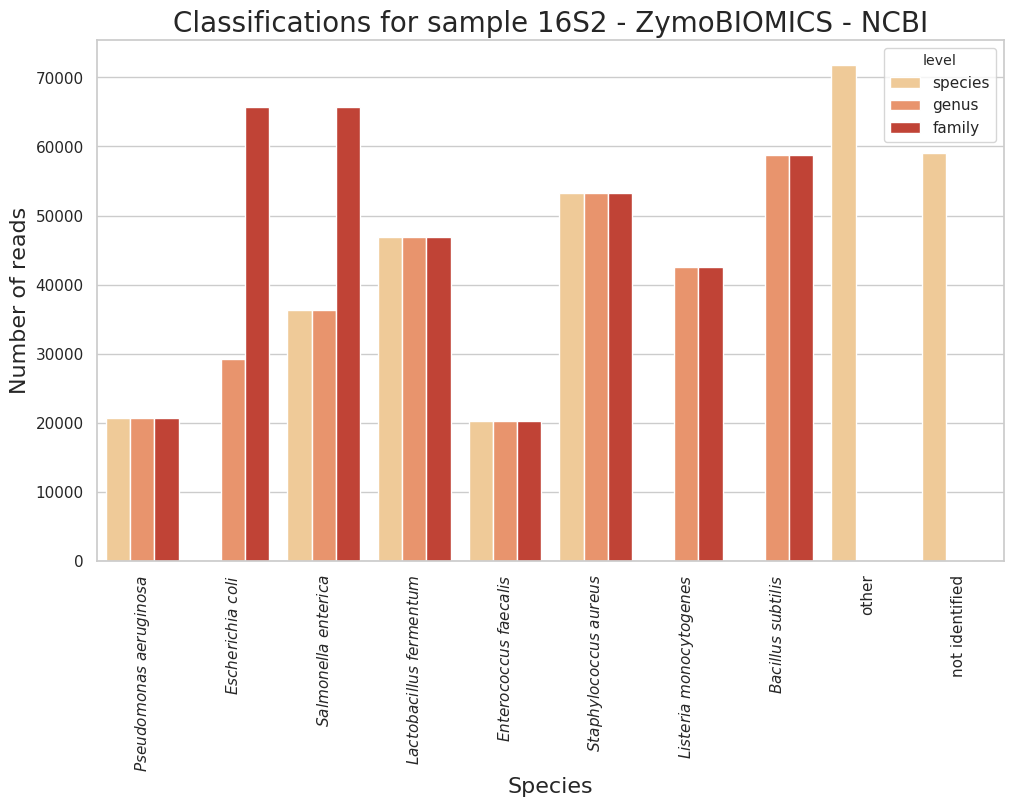

Supplement: Supplementary file 1 [file ijms-21-00298-s001.zip › ijms-659682-proofreading si/figures/16S2_ncbi_16s_Zymo_Mock.png]

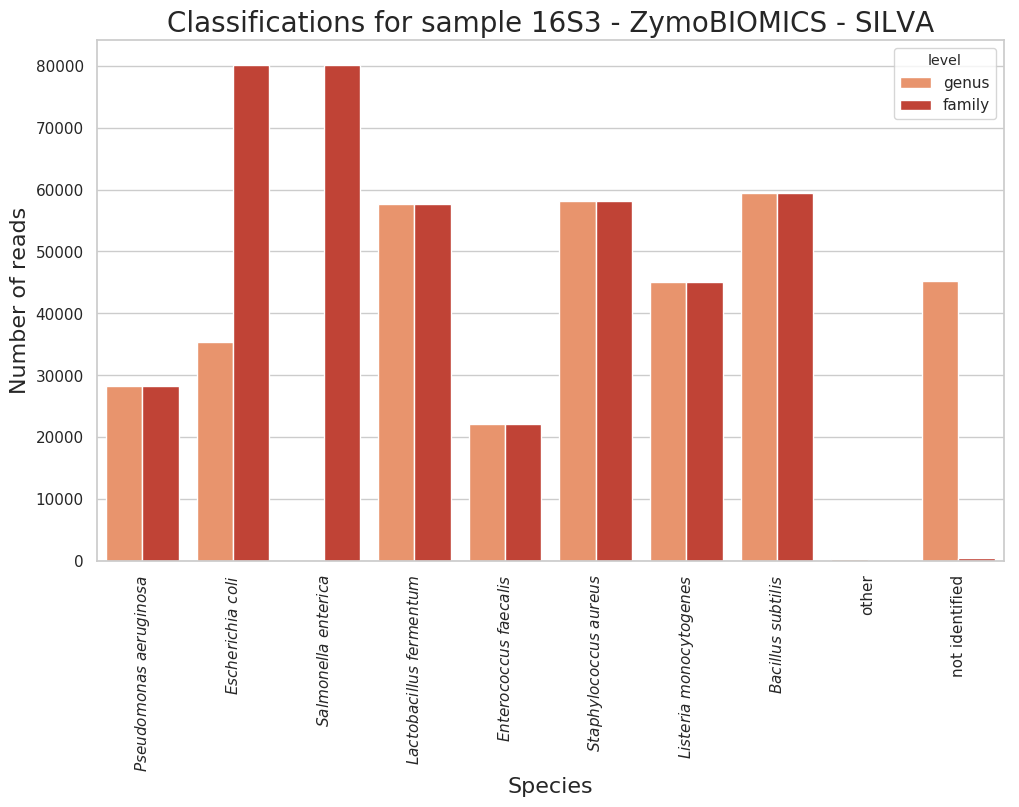

Supplement: Supplementary file 1 [file ijms-21-00298-s001.zip › ijms-659682-proofreading si/figures/16S3_mothur_silva_Zymo_Mock.png]

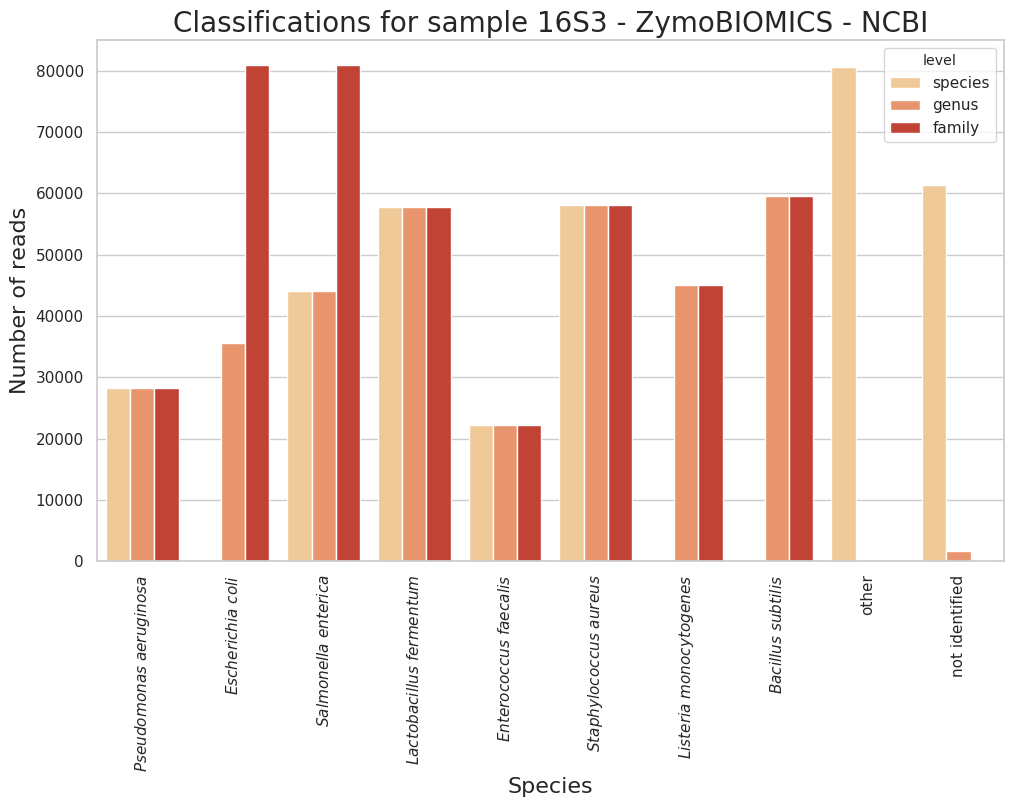

Supplement: Supplementary file 1 [file ijms-21-00298-s001.zip › ijms-659682-proofreading si/figures/16S3_ncbi_16s_Zymo_Mock.png]

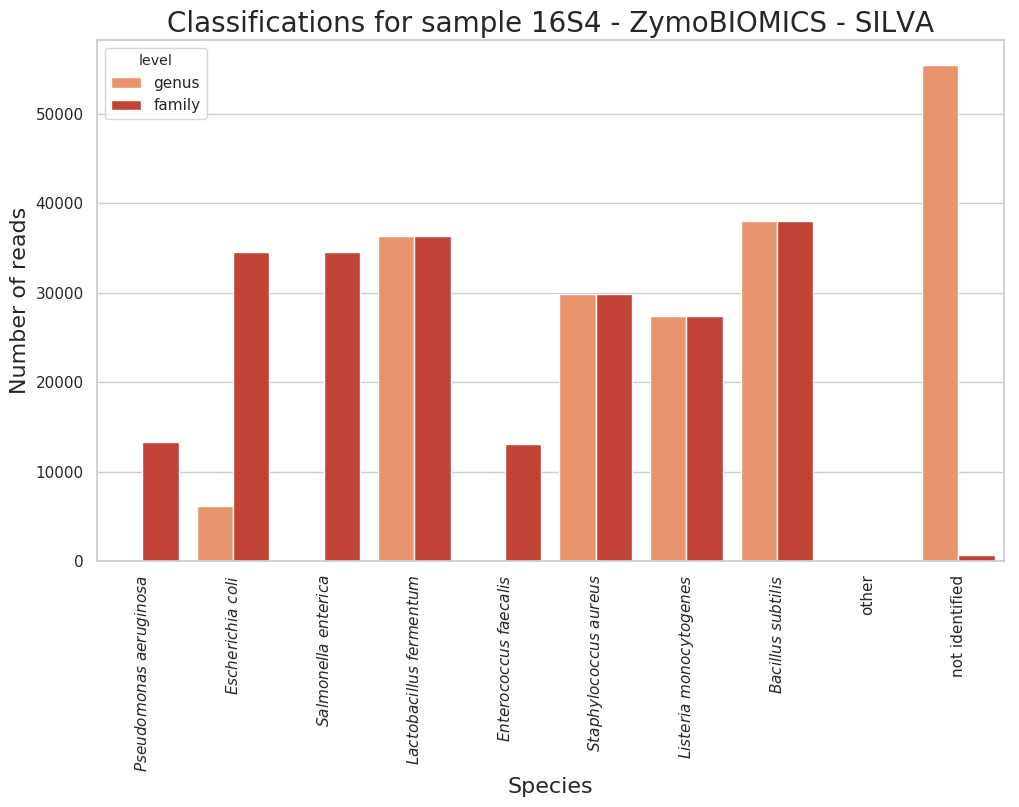

Supplement: Supplementary file 1 [file ijms-21-00298-s001.zip › ijms-659682-proofreading si/figures/16S4_mothur_silva_Zymo_Mock.png]

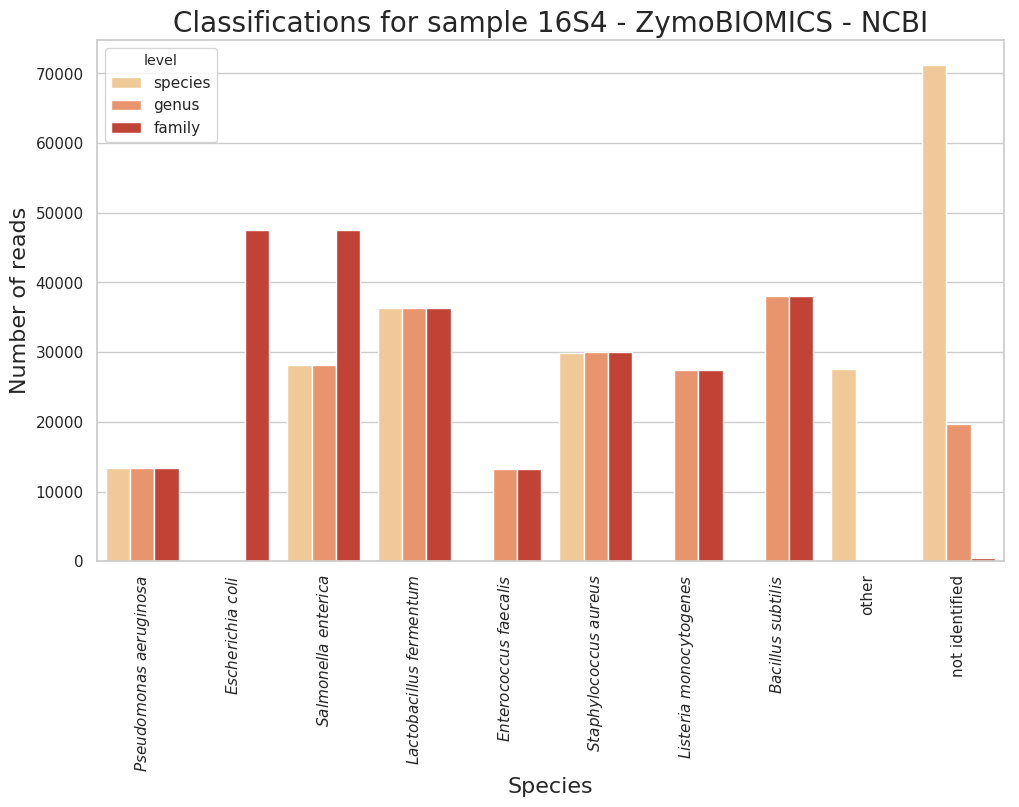

Supplement: Supplementary file 1 [file ijms-21-00298-s001.zip › ijms-659682-proofreading si/figures/16S4_ncbi_16s_Zymo_Mock.png]

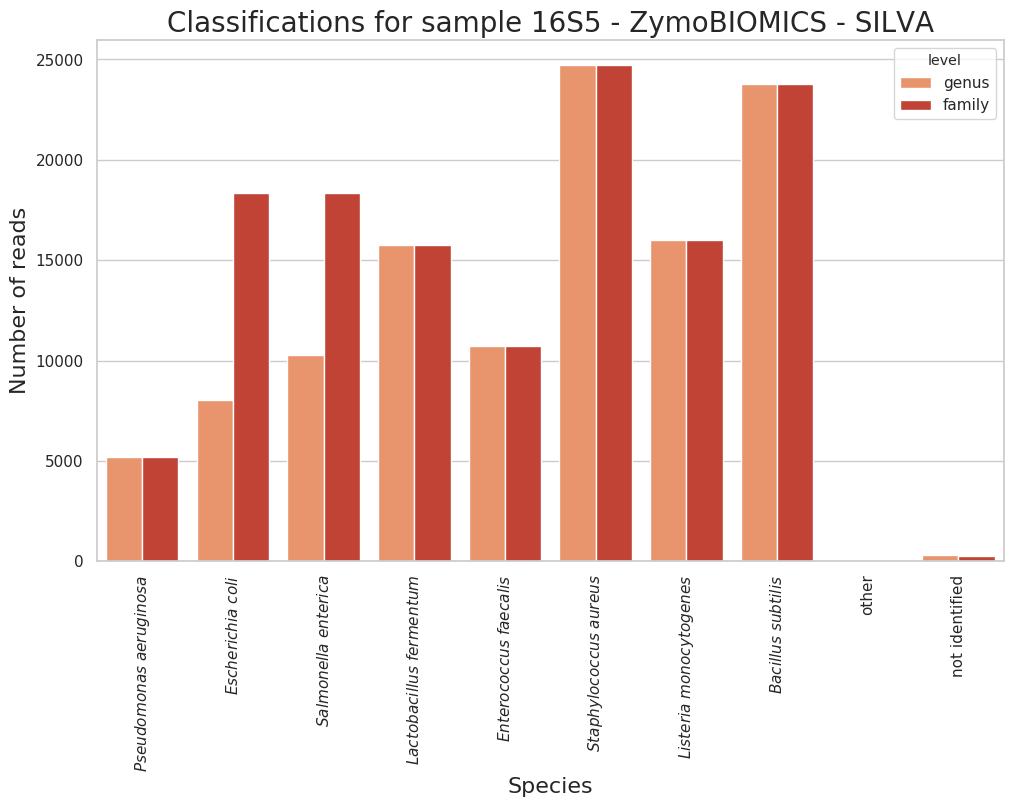

Supplement: Supplementary file 1 [file ijms-21-00298-s001.zip › ijms-659682-proofreading si/figures/16S5_mothur_silva_Zymo_Mock.png]

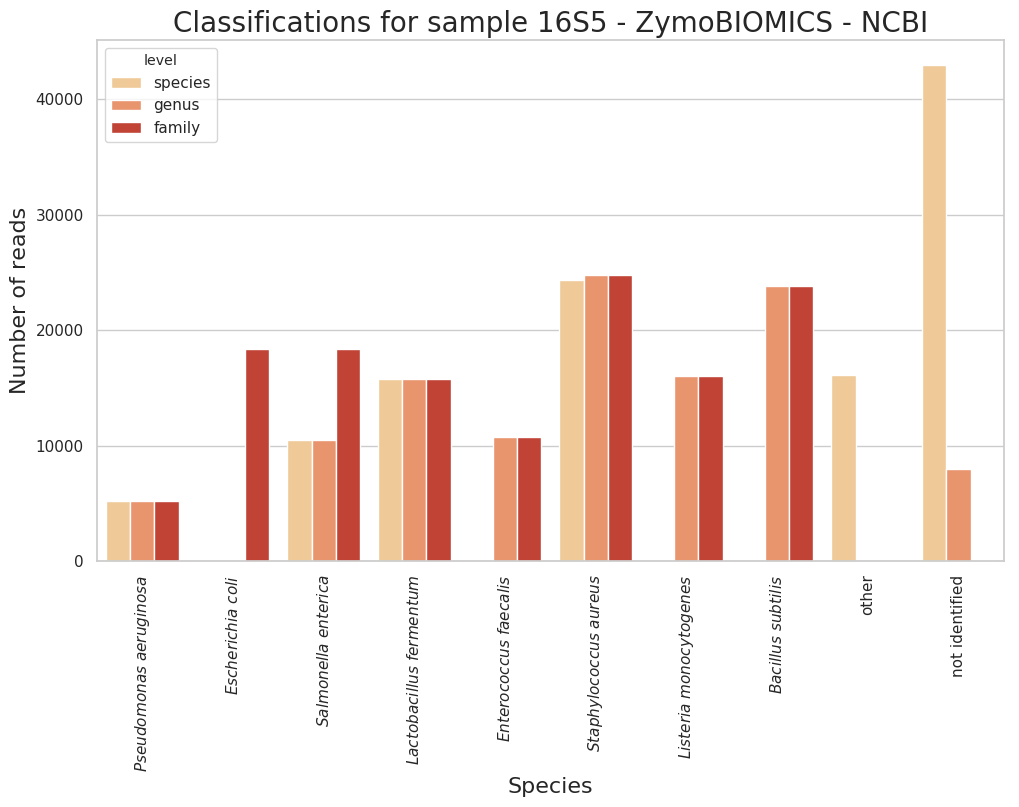

Supplement: Supplementary file 1 [file ijms-21-00298-s001.zip › ijms-659682-proofreading si/figures/16S5_ncbi_16s_Zymo_Mock.png]

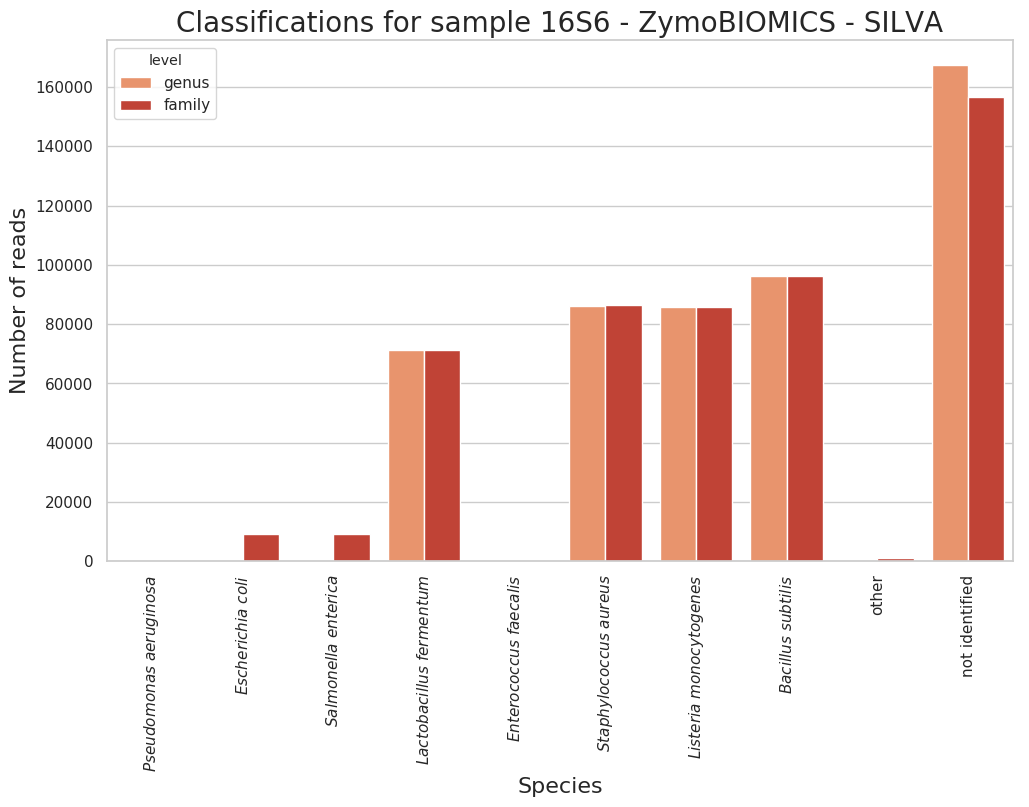

Supplement: Supplementary file 1 [file ijms-21-00298-s001.zip › ijms-659682-proofreading si/figures/16S6_mothur_silva_Zymo_Mock.png]

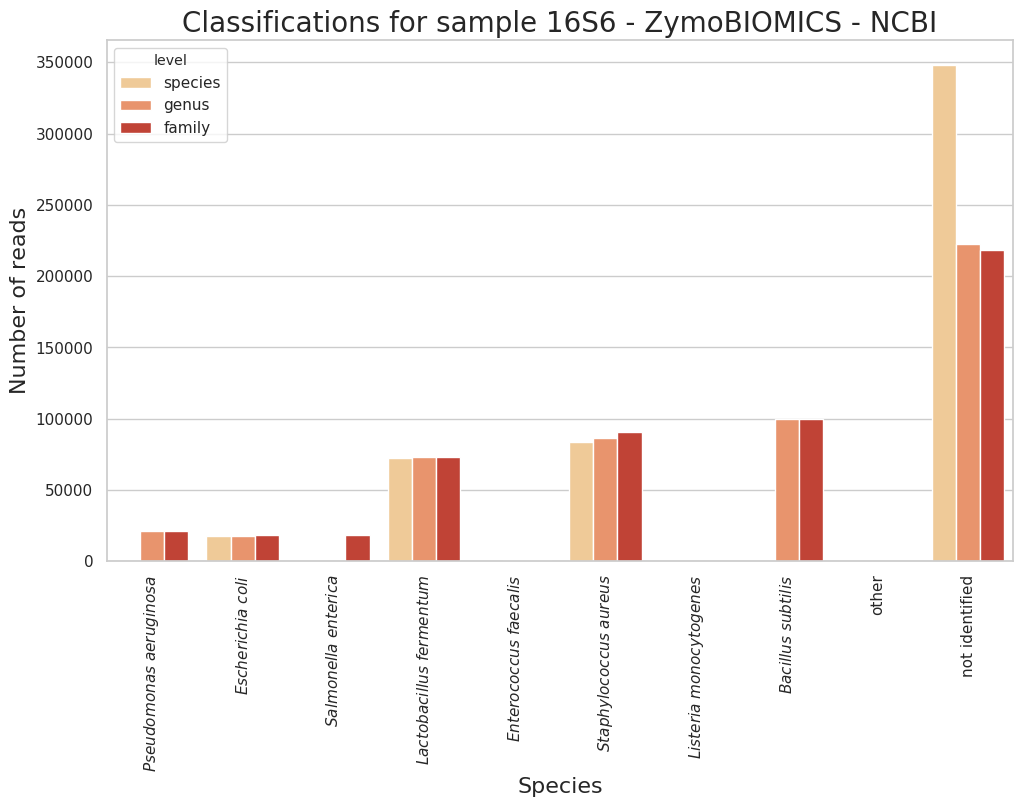

Supplement: Supplementary file 1 [file ijms-21-00298-s001.zip › ijms-659682-proofreading si/figures/16S6_ncbi_16s_Zymo_Mock.png]

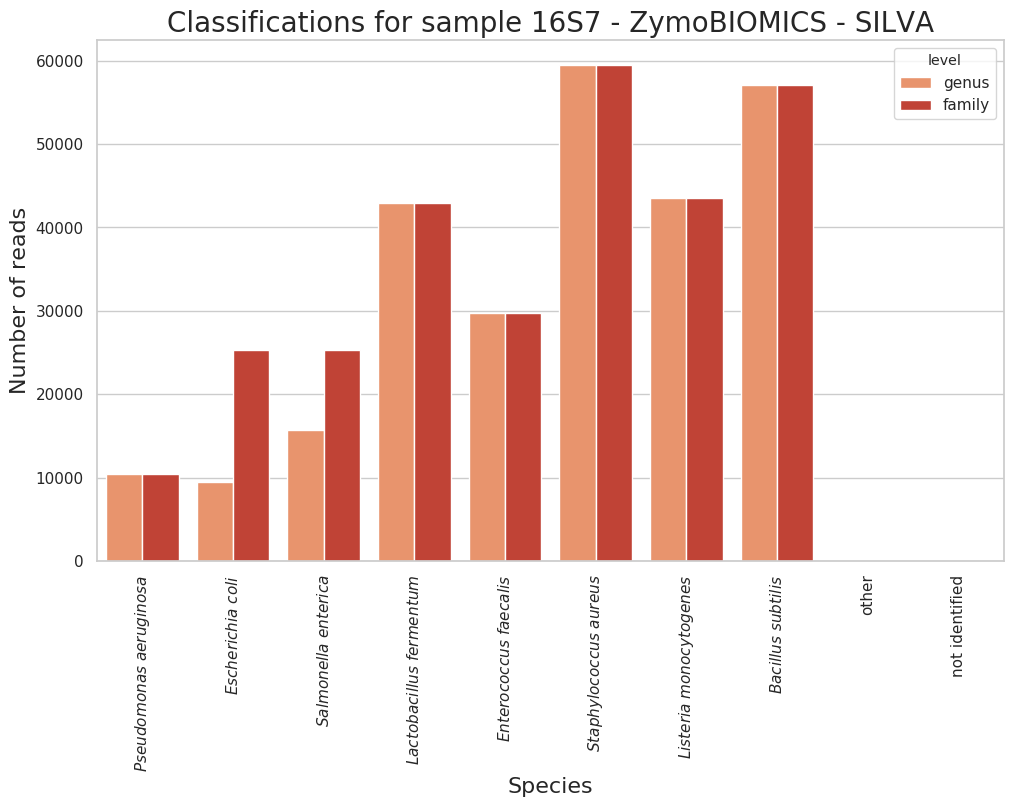

Supplement: Supplementary file 1 [file ijms-21-00298-s001.zip › ijms-659682-proofreading si/figures/16S7_mothur_silva_Zymo_Mock.png]

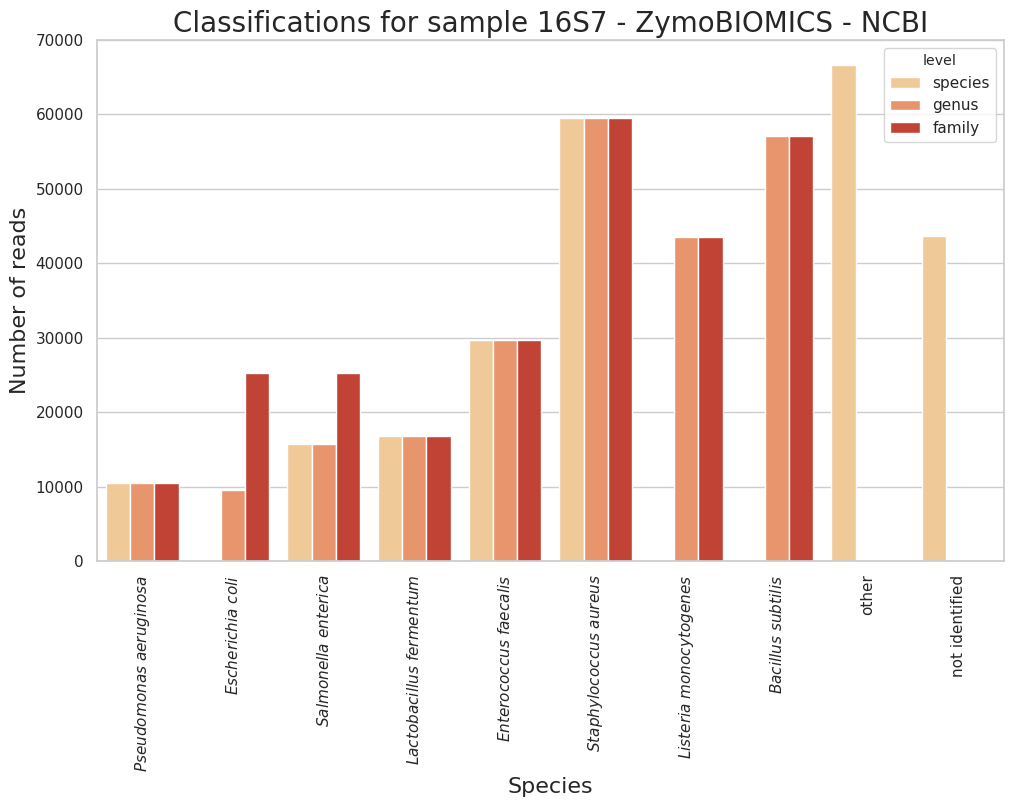

Supplement: Supplementary file 1 [file ijms-21-00298-s001.zip › ijms-659682-proofreading si/figures/16S7_ncbi_16s_Zymo_Mock.png]

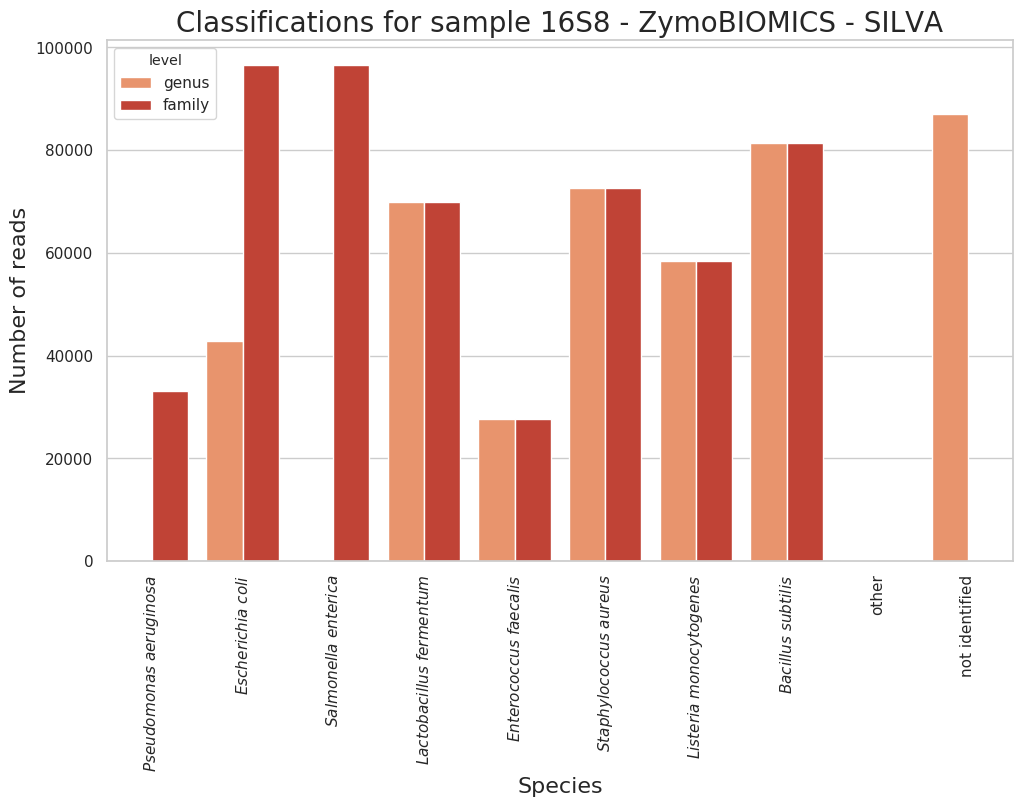

Supplement: Supplementary file 1 [file ijms-21-00298-s001.zip › ijms-659682-proofreading si/figures/16S8_mothur_silva_Zymo_Mock.png]

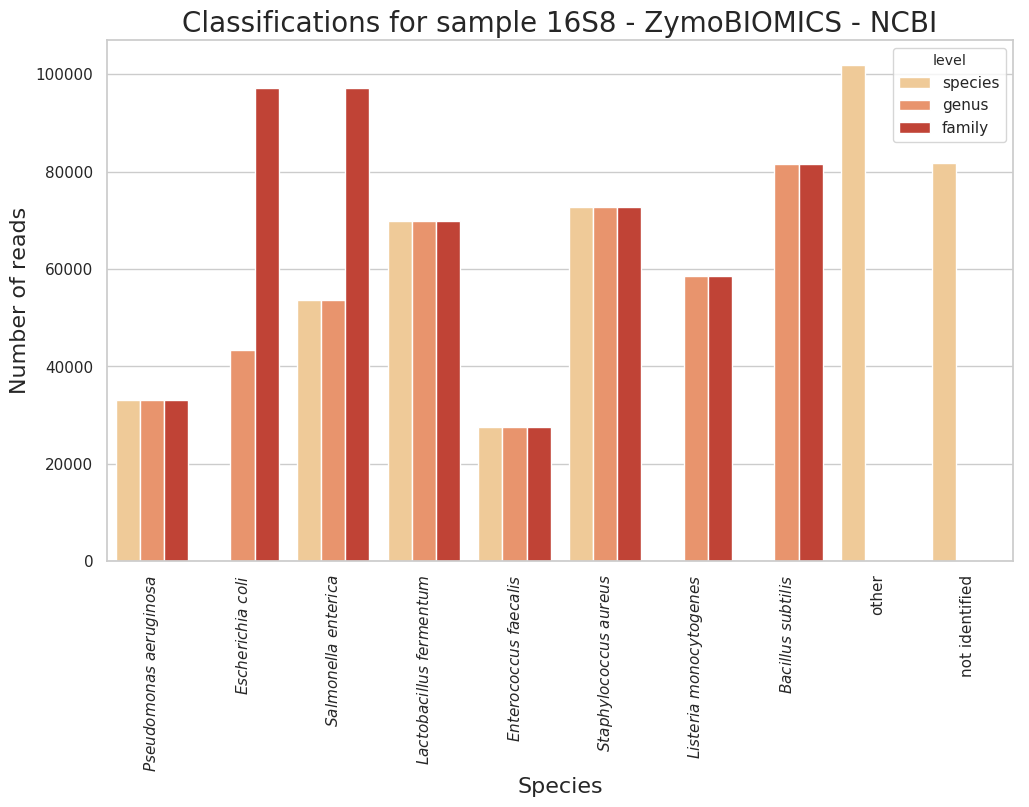

Supplement: Supplementary file 1 [file ijms-21-00298-s001.zip › ijms-659682-proofreading si/figures/16S8_ncbi_16s_Zymo_Mock.png]

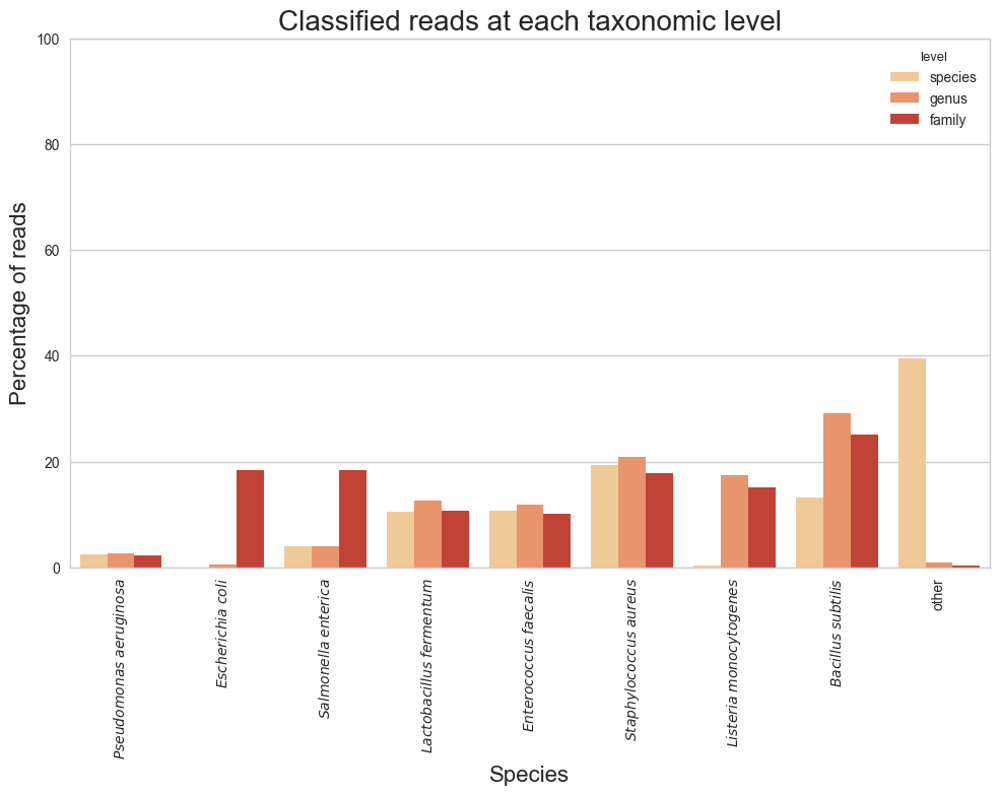

Supplement: Supplementary file 1 [file ijms-21-00298-s001.zip › ijms-659682-proofreading si/figures/epi2me_combined_zymo_ncbi.png]

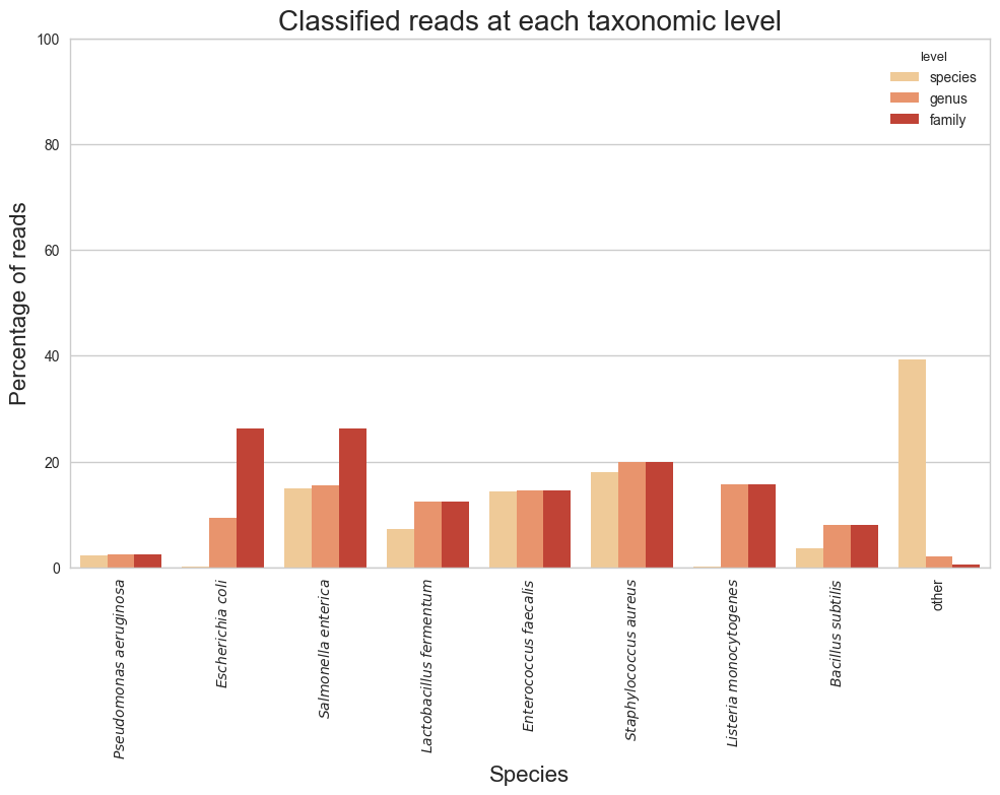

Supplement: Supplementary file 1 [file ijms-21-00298-s001.zip › ijms-659682-proofreading si/figures/graphmap_combined_Zymo_NCBI.png]

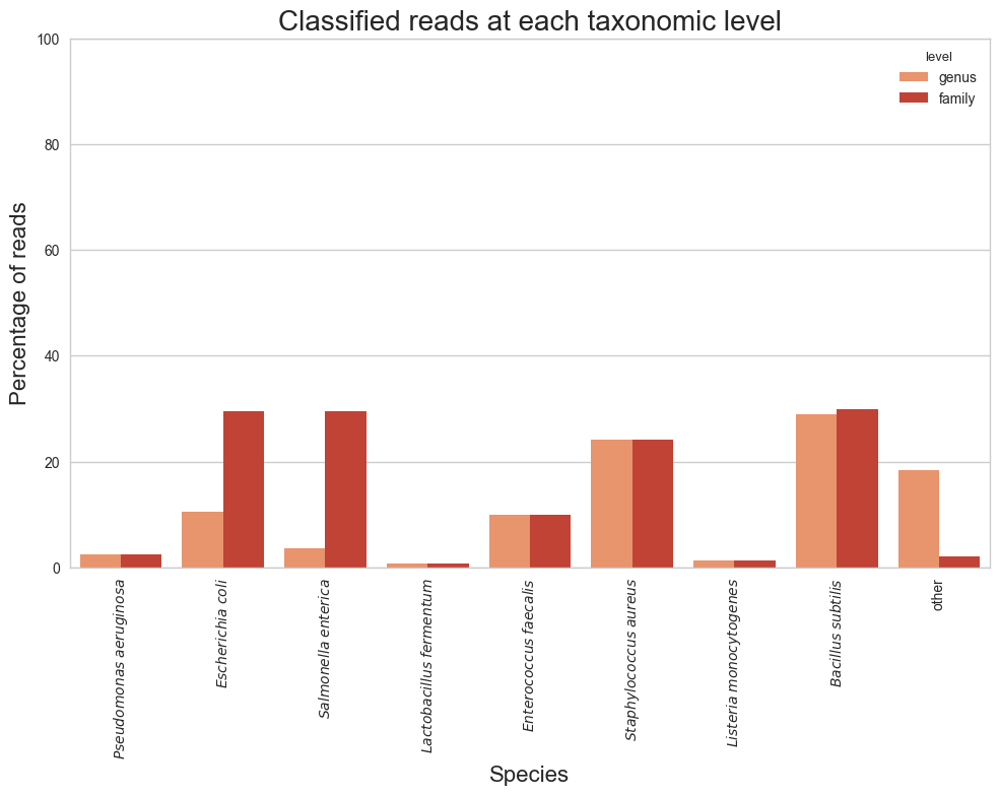

Supplement: Supplementary file 1 [file ijms-21-00298-s001.zip › ijms-659682-proofreading si/figures/graphmap_combined_Zymo_SILVA.png]

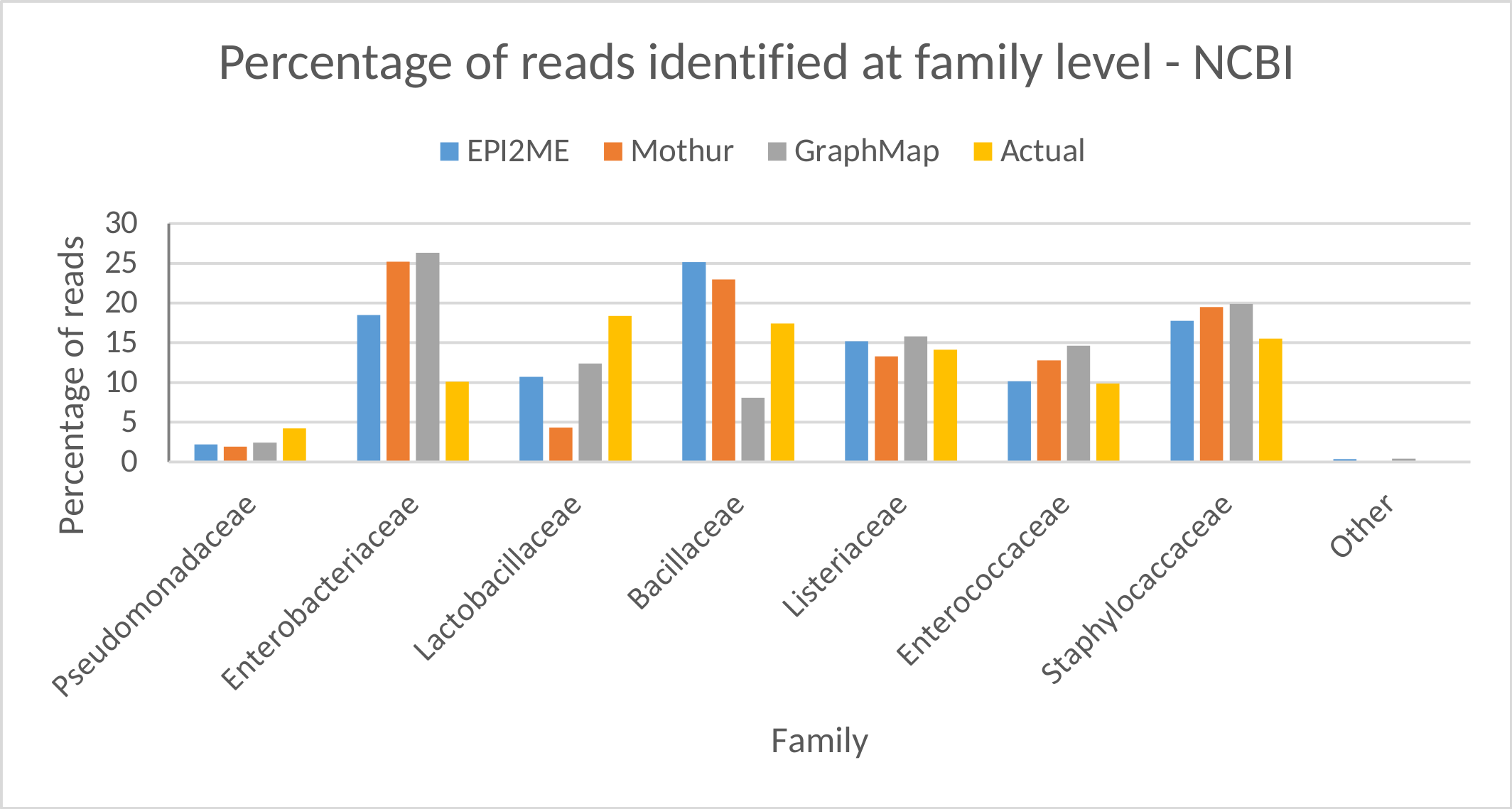

Supplement: Supplementary file 1 [file ijms-21-00298-s001.zip › ijms-659682-proofreading si/figures/minion_overview_ncbi_family.png]

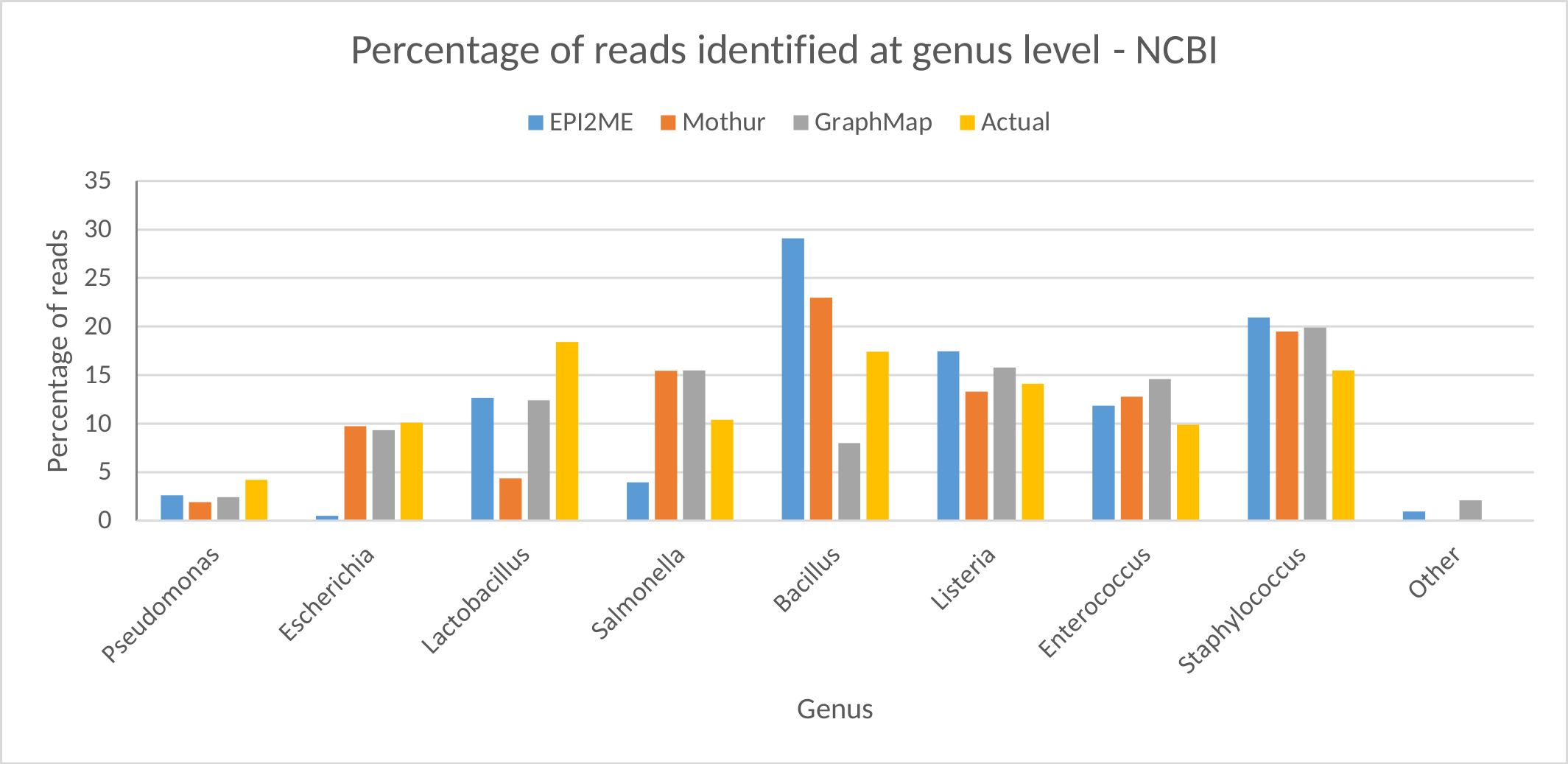

Supplement: Supplementary file 1 [file ijms-21-00298-s001.zip › ijms-659682-proofreading si/figures/minion_overview_ncbi_genus.png]

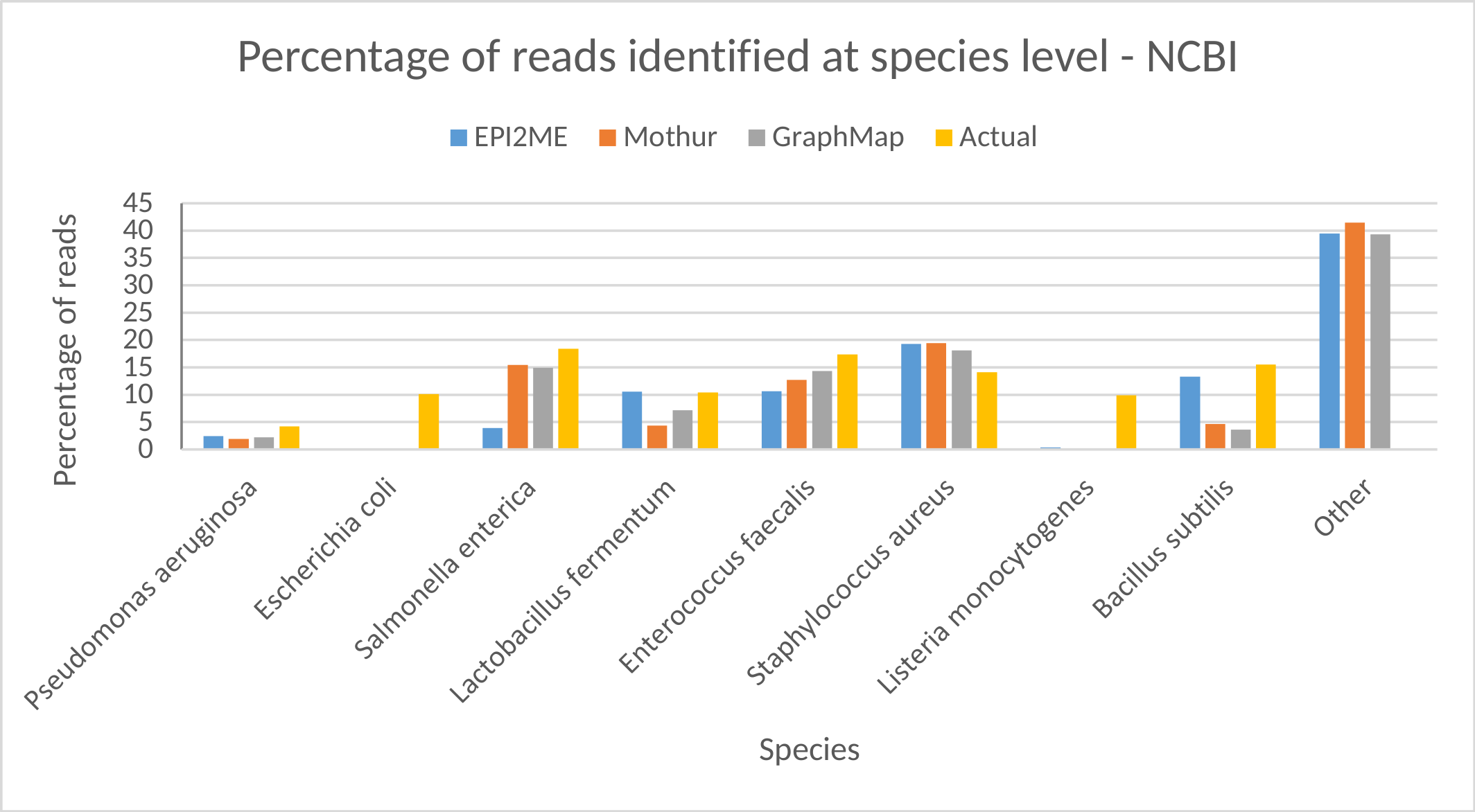

Supplement: Supplementary file 1 [file ijms-21-00298-s001.zip › ijms-659682-proofreading si/figures/minion_overview_ncbi_species.png]

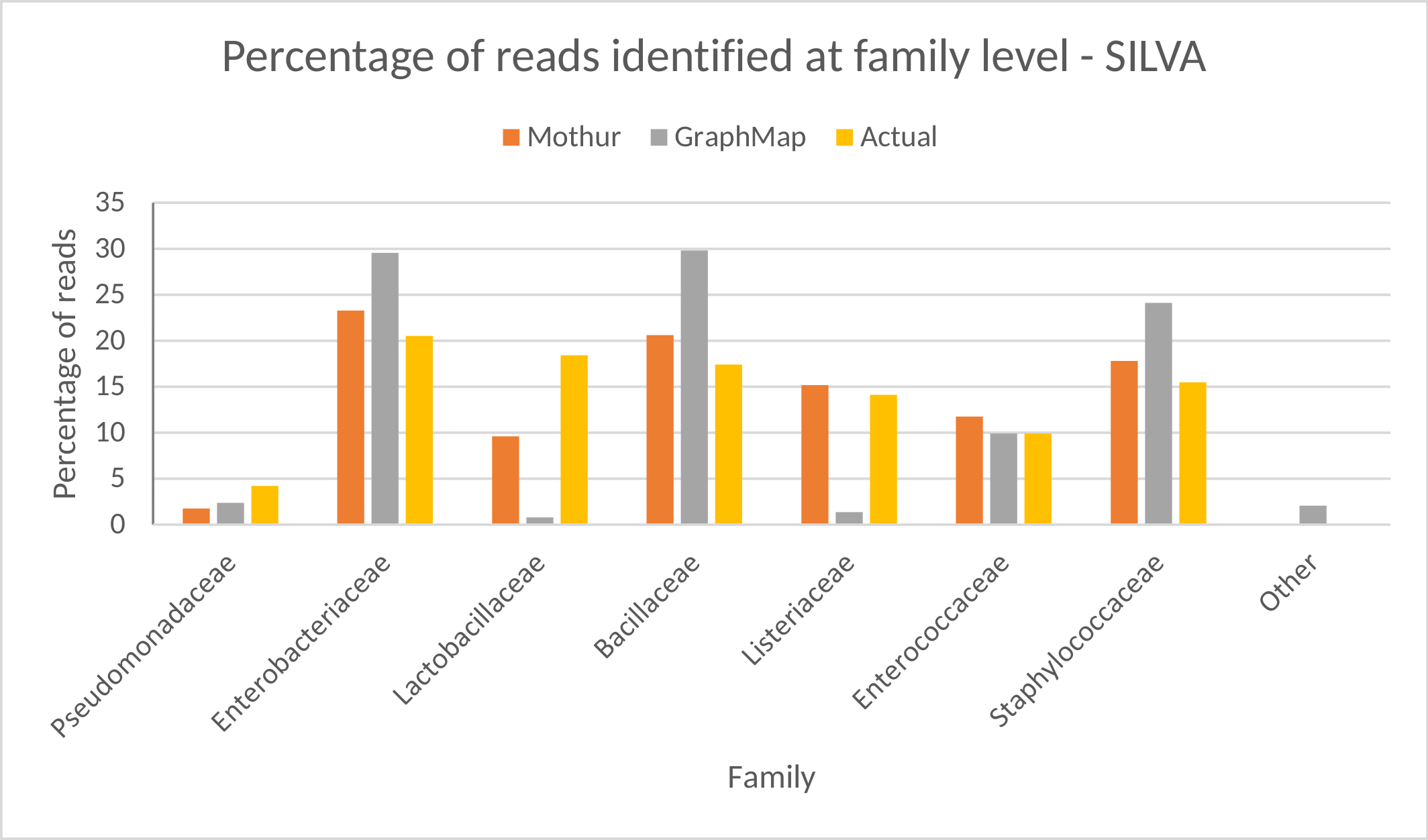

Supplement: Supplementary file 1 [file ijms-21-00298-s001.zip › ijms-659682-proofreading si/figures/minion_overview_silva_family.png]

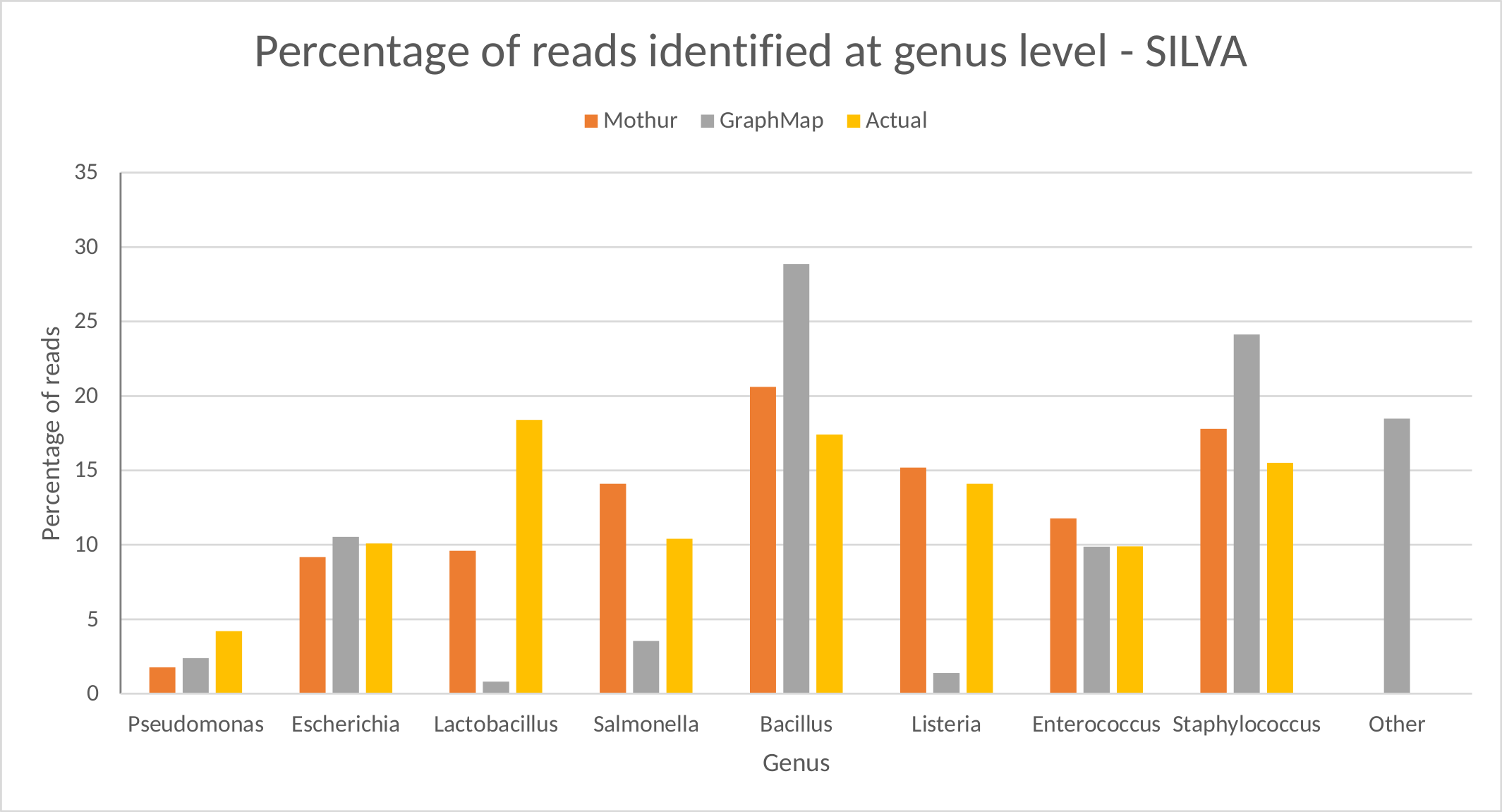

Supplement: Supplementary file 1 [file ijms-21-00298-s001.zip › ijms-659682-proofreading si/figures/minion_overview_silva_genus.png]

**A**

### Correctly classified reads - SILVA

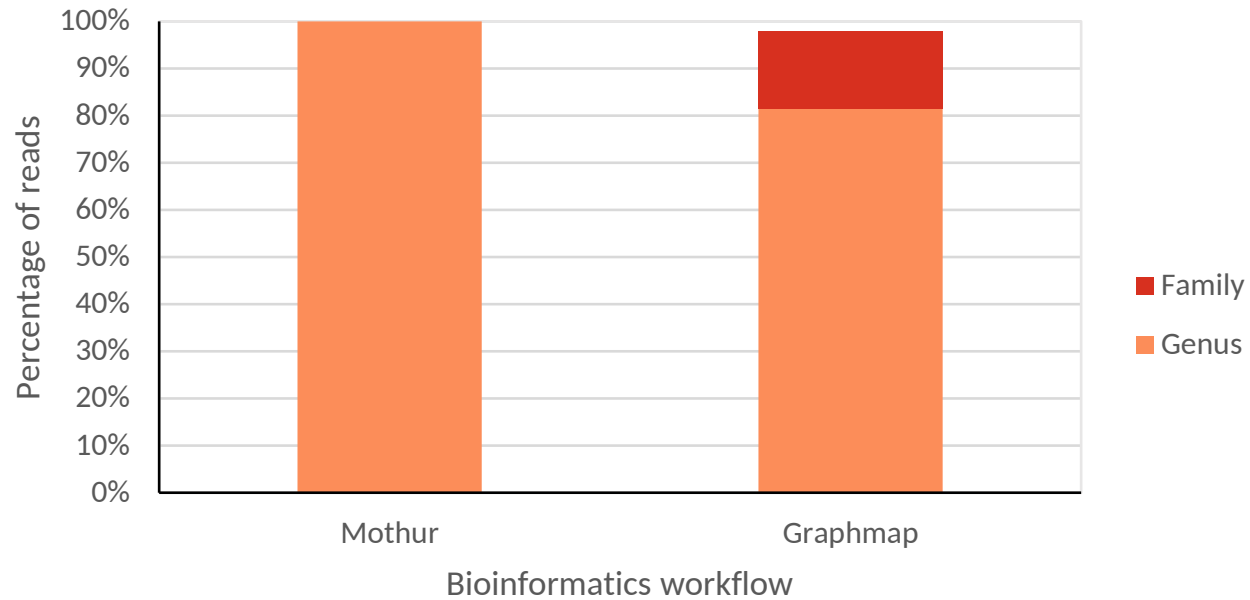**B**

### Correctly classified reads - NCBI 16S

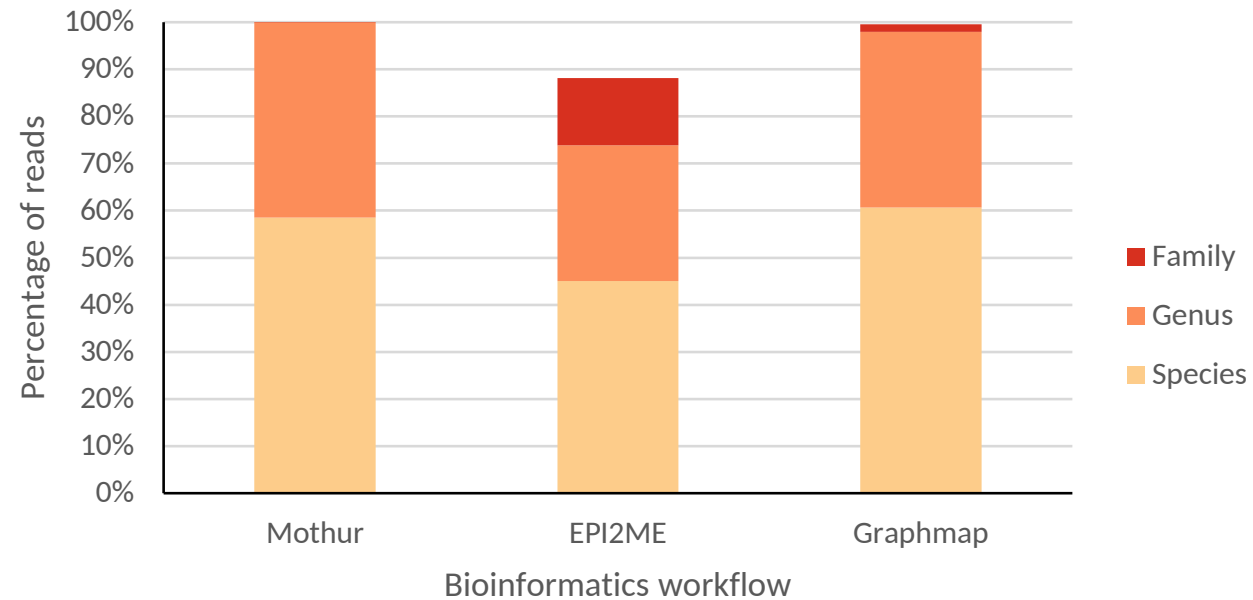

Supplement: Supplementary file 1 [file ijms-21-00298-s001.zip › ijms-659682-proofreading si/figures/minion_reads_reordered.pdf]

A

Correctly classified reads - SILVA

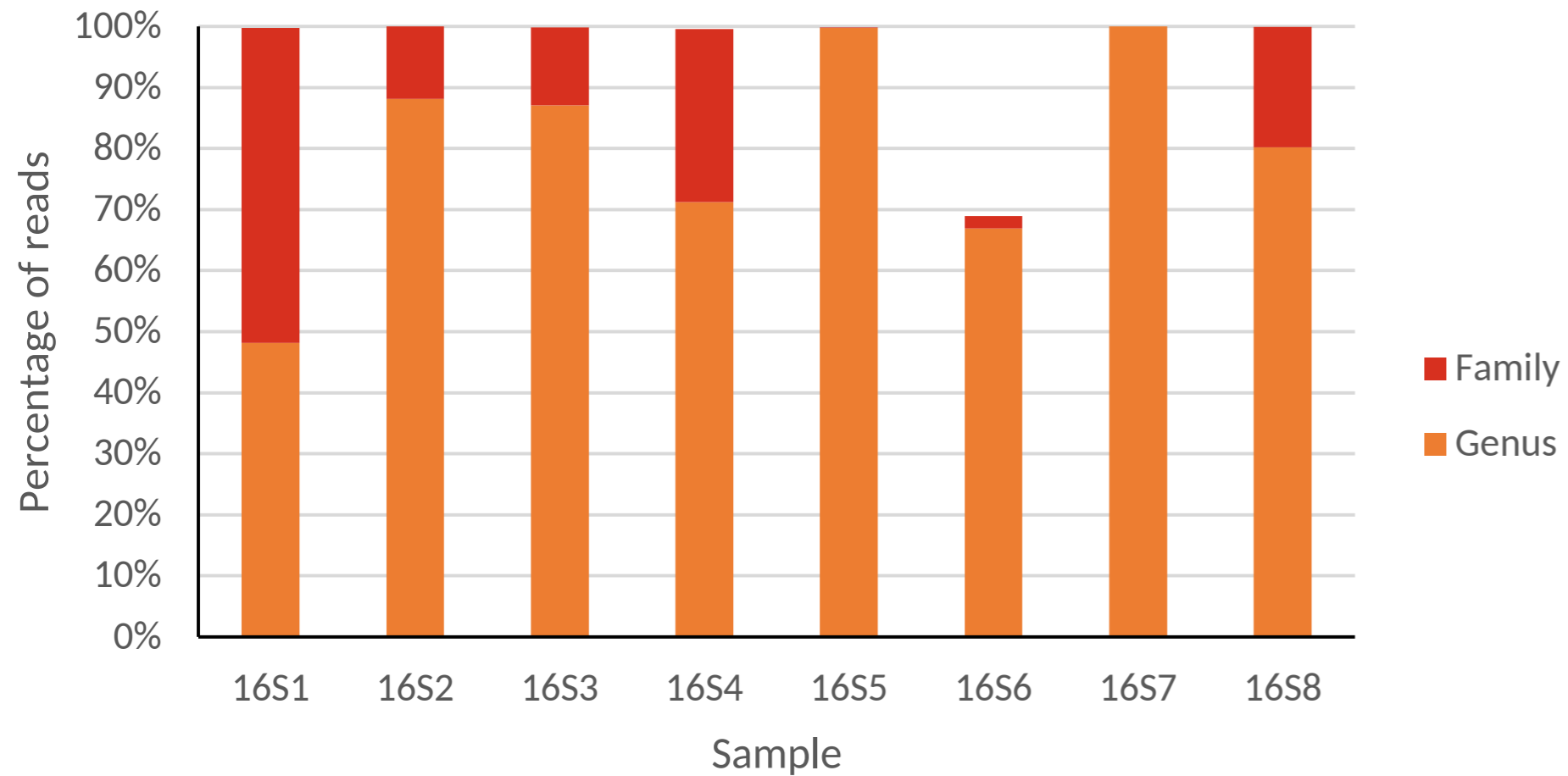

B

Correctly classified reads - NCBI 16S

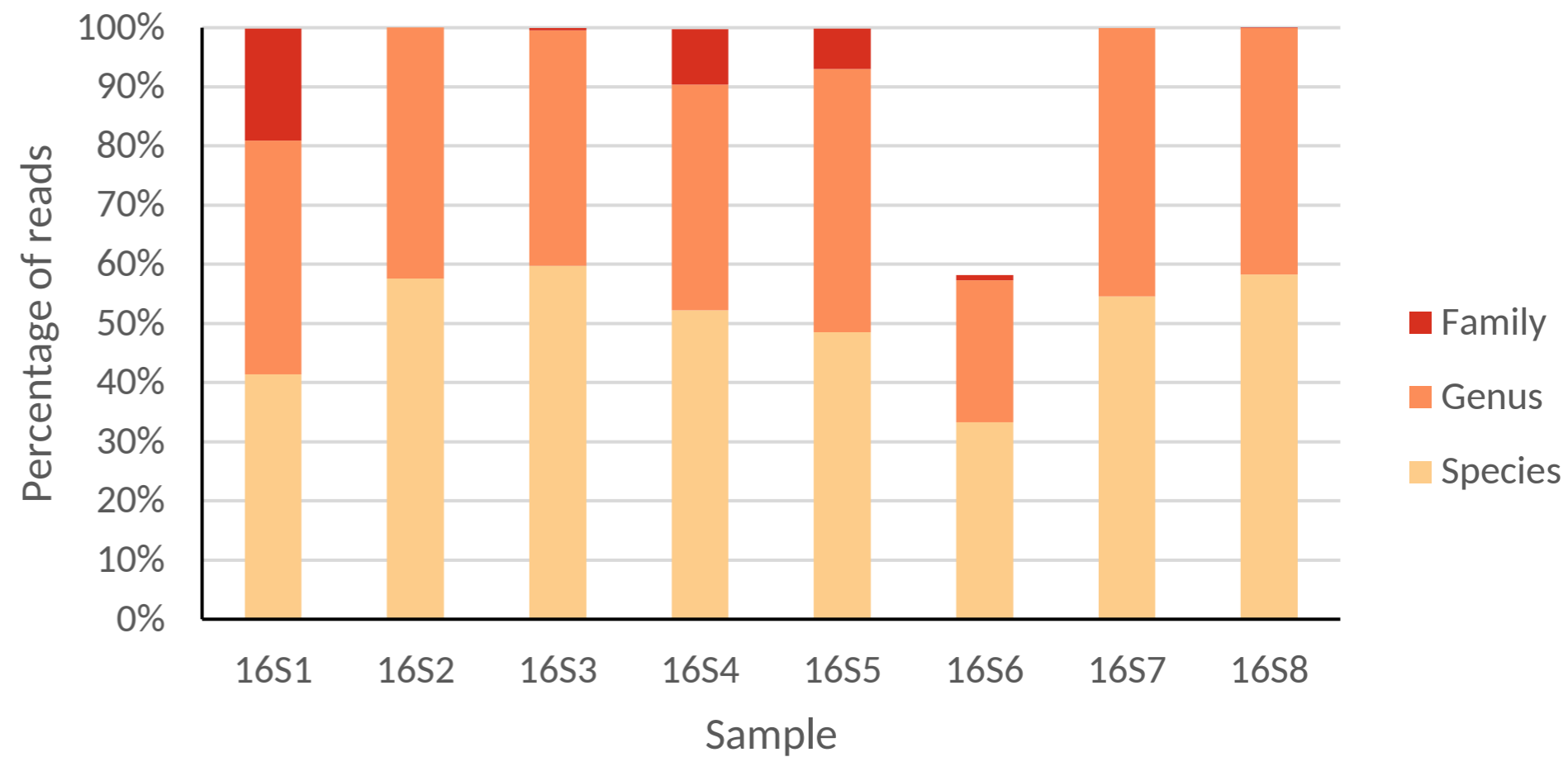

Supplement: Supplementary file 1 [file ijms-21-00298-s001.zip › ijms-659682-proofreading si/figures/miseq_reads.pdf]

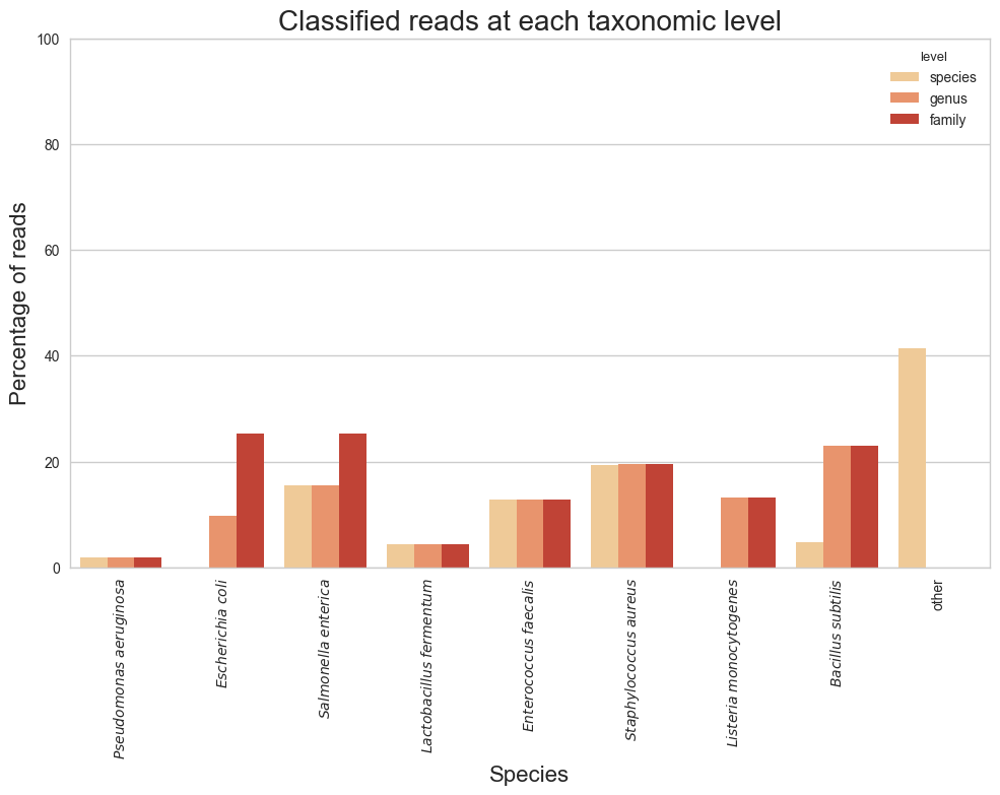

Supplement: Supplementary file 1 [file ijms-21-00298-s001.zip › ijms-659682-proofreading si/figures/mothur_combined_zymo_ncbi.png]

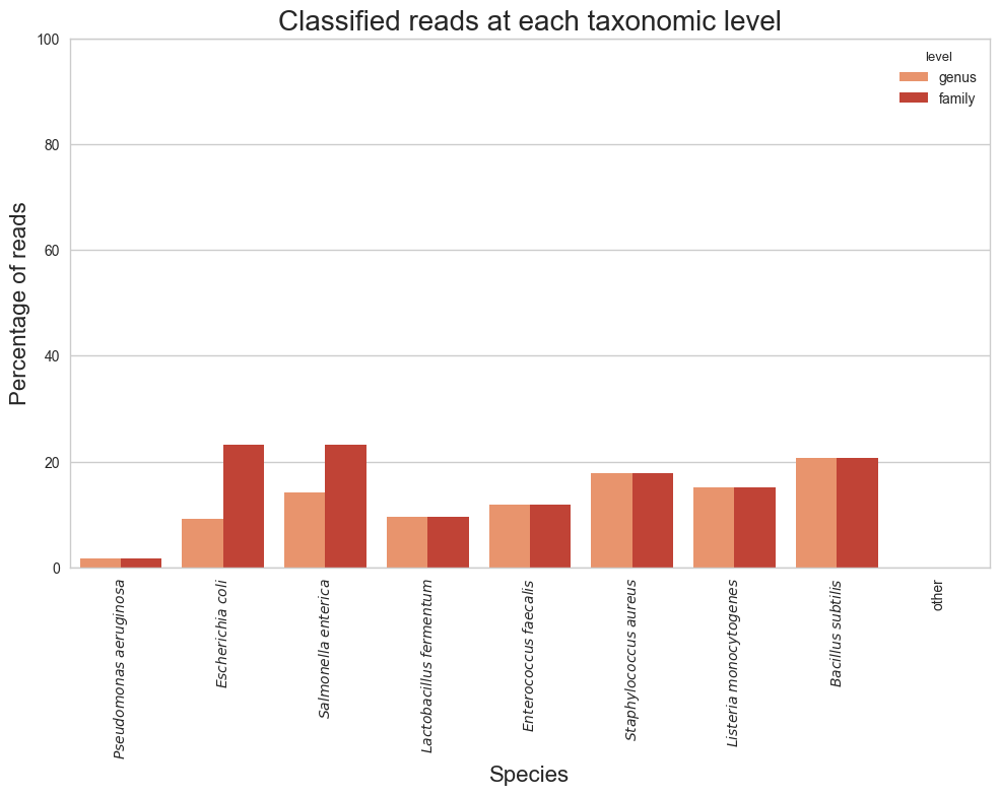

Supplement: Supplementary file 1 [file ijms-21-00298-s001.zip › ijms-659682-proofreading si/figures/mothur_combined_zymo_silva.png]

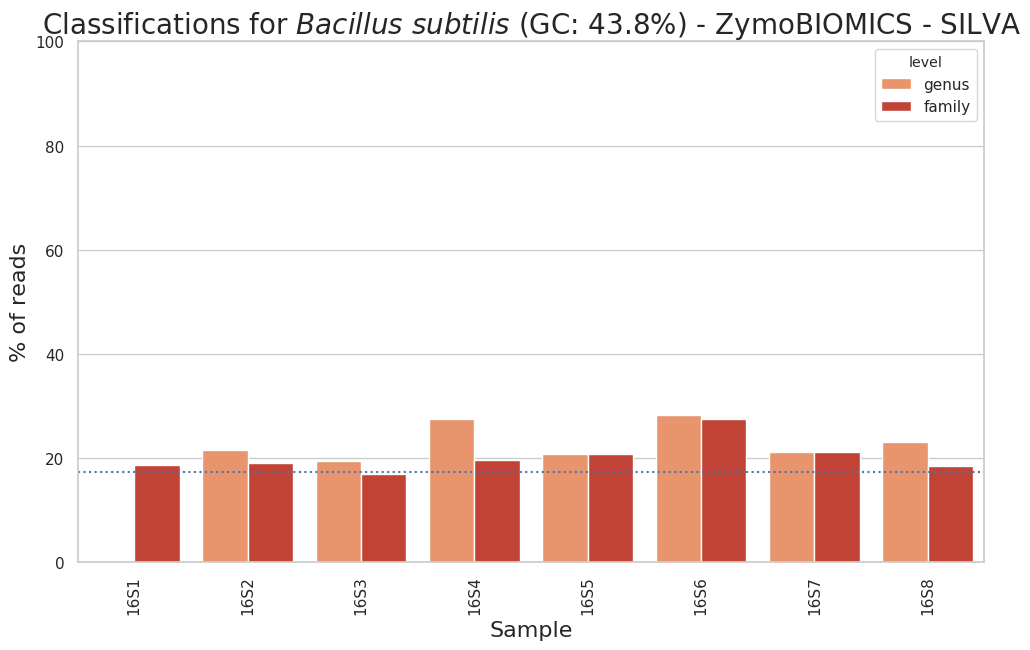

Supplement: Supplementary file 1 [file ijms-21-00298-s001.zip › ijms-659682-proofreading si/figures/Zymo_Mock_Bacillus_mothur_silva.png]

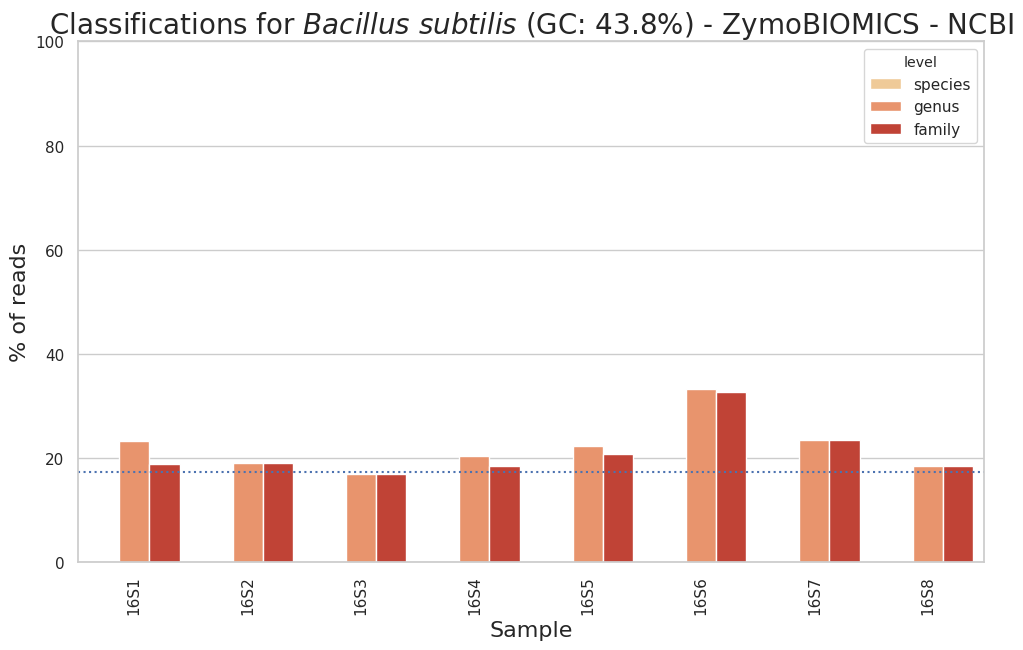

Supplement: Supplementary file 1 [file ijms-21-00298-s001.zip › ijms-659682-proofreading si/figures/Zymo_Mock_Bacillus_ncbi_16s.png]

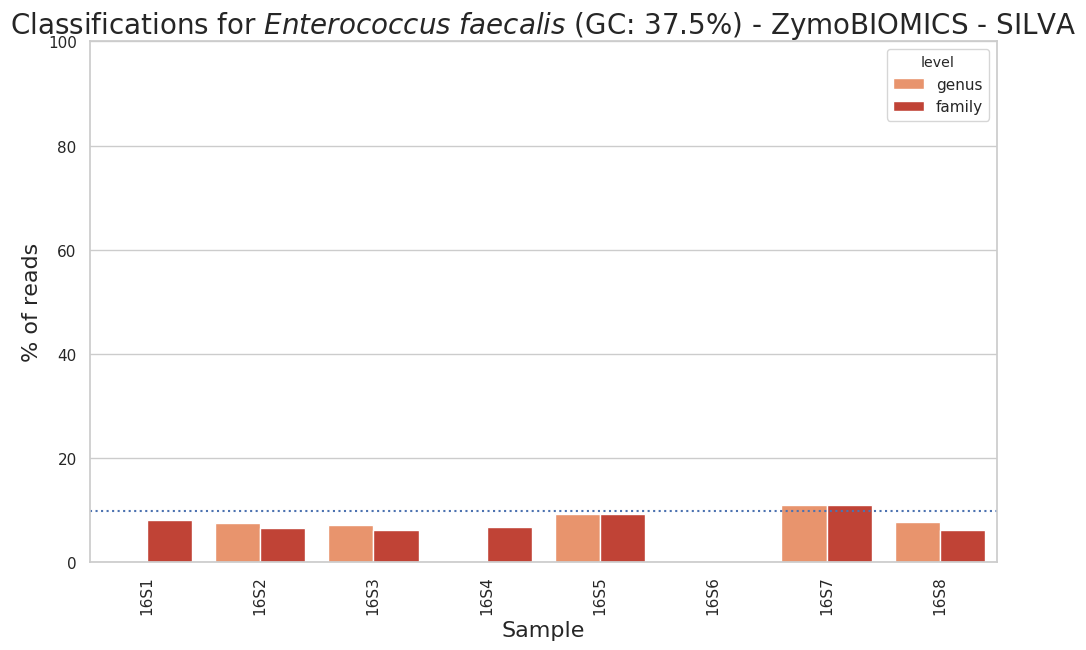

Supplement: Supplementary file 1 [file ijms-21-00298-s001.zip › ijms-659682-proofreading si/figures/Zymo_Mock_Enterococcus_mothur_silva.png]

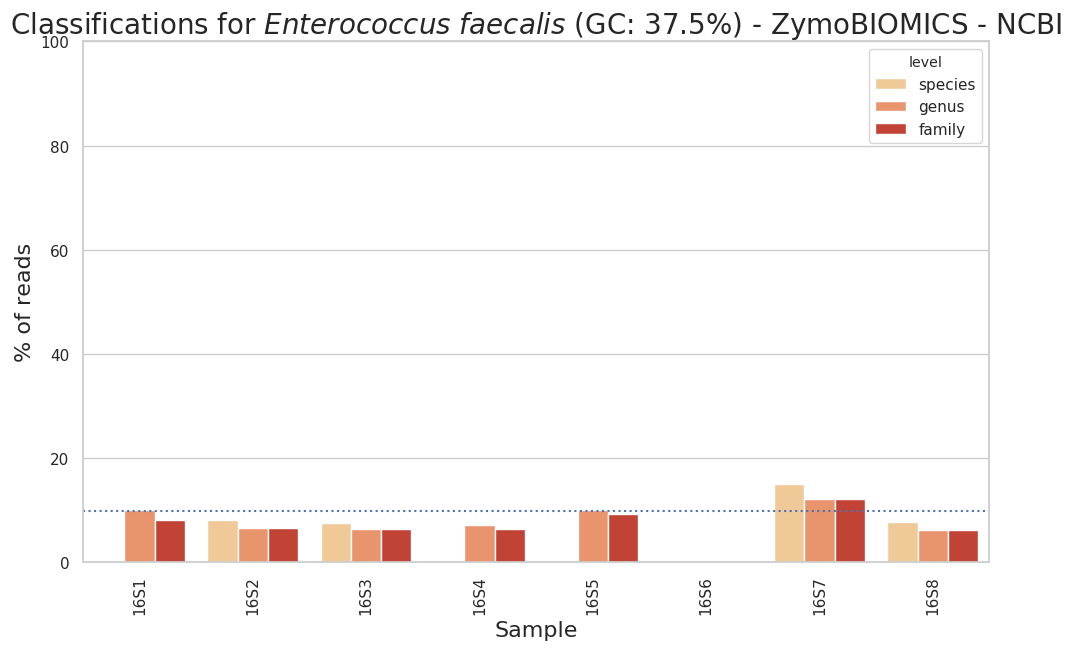

Supplement: Supplementary file 1 [file ijms-21-00298-s001.zip › ijms-659682-proofreading si/figures/Zymo_Mock_Enterococcus_ncbi_16s.png]

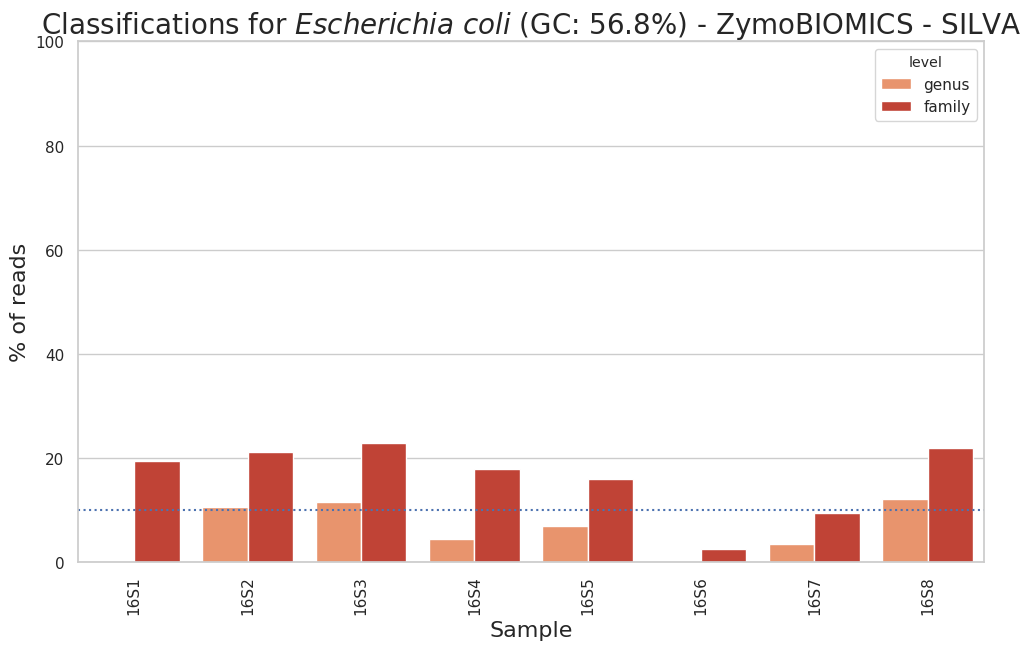

Supplement: Supplementary file 1 [file ijms-21-00298-s001.zip › ijms-659682-proofreading si/figures/Zymo_Mock_Escherichia-Shigella_mothur_silva.png]

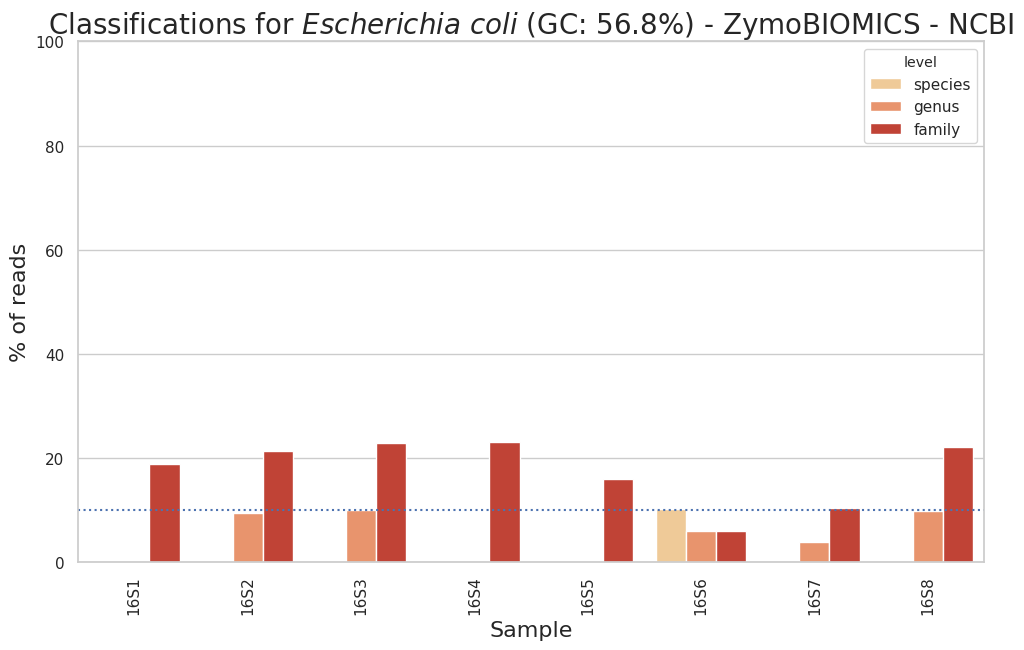

Supplement: Supplementary file 1 [file ijms-21-00298-s001.zip › ijms-659682-proofreading si/figures/Zymo_Mock_Escherichia-Shigella_ncbi_16s.png]

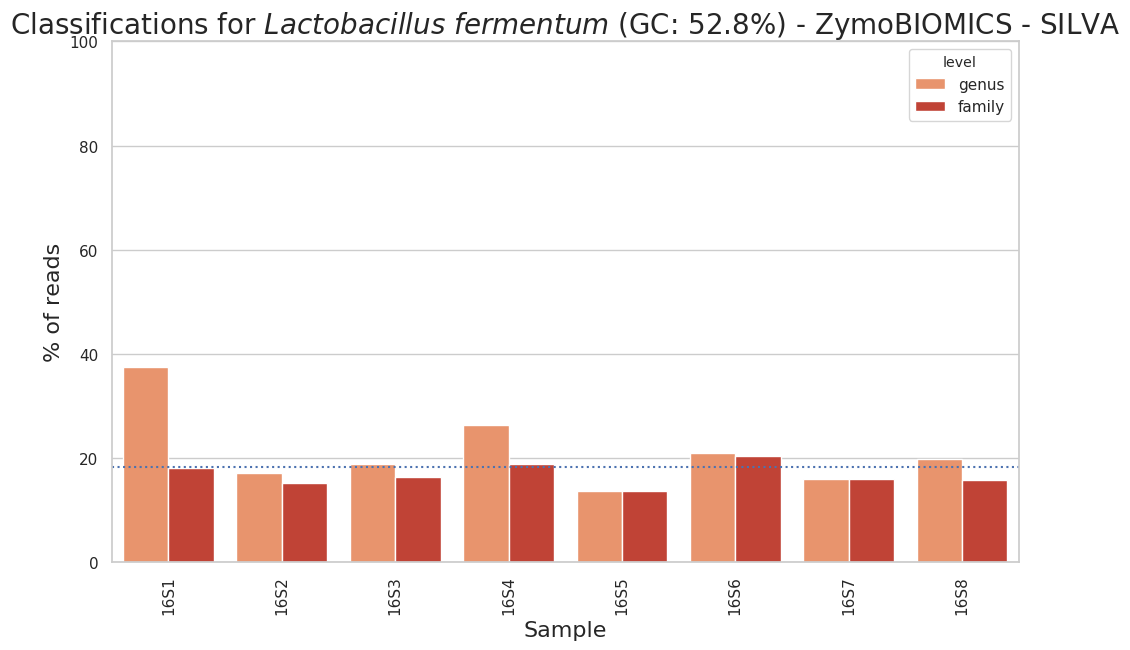

Supplement: Supplementary file 1 [file ijms-21-00298-s001.zip › ijms-659682-proofreading si/figures/Zymo_Mock_Lactobacillus_mothur_silva.png]

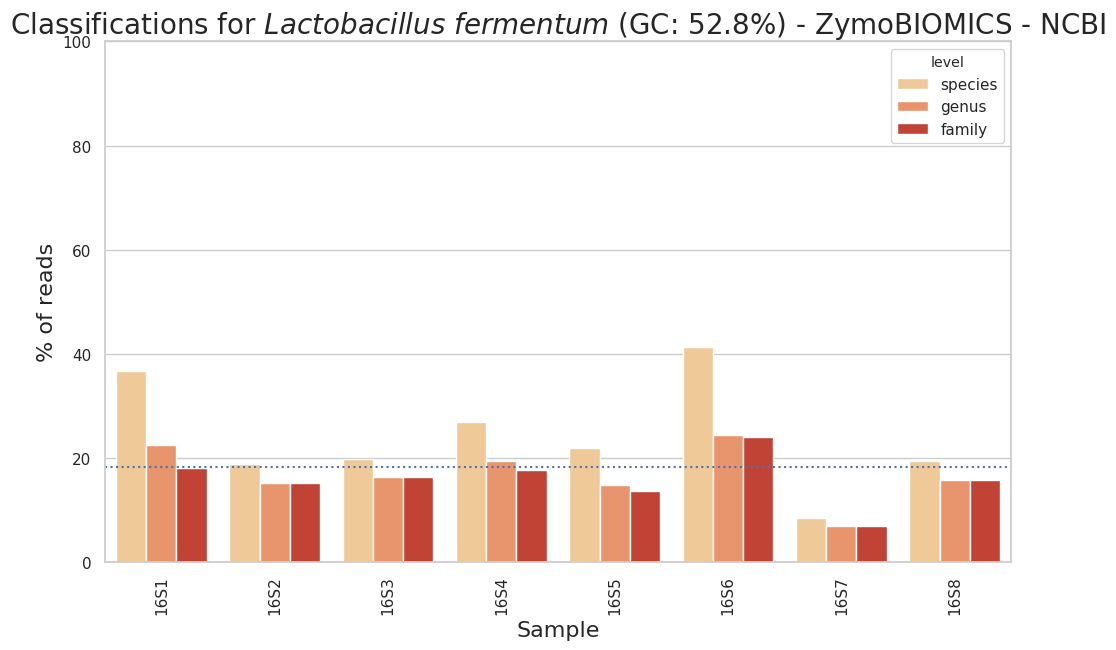

Supplement: Supplementary file 1 [file ijms-21-00298-s001.zip › ijms-659682-proofreading si/figures/Zymo_Mock_Lactobacillus_ncbi_16s.png]

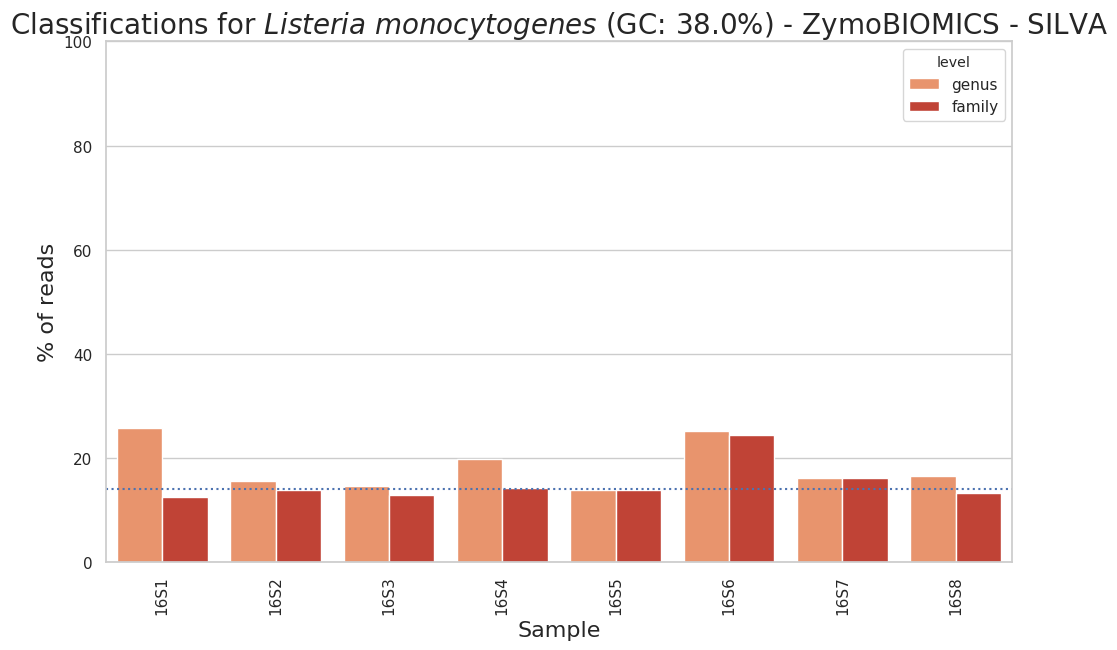

Supplement: Supplementary file 1 [file ijms-21-00298-s001.zip › ijms-659682-proofreading si/figures/Zymo_Mock_Listeria_mothur_silva.png]

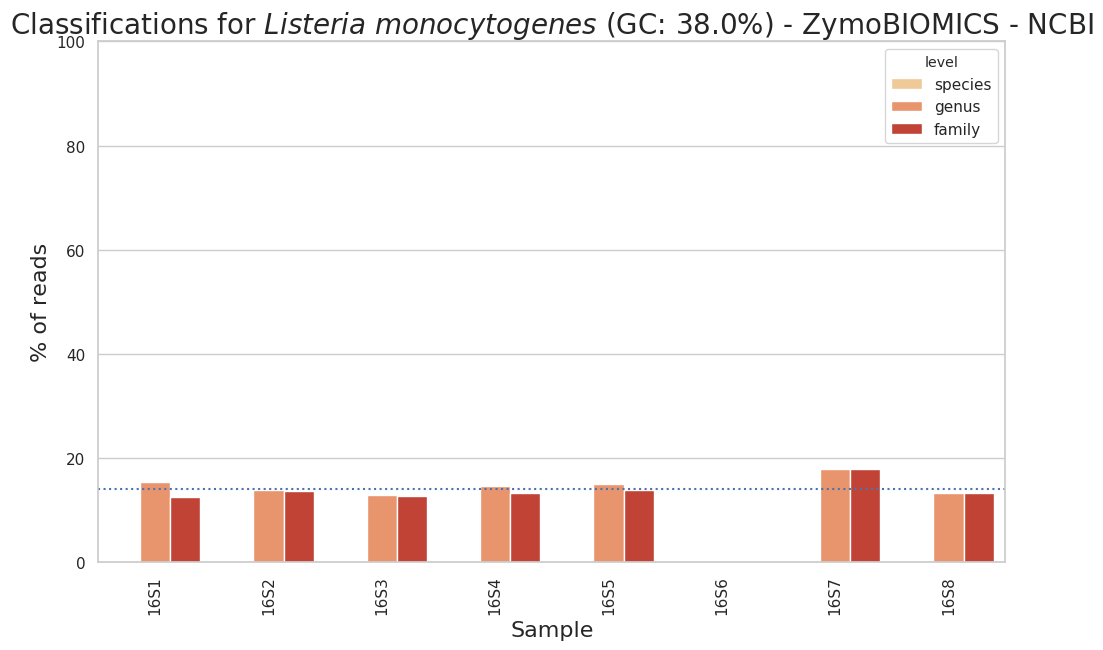

Supplement: Supplementary file 1 [file ijms-21-00298-s001.zip › ijms-659682-proofreading si/figures/Zymo_Mock_Listeria_ncbi_16s.png]

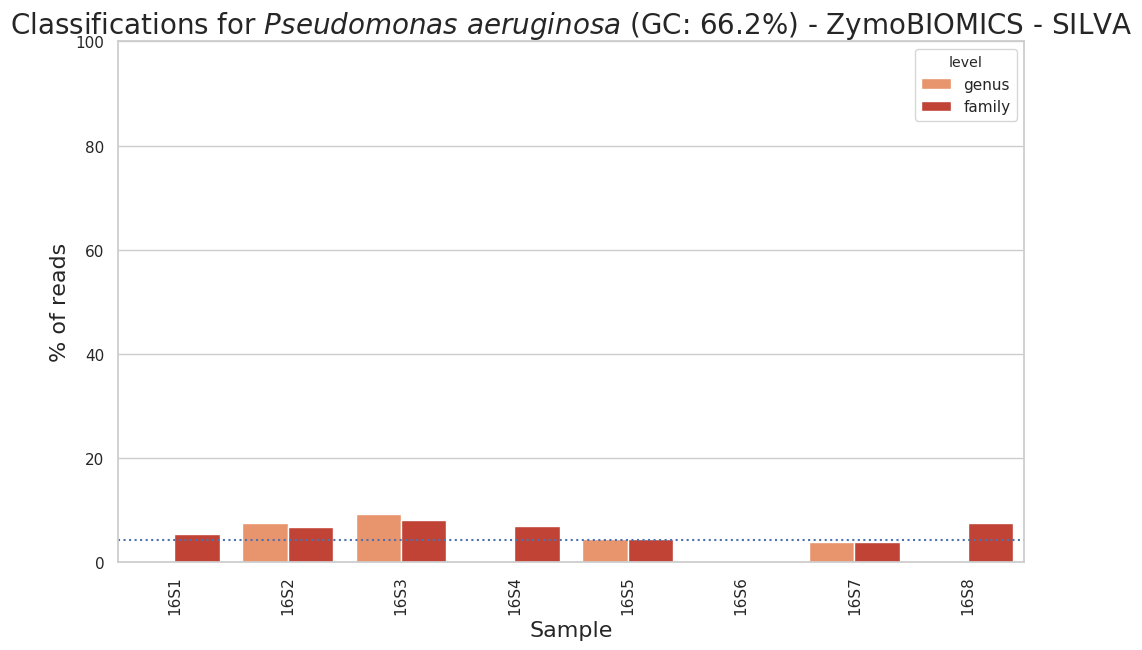

Supplement: Supplementary file 1 [file ijms-21-00298-s001.zip › ijms-659682-proofreading si/figures/Zymo_Mock_Pseudomonas_mothur_silva.png]

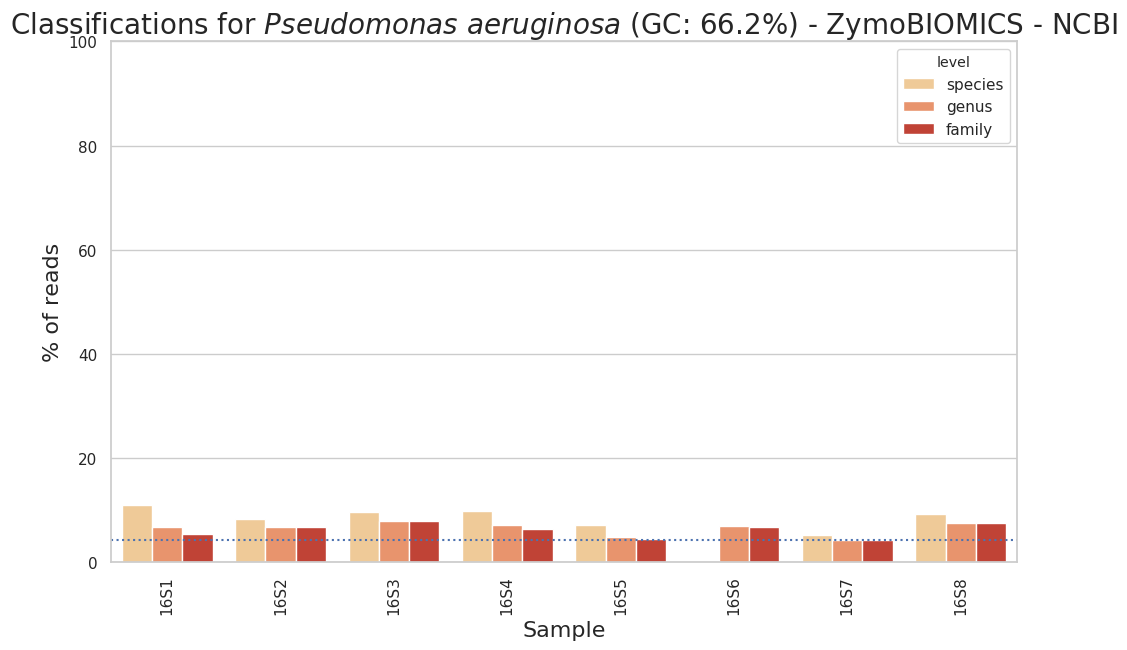

Supplement: Supplementary file 1 [file ijms-21-00298-s001.zip › ijms-659682-proofreading si/figures/Zymo_Mock_Pseudomonas_ncbi_16s.png]

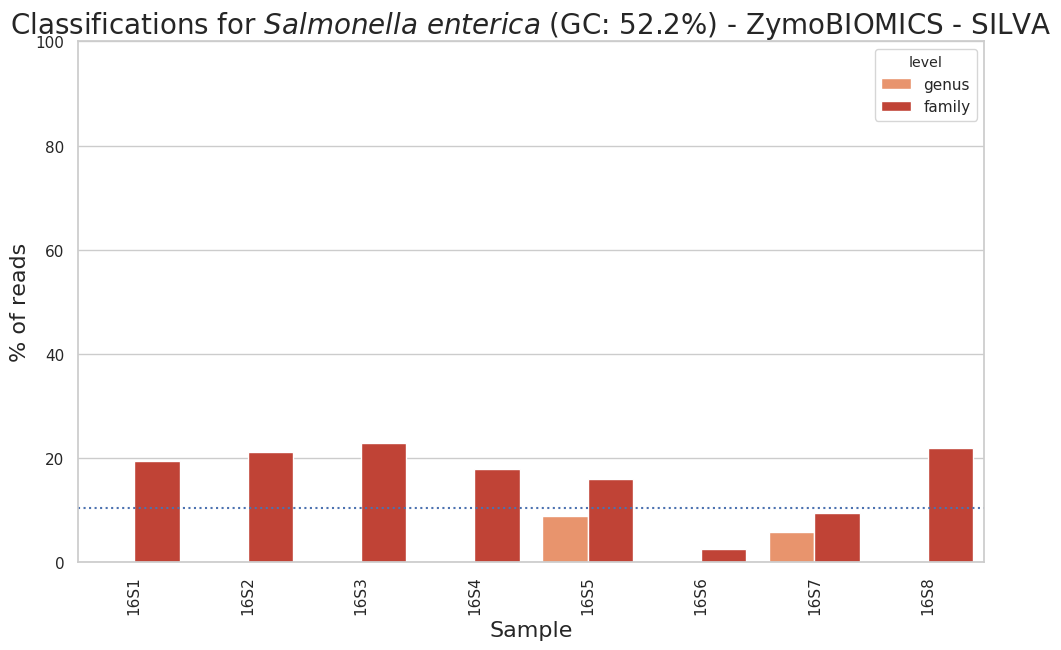

Supplement: Supplementary file 1 [file ijms-21-00298-s001.zip › ijms-659682-proofreading si/figures/Zymo_Mock_Salmonella_mothur_silva.png]

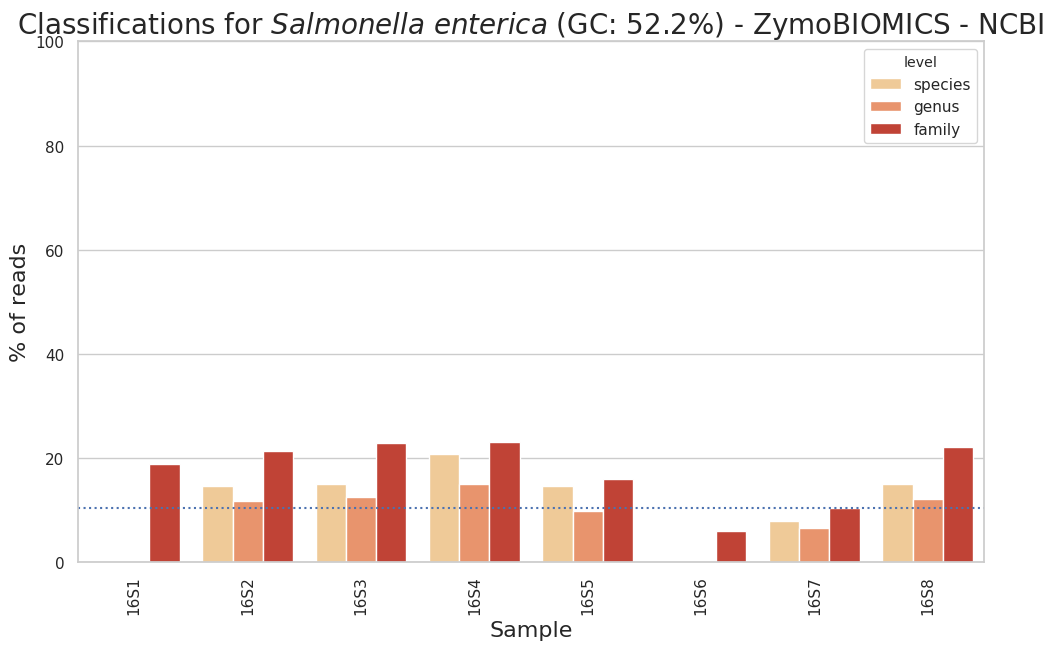

Supplement: Supplementary file 1 [file ijms-21-00298-s001.zip › ijms-659682-proofreading si/figures/Zymo_Mock_Salmonella_ncbi_16s.png]

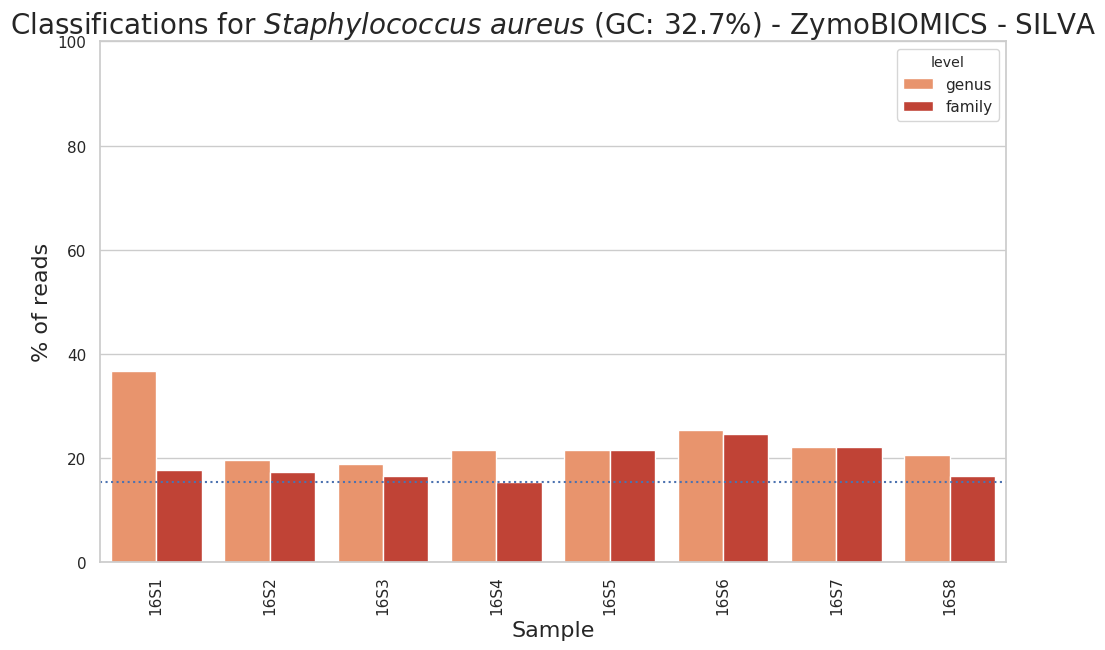

Supplement: Supplementary file 1 [file ijms-21-00298-s001.zip › ijms-659682-proofreading si/figures/Zymo_Mock_Staphylococcus_mothur_silva.png]

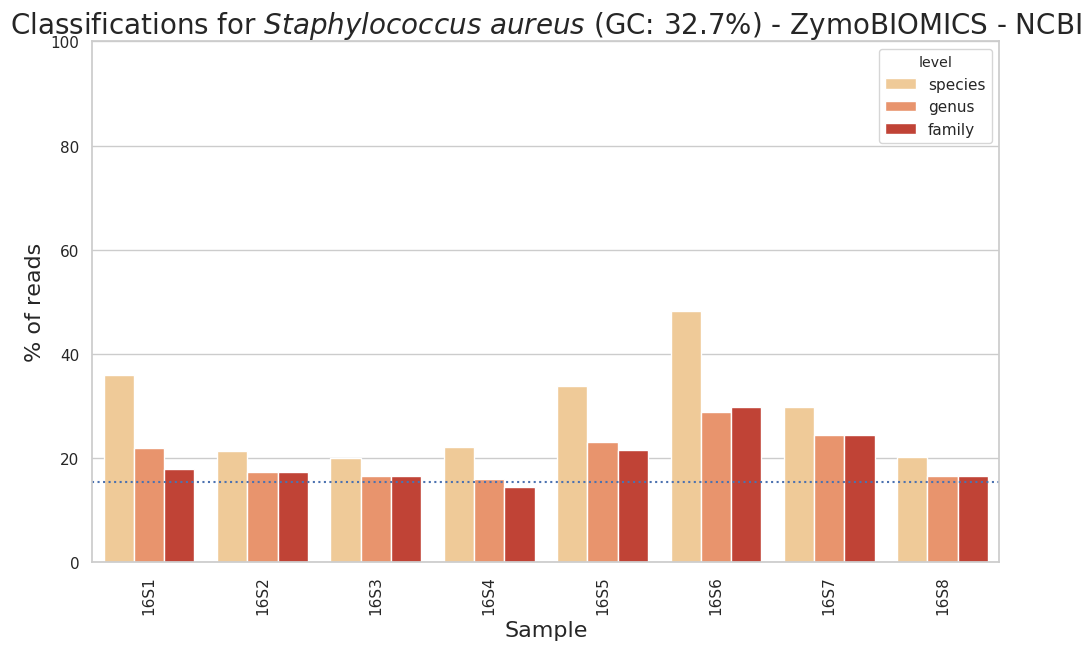

Supplement: Supplementary file 1 [file ijms-21-00298-s001.zip › ijms-659682-proofreading si/figures/Zymo_Mock_Staphylococcus_ncbi_16s.png]
